# Supplementary material for: Metagenome mining and functional analysis reveal oxidized guanine DNA repair at the Lost City Hydrothermal Field
Source: PLoS One. 2024 May 8;19(5):e0284642. doi: 10.1371/journal.pone.0284642 (PMC11078426; doi:10.1371/journal.pone.0284642)
Supplement: S1 Fig — Structural homology for Ec YggX (left) and nearest neighbor to Thiotrichaceae MutY (right). The solution NMR structure for Ec YggX (PDB ID 1yhd) [103] is superimposable to the structure predicted by Colabfold for the nearest neighbor to Thiotrichaceae MutY, with RMSD of 0.99 Å for 69 pruned pairs selected from 88 possible pairs. The Cys residue critical for function is highlighted with a yellow sphere for the sulfhydryl group. (PDF) [file pone.0284642.s001.pdf]

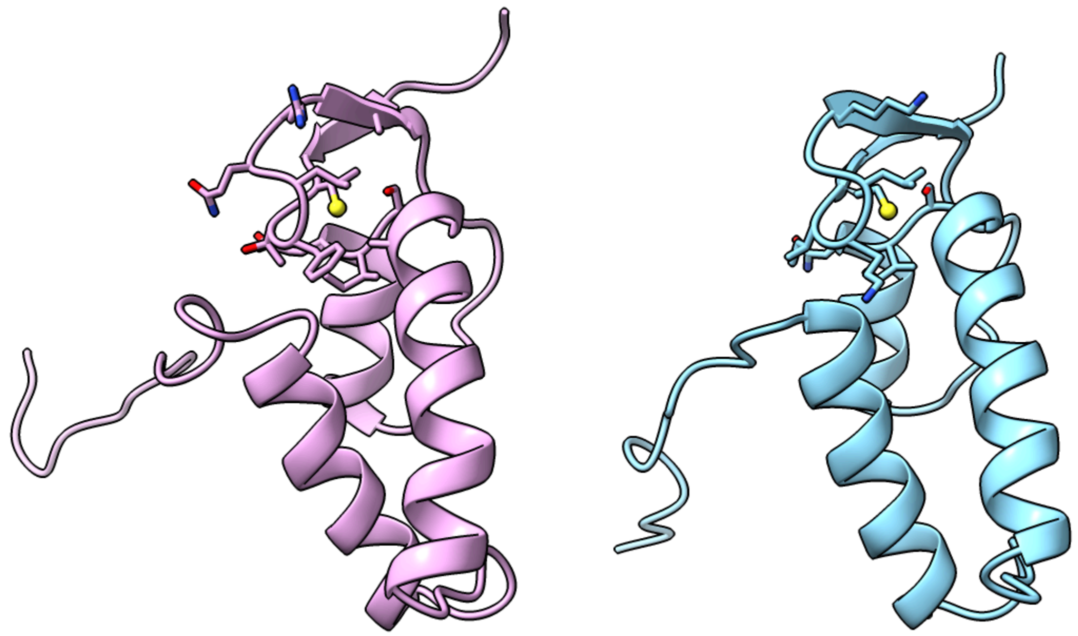

S3 Fig. MutY gene neighbor YggX.
Structural homology for *Ec* YggX (left) and nearest neighbor to *Thiotrichaceae* MutY (right). The solution NMR structure for *Ec* YggX (PDB ID 1yhd) [103] is superimposable to the structure predicted by *Colabfold* for the nearest neighbor to *Thiotrichaceae* MutY, with RMSD of 0.99 Å for 69 pruned pairs selected from 88 possible pairs. The Cys residue critical for function is highlighted with a yellow sphere for the sulfhydryl group.
