# Supplementary material for: Metagenome mining and functional analysis reveal oxidized guanine DNA repair at the Lost City Hydrothermal Field
Source: PLoS One. 2024 May 8;19(5):e0284642. doi: 10.1371/journal.pone.0284642 (PMC11078426; doi:10.1371/journal.pone.0284642)
Supplement: S2 Fig — pLDDT scores represent the confidence in the prediction calculated by Colabfold. (PDF) [file pone.0284642.s002.pdf]

S5 Fig. Colabfold structure prediction pLDDT scores
pLDDT scores represent the confidence in the prediction calculated by *Colabfold*.

Marinosulfonomonas MutY Structure Prediction

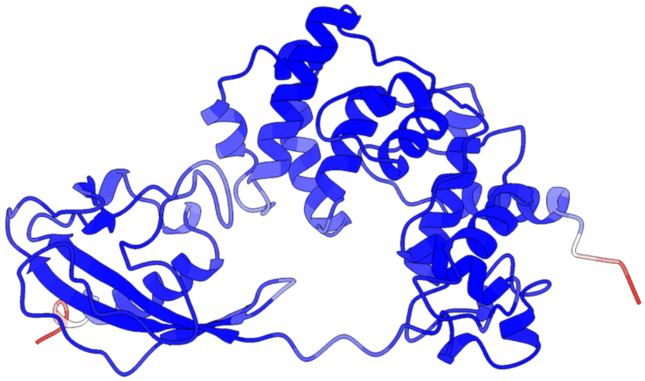

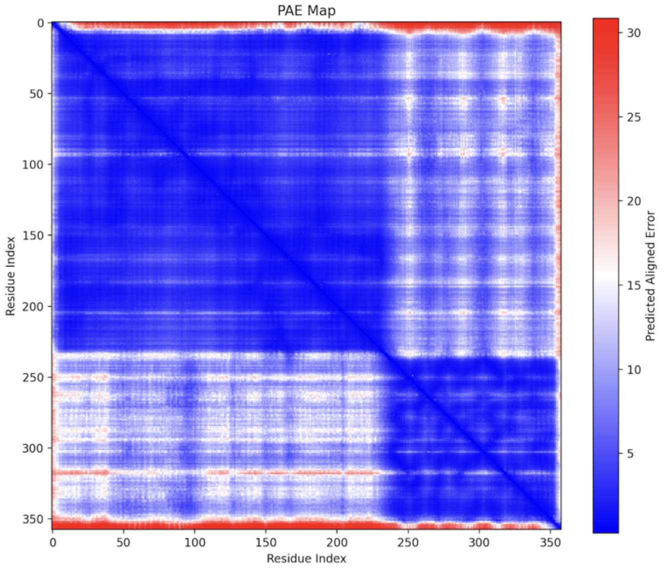

pLDDT score: 93 (±9)
ptm score: 0.75

Rhodobacteraceae MutY Structure Prediction

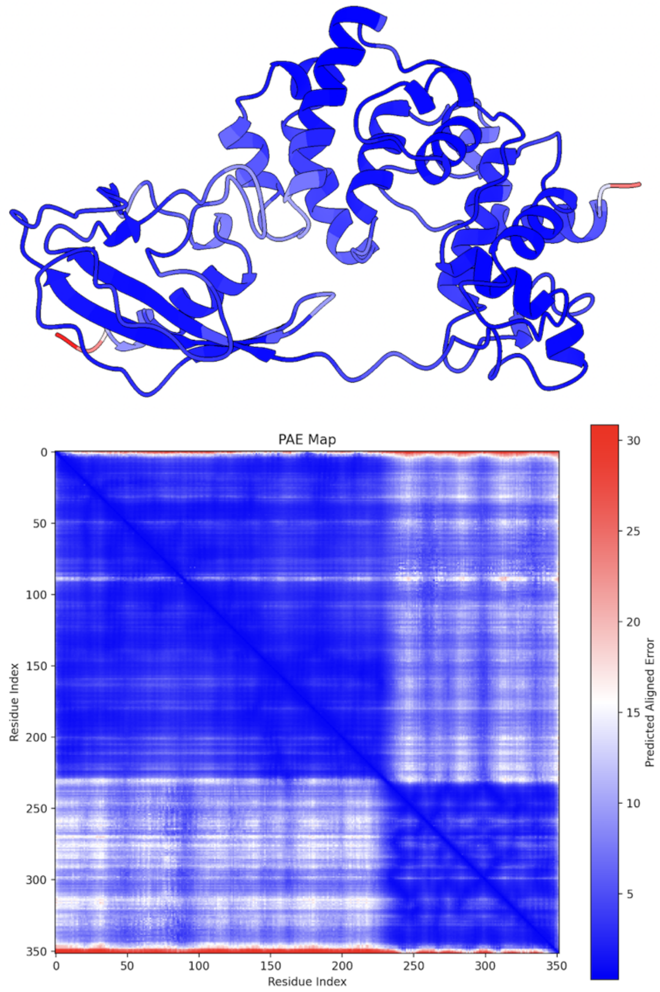

pLDDT score: 94 (±6)
ptm score: 0.79

Thiotrichaceae MutY Structure Prediction

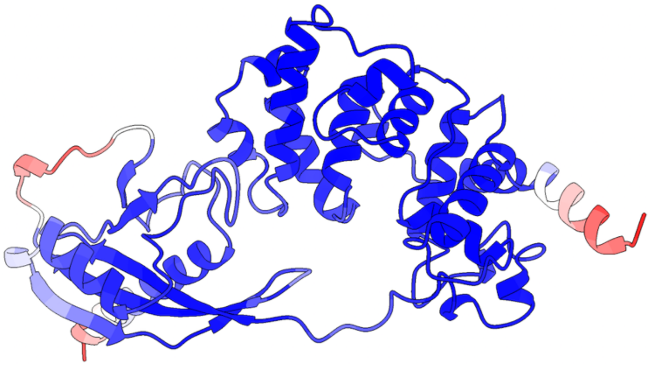

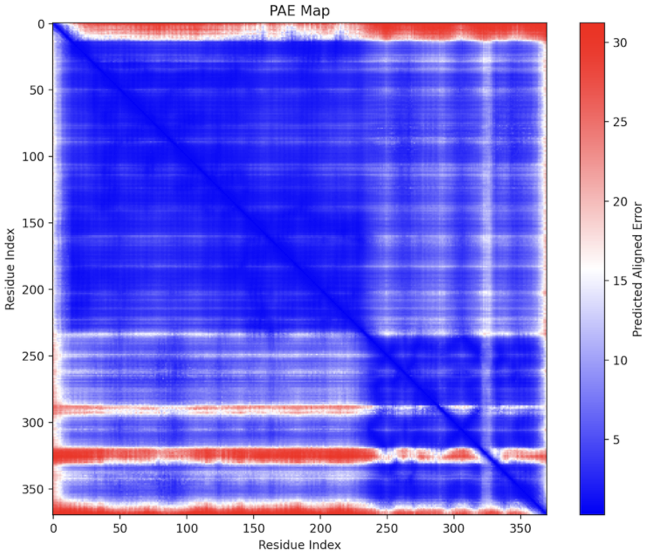

pLDDT score: 92 (±12)
ptm score: 0.83

Flavobacteriaceae MutY Structure Prediction

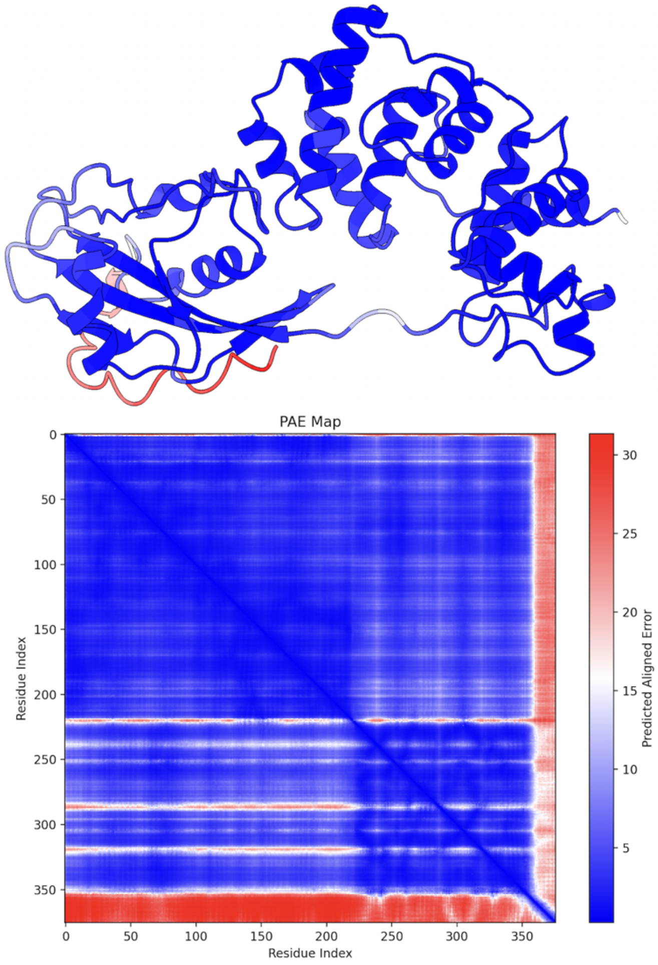

pLDDT score: 92 (±14)
ptm score: 0.84
