# Supplementary material for: Metagenome mining and functional analysis reveal oxidized guanine DNA repair at the Lost City Hydrothermal Field
Source: PLoS One. 2024 May 8;19(5):e0284642. doi: 10.1371/journal.pone.0284642 (PMC11078426; doi:10.1371/journal.pone.0284642)

S7 Fig. Molecular dynamics.

Molecular dynamics simulations were calculated by *GROMACS* with the *Amber*99SB and GAFF force fields for MutY complexed to adenosine and OG. For each MD simulation, short range interaction energies, distances between the ligand and functionally relevant residues, and representative structures sampled after equilibration and at 10,000 ps are shown. Note that the Y-axis is logarithmic for distance. The adenosine and OG ligands are shown with all atoms wrapped in transparent surfaces. For the adenosine complexes, the protein structure was truncated so as to focus on the NTD (residues 8 - 220 in *Gs* MutY, and corresponding residues for the LCHF MutYs). Catalytic residues are shown: Glu43 and Asp144 in the *Gs* MutY protein and corresponding residues in the LCHF MutYs. The distance versus time plot for the adenosine complex, tracks potential contacts between the catalytic Glu (atoms OE1 and OE2) and the hydrogen bond donors and acceptors on adenosine (atoms N1, N6 and N7). For the OG complex, the iron-sulfur cluster domain and inter-domain linker were omitted so as to focus on the OG-recognition site found at the interface between NTD (residues 29-137 in *Gs* MutY) and CTD (residues 234-360 in *Gs* MutY). Residues that interact with OG are shown: Thr49, and Ser308 in the *Gs* MutY protein and corresponding residues in the LCHF MutYs. The distance versus time plot for the OG complex tracks potential contacts between the critical Ser/Thr residues and the hydrogen bond donors and acceptors on OG (atoms N1, N2, O6, N7 and O8). The total short range interaction energy (black trace) is the sum of short range Leanord-Jones (salmon trace) and Coulombic (sky blue) interaction energies.

(A) Molecular dynamic simulation for *Gs* MutY NTD complexed with adenosine. The ligand complex persisted for the entire 100,000 ps, with changes in location and orientation evident at 16,000 ps and 42,000 ps in the distance plot. Hydrogen bonds between catalytic Glu43 and the Hoogsten face of the adenine base were observed during the first 16,000 ps. These consistently involved direct contact with N6, as evidenced by close distance (green traces) and inspection of structures. N7 was also engaged (blue traces), with relevance for catalysis, via bridging water molecules (O red and H white).

(B) Molecular dynamic simulation for *Gs* MutY complexed with OG. The ligand complex was stable for 92,000 ps, with the OG ligand bound to a cleft between the NTD (white) and CTD (gray). The functionally relevant hydrogen bond between the amide N of Ser308 and atom O8 of OG was frequently observed (not shown), sometimes accompanied by a second OG-specific hydrogen bond between the hydroxyl of Ser308 and atom N7 of OG (sky blue trace in the distance plot).

(C) Molecular dynamic simulation for *Marinosulfonomonas* MutY NTD complexed with adenosine. In the first 3,000 ps, the adenine base approached closely catalytic Glu49 (green traces), often directly hydrogen bonded and occasionally bridged by a solvent molecule. However, the complex was relatively unstable, and the ligand departed the active site and found a new binding site by 8,000 ps. Favorable VDW interactions characterize both binding sites, but favorable Coulombic interactions are diminished substantially at the second site.

(D) Molecular dynamic simulation for *Marinosulfonomonas* MutY complexed with OG. The initial ligand complex was unstable with a hinge-like motion creating new contacts between the NTD (white) and CTD (gray). After nearly escaping at ~4,000 ps, the OG ligand found several alternate sites on the NTD and CTD.

(E) Molecular dynamic simulation for *Rhodobacteraceae* MutY NTD complexed with adenosine. The complex was relatively unstable. The adenine base initially hydrogen bonded with catalytic Glu45 during the first 3,800 ps but then changed orientation and drifted to a new site distinct and different from its original docking site. Note, catalytic Glu45 is not visible in the 10,000-ps representative structure as the new position of adenosine blocks its view.

(F) Molecular dynamic simulation for *Rhodobacteraceae* MutY complexed with OG. The ligand complex was unstable and dissociated completely within 48 ns. Functionally relevant hydrogen bonds between Thr299 and OG observed for the initially equilibrated structure were lost as the ligand moved to new positions on the NTD and CTD before dissociation.

(G) Molecular dynamic simulation for *Thiotrichaceae* MutY NTD complexed with adenosine. Note that Ser replaces active site Tyr for this LCHF MutY, as is also the case for *Ec* MutY. The complex was relatively stable with the ligand persisting in the active site throughout the simulation. Hydrogen bonds between catalytic Glu46 and the Hoogsteen face of adenine were evident by close distance to N7 (blue traces) and N6 (green traces) and by inspection of structures. Water frequently bridged N7 to Glu46 as seen in the representative structure at 10,000 ps.

(H) Molecular dynamic simulation for *Thiotrichaceae* MutY complexed with OG. The ligand complex was stable for the entire 100,000-ps simulation with the OG ligand bound to a cleft between the NTD (white) and CTD (gray). Hydrogen bonds between Ser306 and OG were frequently observed.

(I) Molecular dynamic simulation for *Flavobacteriaceae* MutY NTD complexed with adenosine. The ligand persisted in the active site throughout the simulation, periodically finding new orientations as evident in different distance traces vying for close approach to catalytic Glu33. For example, N7 of the adenine base was very close to Glu33 (blue trace) during the first 2,700 ps, suggesting catalytic engagement, but slipped out of reach at later time points. Water frequently bridged contacts between Glu33 and the adenine base.

(J) Molecular dynamic simulation for *Flavobacteriaceae* MutY complexed with OG. The ligand complex was relatively stable. Functionally relevant hydrogen bonds between Ser305 and the Hoogsten face of OG can be inferred from recurring close distances up until 13,000 ps when the ligand adopts a new pose at the NTD-CTD interface.

(A) *Geobacillus stearothermophilus* MutY NTD complexed with adenosine

Distance Adenosine - Glu43

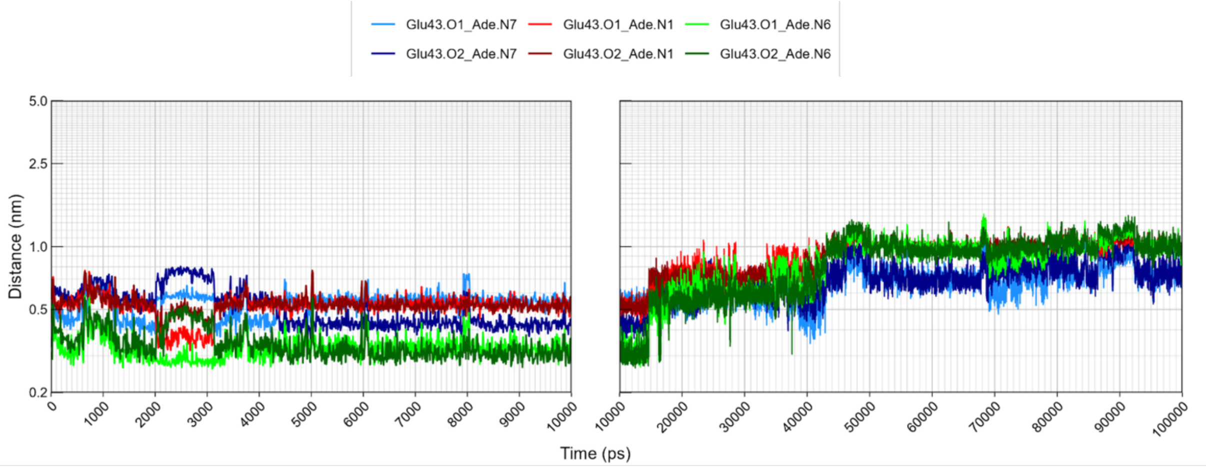

Energy

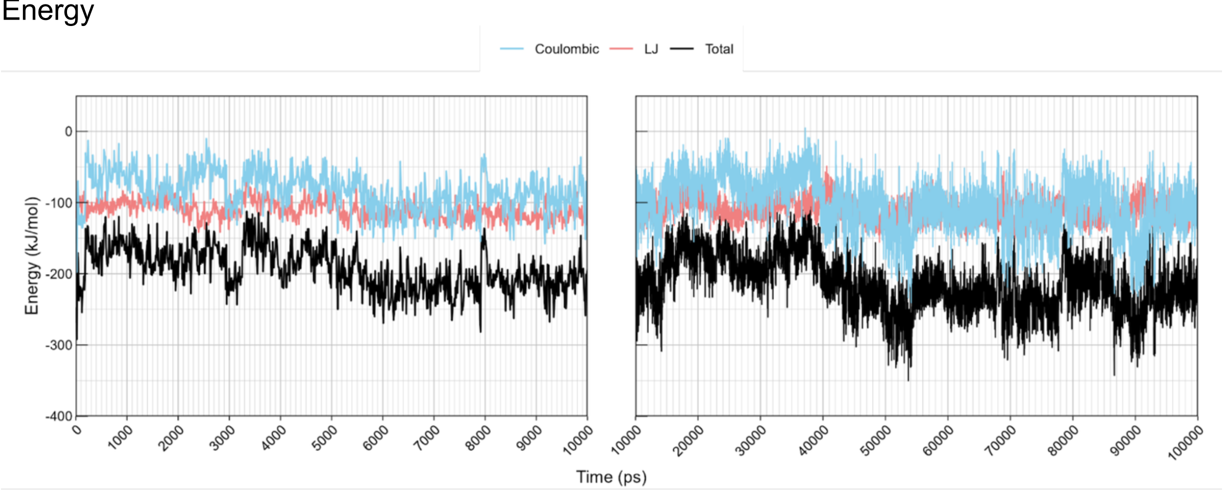

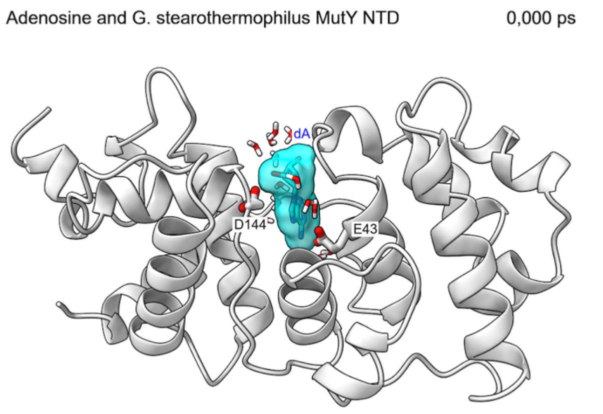

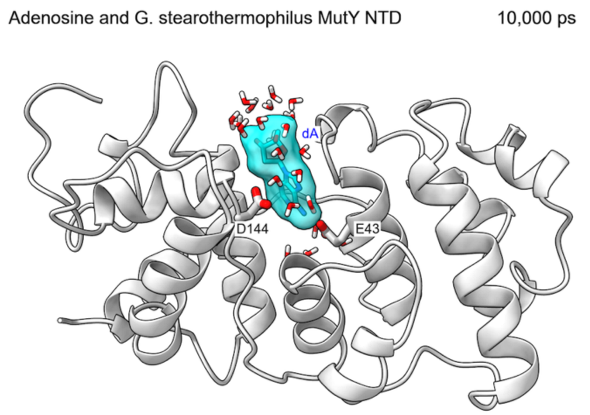

(B) *Geobacillus stearothermophilus* MutY complexed with OG

Distance OG - Thr49 and Ser308

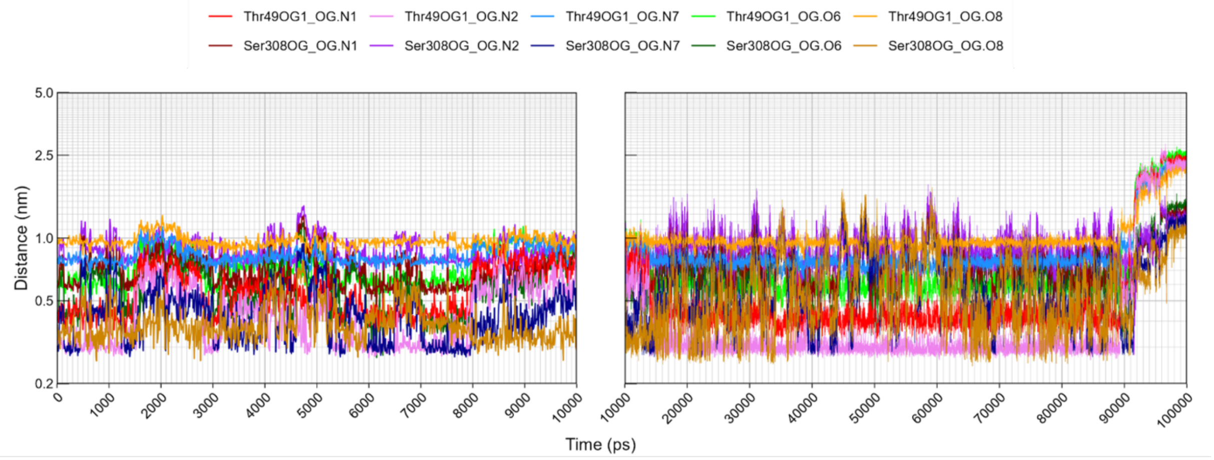

Energy

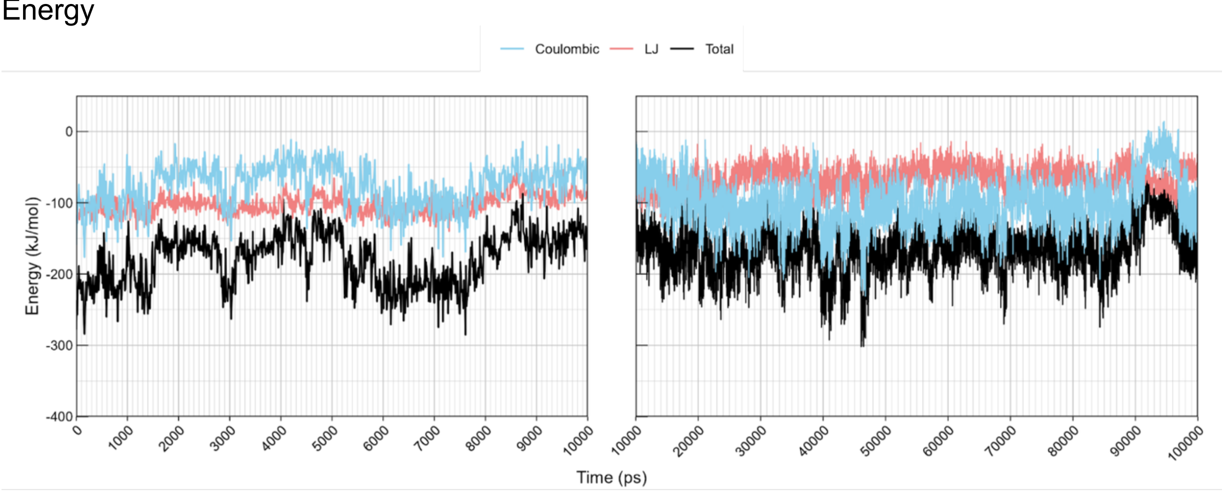

OG and Geobacillus stearothermophilus MutY 0,000 ps

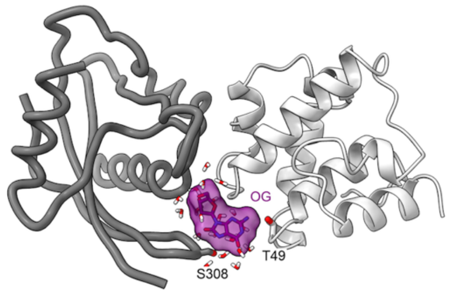

OG and Geobacillus stearothermophilus MutY 10,000 ps

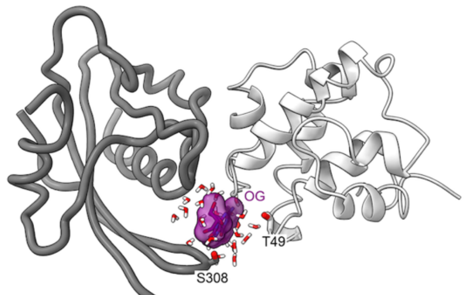

(C) *Marinosulfonomonas* MutY NTD complexed with adenosine

Distance Adenosine - Glu49

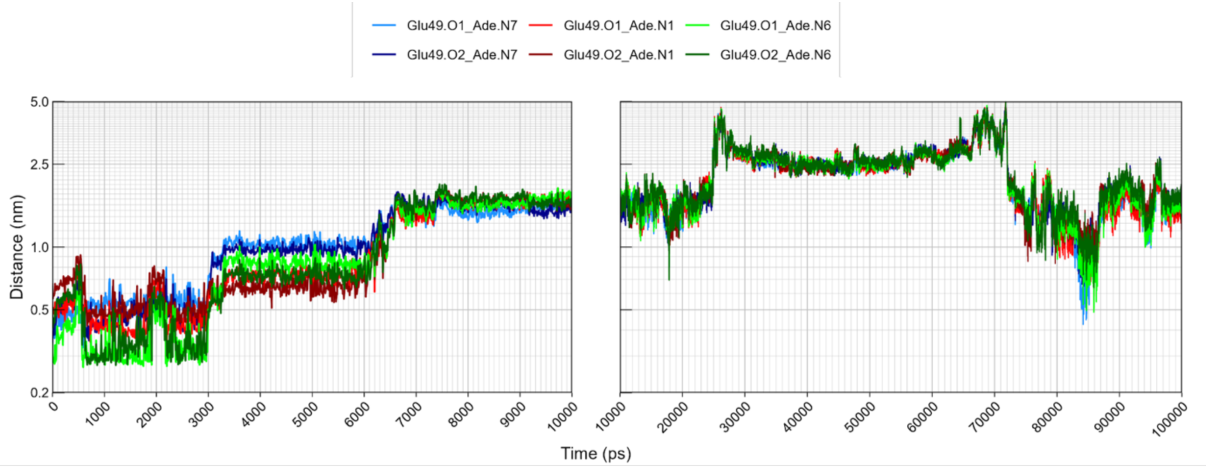

Energy

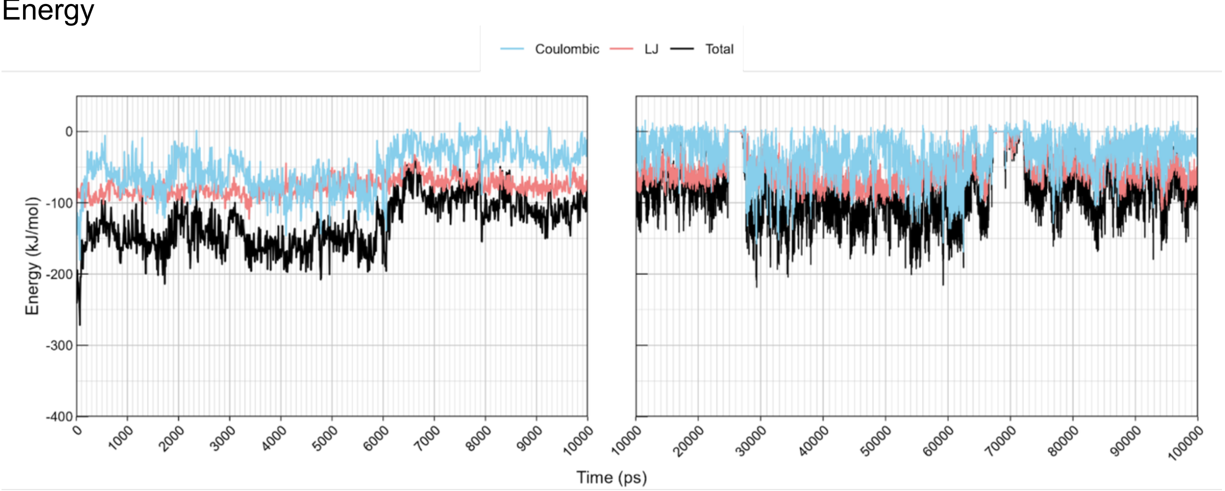

Adenosine and Marinosulfonomonas MutY NTD 0,000 ps

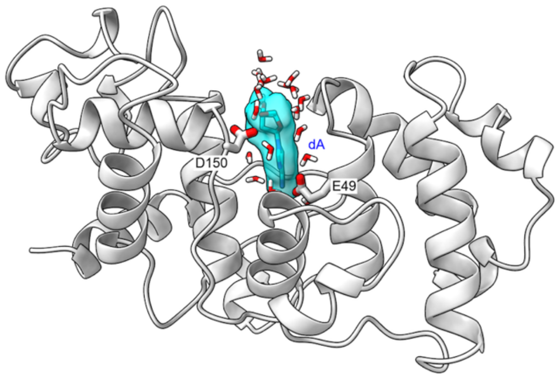

Adenosine and Marinosulfonomonas MutY NTD 10,000 ps

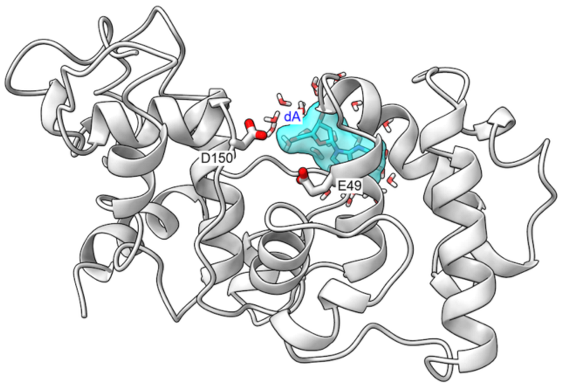

(D) *Marinosulfonomonas* MutY complexed with OG

Distance OG - Thr55 and Thr303

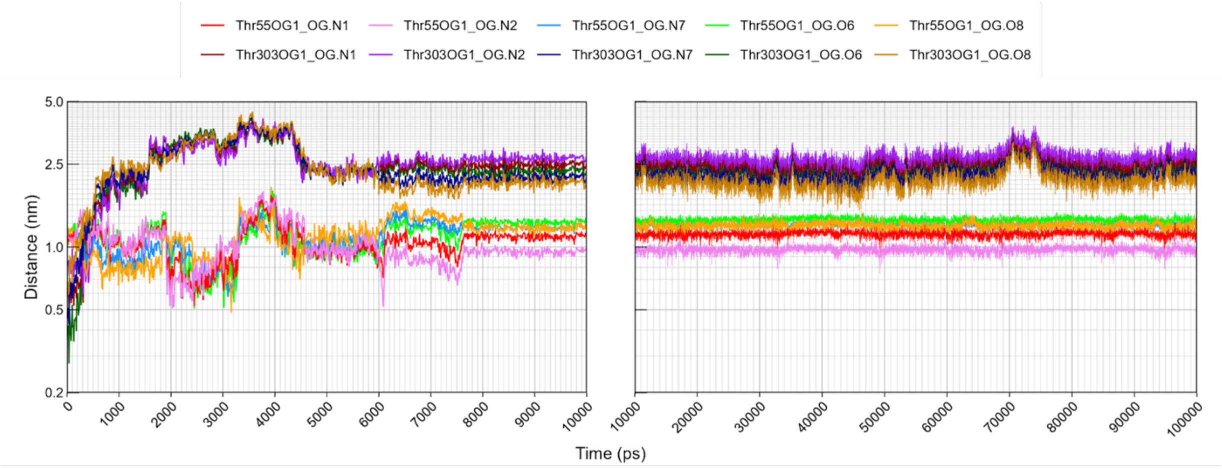

Energy

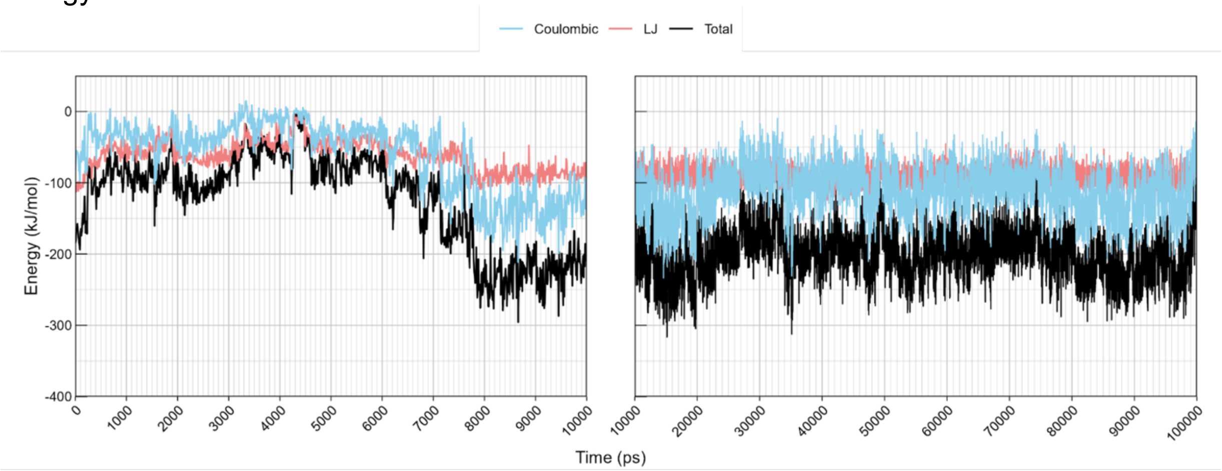

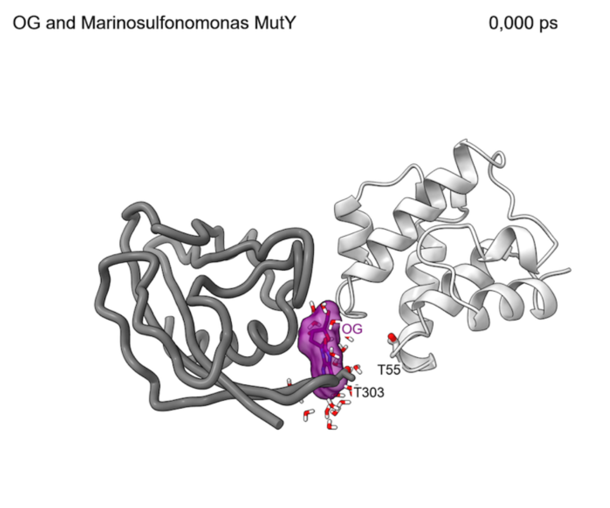

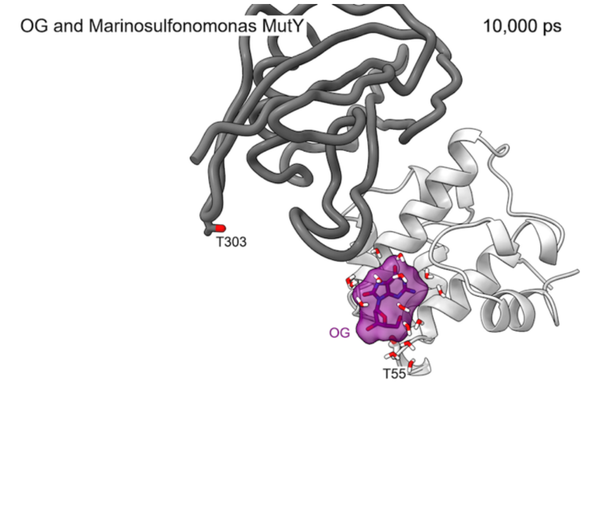

(E) *Rhodobacteraceae* MutY NTD complexed with adenosine

Distance Adenosine - Glu45

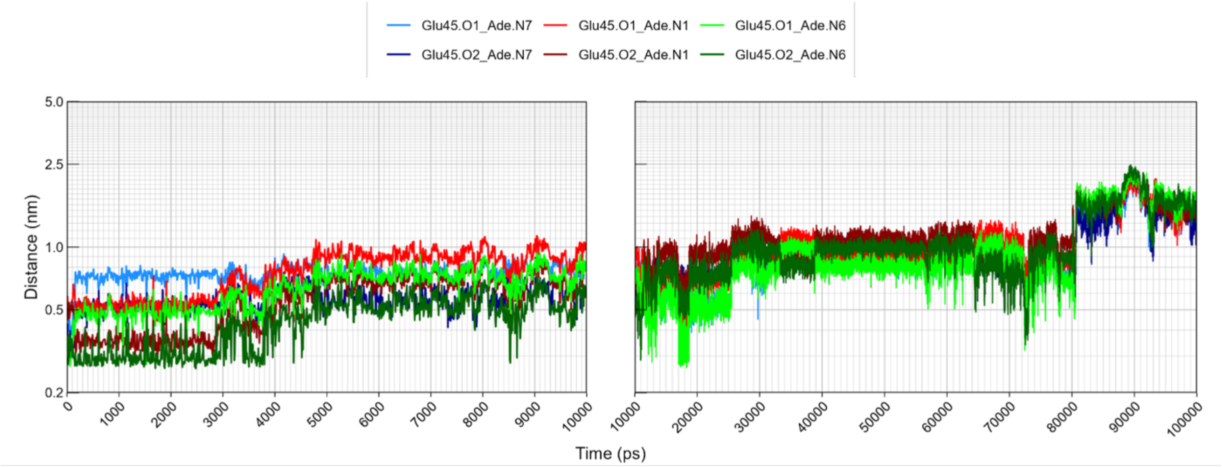

Energy

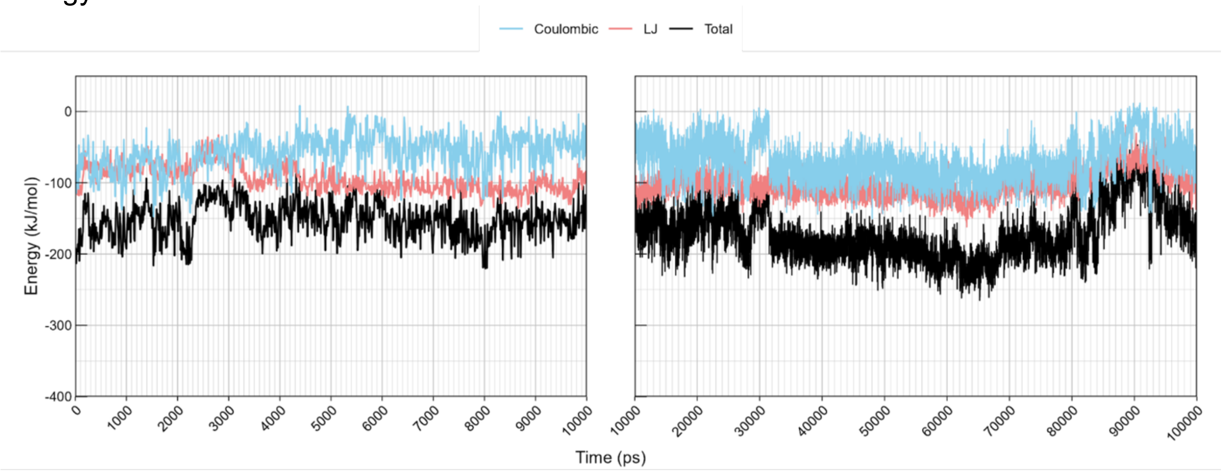

Adenosine and Rhodobacteraceae MutY NTD 0,000 ps

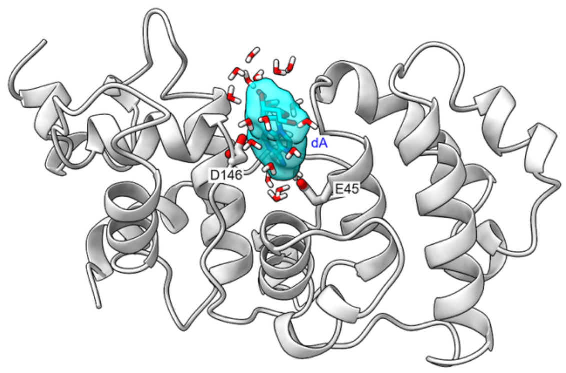

Adenosine and Rhodobacteraceae MutY NTD 10,000 ps

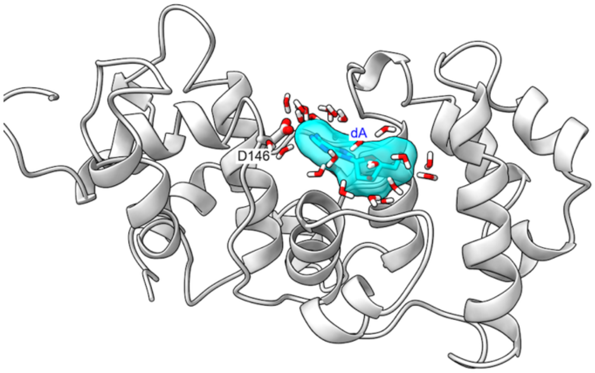

(F) *Rhodobacteraceae* MutY complexed with OG

Distance OG - Thr51 and Thr299

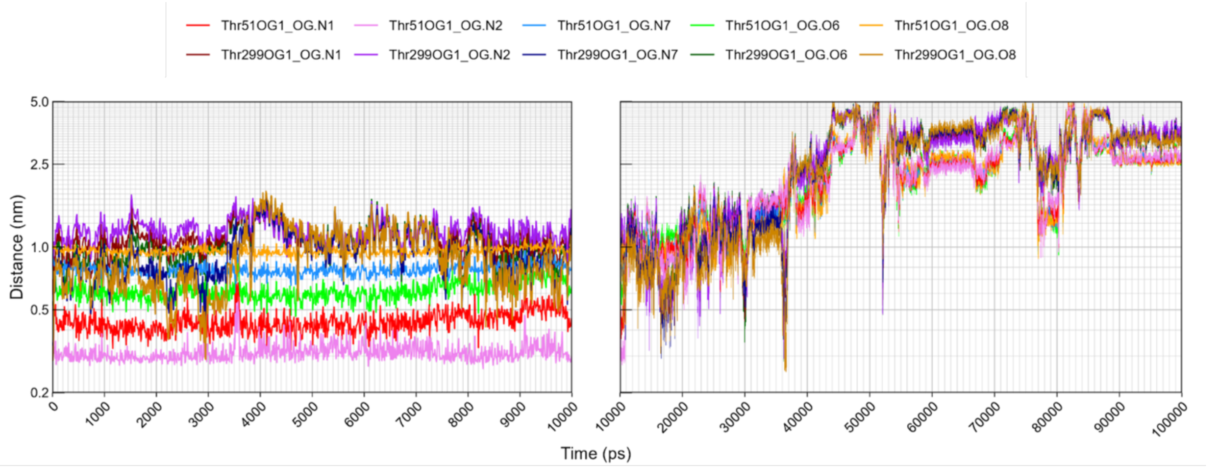

Energy

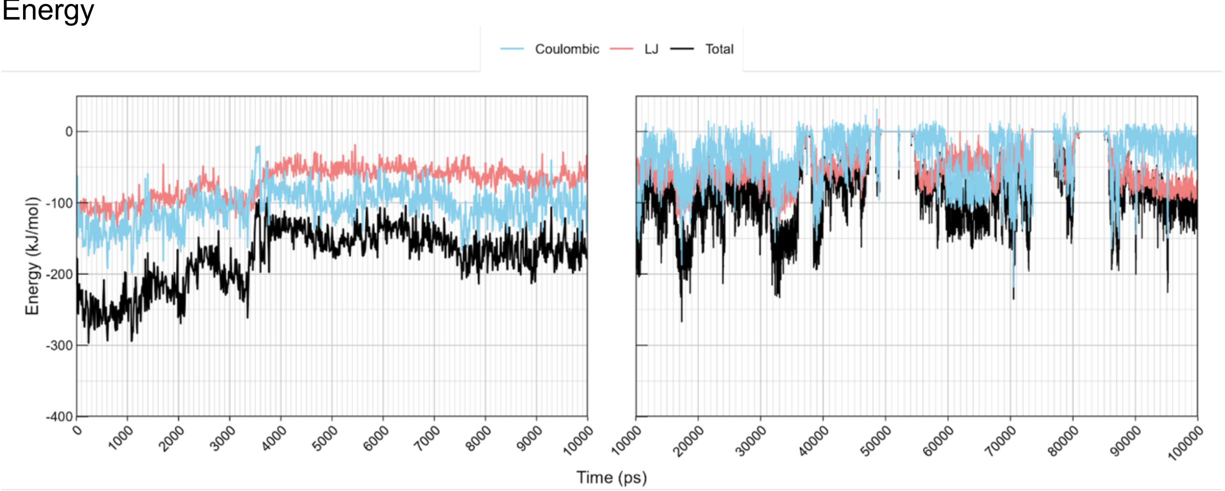

OG and Rhodobacteraceae MutY 0,000 ps

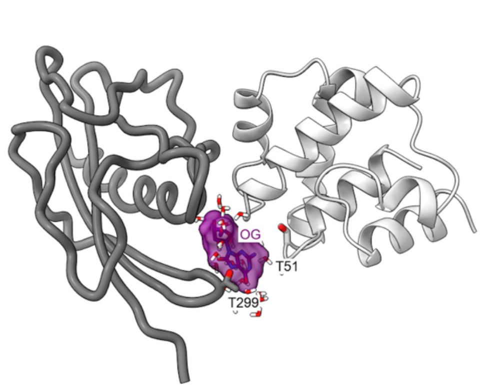

OG and Rhodobacteraceae MutY 10,000 ps

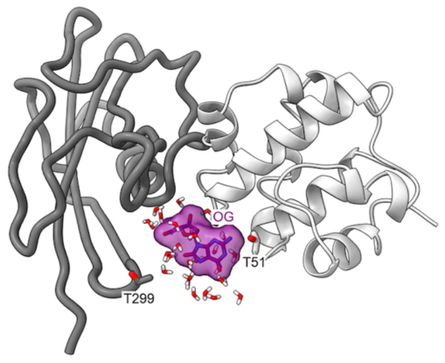

(G) *Thiotrichaceae* MutY NTD complexed with adenosine

Distance Adenosine - Glu46

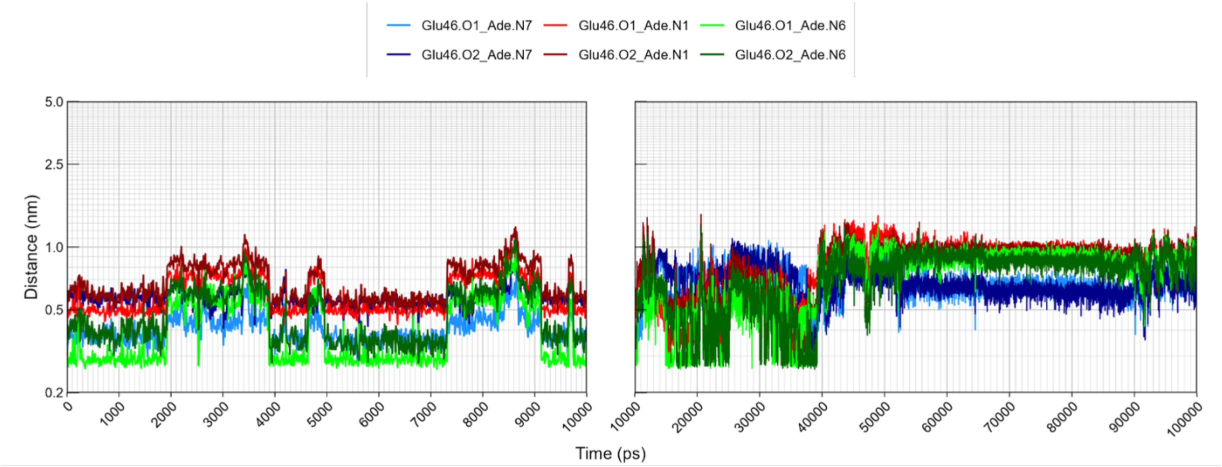

Energy

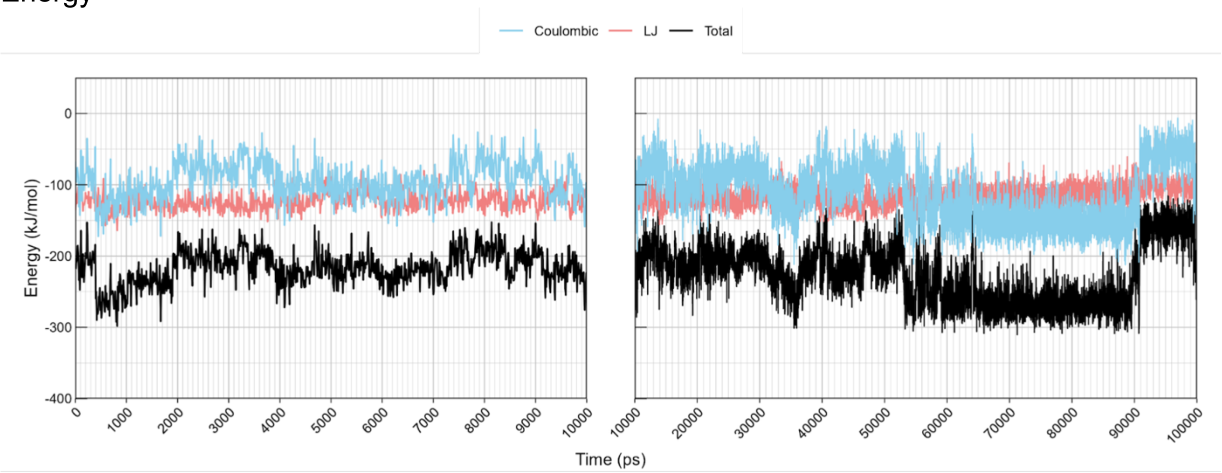

Adenosine and Thiotrichaceae MutY NTD 0,000 ps

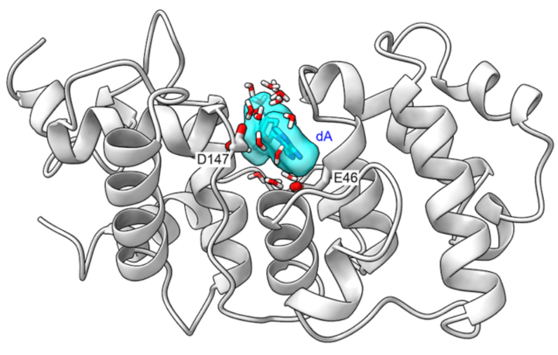

Adenosine and Thiotrichaceae MutY NTD 10,000 ps

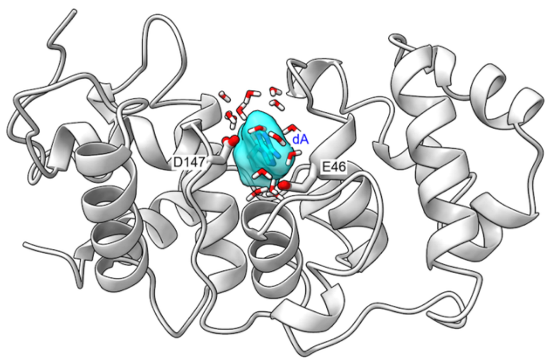

(H) *Thiotrichaceae* MutY complexed with OG

Distance OG - Thr52 and Ser306

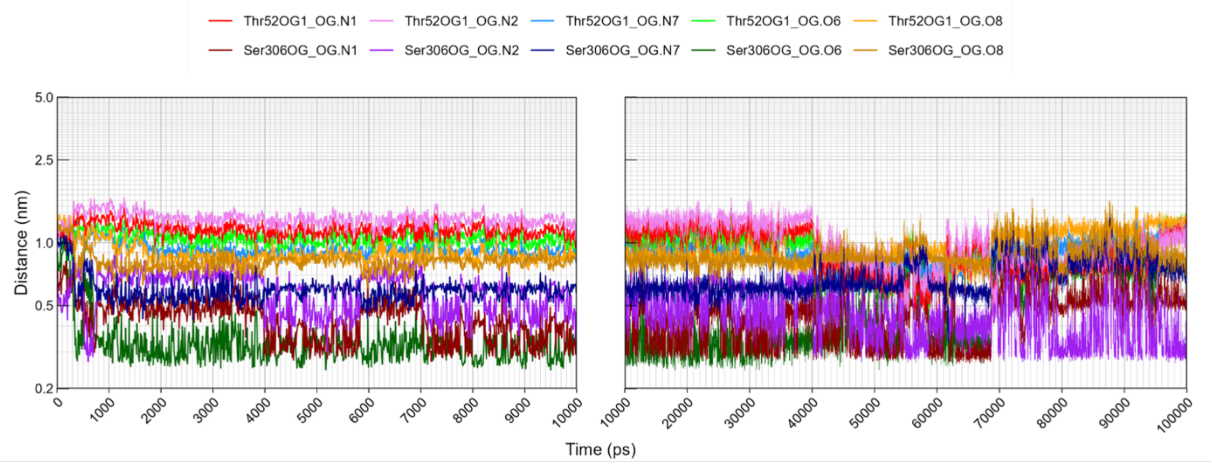

Energy

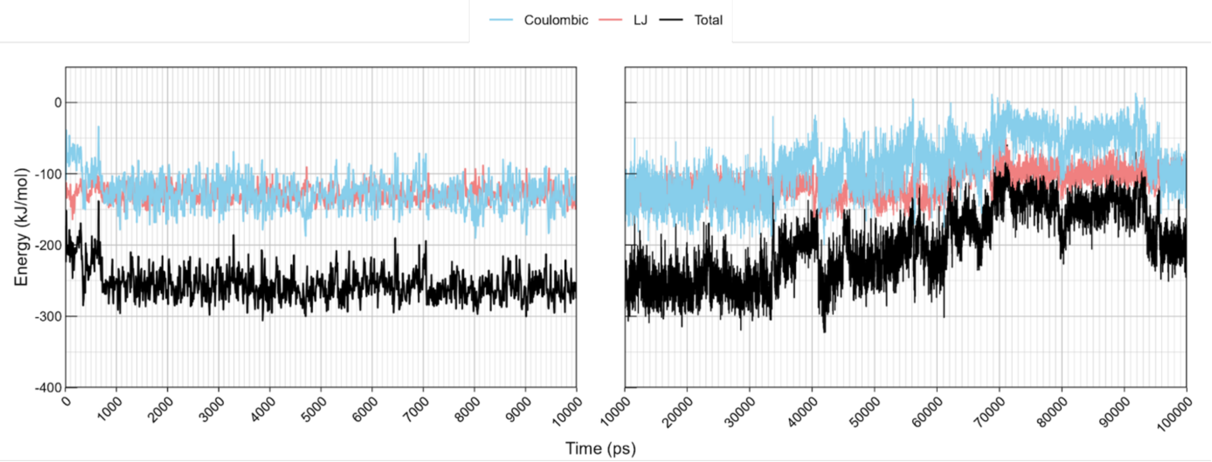

OG and Thiotrichaceae MutY 0,000 ps

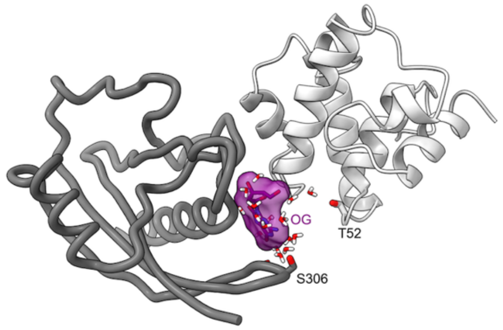

OG and Thiotrichaceae MutY 10,000 ps

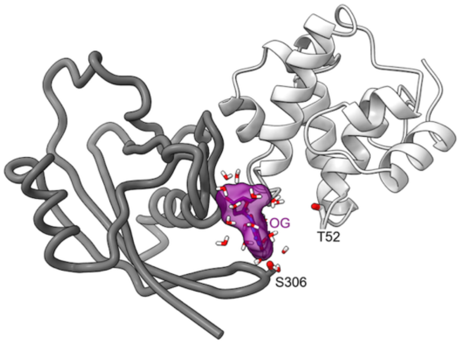

(I) *Flavobacteriaceae* MutY NTD complexed with adenosine

Distance Adenosine - Glu33

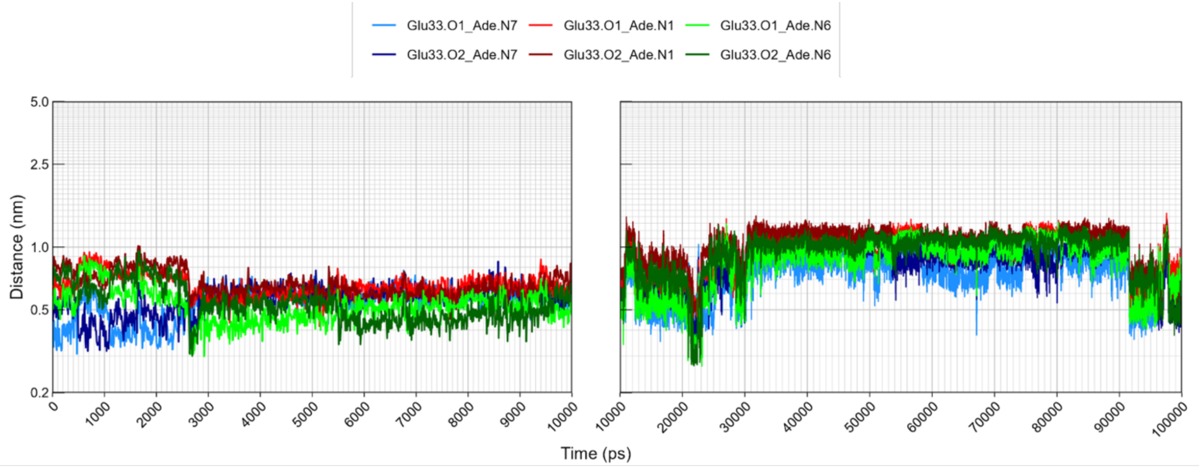

Energy

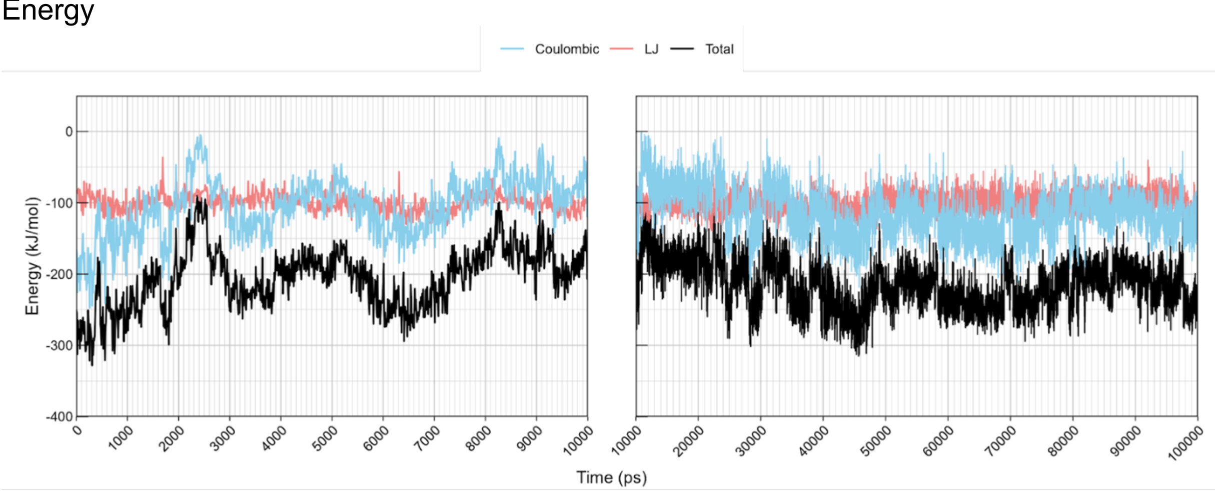

Adenosine and Flavobacteriaceae MutY NTD 0,000 ps	Adenosine and Flavobacteriaceae MutY NTD 10,000 ps
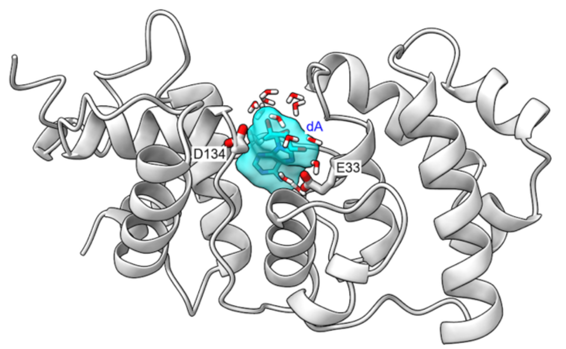	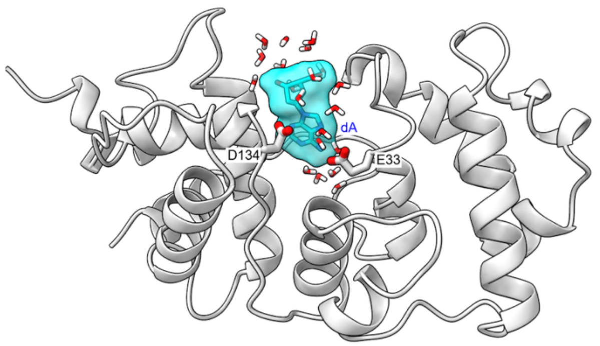

(J) *Flavobacteriaceae* MutY complexed with OG

Distance OG - Thr39 and Ser305

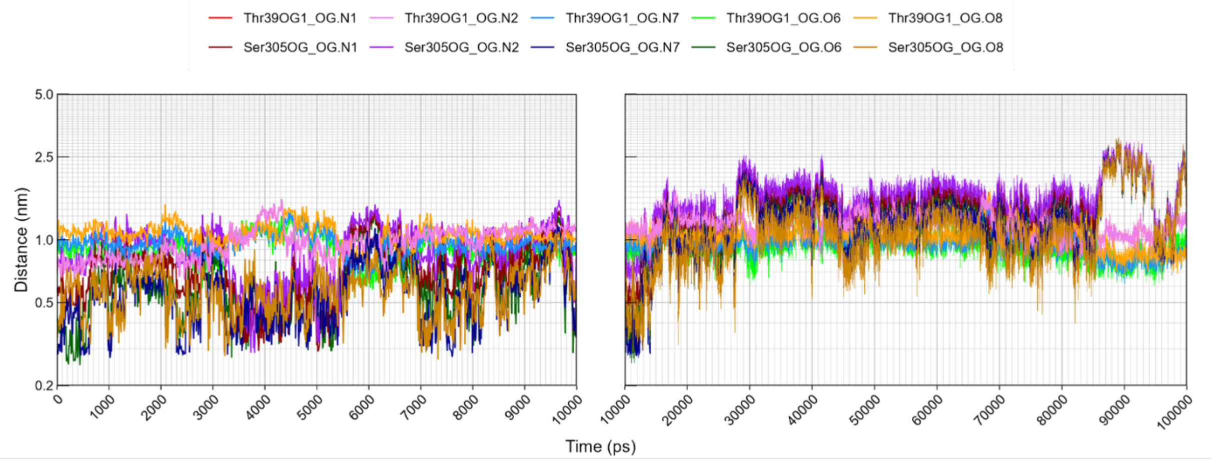

Energy

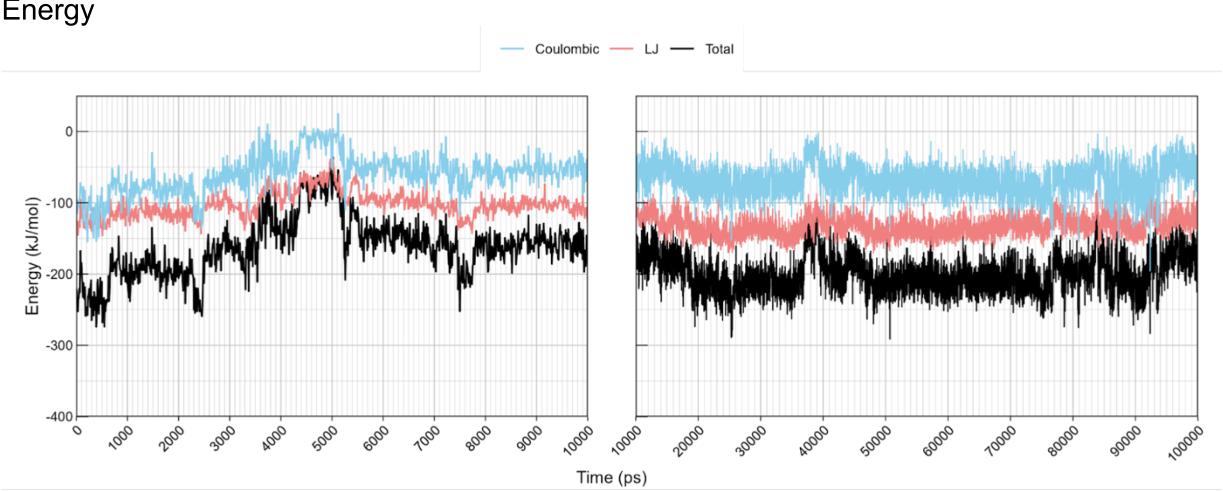

OG and Flavobacteriaceae MutY 0,000 ps

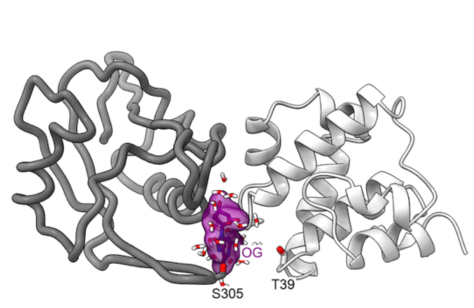

OG and Flavobacteriaceae MutY 10,000 ps

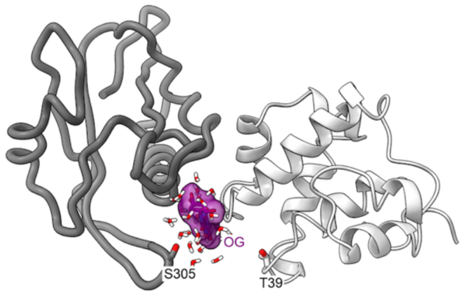

Supplement: S3 Fig — Molecular dynamics simulations were calculated by GROMACS with the Amber99SB and GAFF force fields for MutY complexed to adenosine and OG. For each MD simulation, short range interaction energies, distances between the ligand and functionally relevant residues, and representative structures sampled after equilibration and at 10,000 ps are shown. Note that the Y-axis is logarithmic for distance. The adenosine and OG ligands are shown with all atoms wrapped in transparent surfaces. For the adenosine complexes, the protein structure was truncated so as to focus on the NTD (residues 8–220 in Gs MutY, and corresponding residues for the LCHF MutYs). Catalytic residues are shown: Glu43 and Asp144 in the Gs MutY protein and corresponding residues in the LCHF MutYs. The distance versus time plot for the adenosine complex, tracks potential contacts between the catalytic Glu (atoms OE1 and OE2) and the hydrogen bond donors and acceptors on adenosine (atoms N1, N6 and N7). For the OG complex, the iron-sulfur cluster domain and inter-domain linker were omitted so as to focus on the OG-recognition site found at the interface between NTD (residues 29–137 in Gs MutY) and CTD (residues 234–360 in Gs MutY). Residues that interact with OG are shown: Thr49, and Ser308 in the Gs MutY protein and corresponding residues in the LCHF MutYs. The distance versus time plot for the OG complex tracks potential contacts between the critical Ser/Thr residues and the hydrogen bond donors and acceptors on OG (atoms N1, N2, O6, N7 and O8). The total short range interaction energy (black trace) is the sum of short range Leanord-Jones (salmon trace) and Coulombic (sky blue) interaction energies. (A) Molecular dynamic simulation for Gs MutY NTD complexed with adenosine. The ligand complex persisted for the entire 100,000 ps, with changes in location and orientation evident at 16,000 ps and 42,000 ps in the distance plot. Hydrogen bonds between catalytic Glu43 and the Hoogsten face of the adenine base we [file pone.0284642.s003.pdf]
