# Supplementary material for: Metagenome mining and functional analysis reveal oxidized guanine DNA repair at the Lost City Hydrothermal Field
Source: PLoS One. 2024 May 8;19(5):e0284642. doi: 10.1371/journal.pone.0284642 (PMC11078426; doi:10.1371/journal.pone.0284642)
Supplement: S1 Dataset — Chemical motifs are highlighted in columns. Alignment was generated by Promals3D [88], guided by the structure of Gs MutY. It was necessary to align sequences in the first block including up to N146 separately from the second block and third block because otherwise the C-terminal domain residues were aligned inconsistently. The homologs flagged with dark red highlighting were eliminated because of missing chemical motifs. The homolog flagged with light pink highlighting required manual adjustment so as to align the H-x-FSH motif. The representative LCHF MutYs have the following contig ids: Marinosulfonomonas MutY, c_000001803648; Rhodobacteraceae MutY, c_000002747260; Thiotrichaceae MutY, c_000000598175; Flavobacteriaceae MutY, c_000001535696. (PDF) [file pone.0284642.s004.pdf]

S1 Dataset. Alignment of Lost City MutY homologs.
Chemical motifs are highlighted in columns. Alignment was generated by *Promals3D* [88], guided by the structure of *Gs* MutY. It was necessary to align sequences in the first block including up to N146 separately from the second block and third block because otherwise the C-terminal domain residues were aligned inconsistently. The homologs flagged with dark red highlighting were eliminated because of missing chemical motifs. The homolog flagged with light pink highlighting required manual adjustment so as to align the H-x-FSH motif.
The representative LCHF MutYs have the following contig ids: *Marinosulfonomonas* MutY, c_000001803648; *Rhodobacteraceae* MutY, c_000002747260; *Thiotrichaceae* MutY, c_000000598175; *Flavobacteriaceae* MutY, c_000001535696.

```
Conservation:                    9 5 579 67999 5  5   65  7         55       6   9 98989 9   5       5        8        65969 67  66 5          859
sp_P17802_MUTY_ECOL      28  KTPYKVWLSEVMLQQTQVATVIP-YFERFMARFPTVTDLANAPLDEVLHLWTGLGYYARARNLHKAAQQVATLHGGKFPETFEEVAALPGVGRSTAGAILSLSLGKHFPILDGN  140
sp_P83847_MUTY_GEOS      34  RDPYKVWVSEVMLQQTRVETVIP-YFEQFIDRFPTLEALADADEDEVLKAWEGLGYYSRVRNLHAAVKEVKTRYGGKVPDDPDEFSRLKGVGPYTVGAVLSLAYGVPEPAVDGN  146
c_000003652391_2         37  PDPYTVWLSEVMLQQTTVAAVTG-YFRRFTTRWPTVQALAAADDGDVMGAWAGLGYYARARNLLKCARVVCETHAGAFPNTYAGLIALPGIGPYTAAAIAAIAFDRAEVVVDGN  149
c_000000031207_6         26  EDPYAIWLSEVMLQQTRVTTVIP-YWHRFLEKYPDPKSLARAPLEQVLELWAGLGYYSRGRNLHAAACQIVNDHDGIFPRDPELIGKLPGIGEYTTAAICSIAYGEPLAVIDGN  138
c_000000430975_4         38  PNVYHVWLSEIMLQQTKVATVLS-YYLNFLHKWPTLNDLATATRQNVLQAWAGLGYYARARNLHACAQIVMEKYGGTFPKTEKELLRLPGIGHYTAAAIVAIAHGQPSVVVDGN  150
c_000001007148_4         35  RDPYVVWVREIMLQQTTVTAVVP-YLDRFLKRFPTVESLAGAKSAEVLRVWEGLGYYSRARNLSRGAKLIVEEWGGQWRNTATELQSLPGVGPYTAGAIASFAFDQSAGIVEAN  147
c_000001092848_3         48  PDPYRVWLSEIMLQQTTVGAVKP-YCERFLERWPTVEALARADLQEVMKAWAGLGYYSRARNLKKCAEEVAHRHGGVFPDTEAGLKALPGIGDYTAAAVAAIAFGRRAAVVDGN  160
c_000001151029_1          1  ------WLSEIMLQQTKVATVLS-YYSNFLHKWPTLADLAMATRHNVLQAWAGLGYYARARNLHACSQIVMEKYGGTFPKTEKELLKLPGIGHYTAAAIVAIAHGQPSVVIDGN  107
c_000001797282_1         58  PDPYRVWLSEVMLQQTTIAAVRP-YFERFLTRFPDIFALAEAPEEAVMSAWAGLGYYSRARNLHACARTVAA-AGGRFPDTAEGLRKLPGIGAYTAGAIAAIAFDRQEAAVDGN  169
c_000001803648_25        40  PDPYRIWMSEVMLQQTTVAAVAK-YFHAFTTRWPTIIALASANDDDVMGQWAGLGYYARARNLLKCARVVVADYGGNFPDTRDELLKLPGIGPYTAAAIASIAFGRSETVMDAN  152
c_000002078955_4         40  AEPYRVWMSEVMLQQTTVAAVAK-YFHAFTKRWPTVDALAAAKDQDVMGQWAGLGYYARARNLLKCARMVVSDYNSEFPETRDELLKLPGIGPYTAAAIASIAFGRPECVMDGN  152
c_000002106160_4         28  KEPYKIWLSETMLQQTQVKTVIP-FYNSWIEKYPDFESVAGARLDSLLKSWEGLGYYNRCHNFHKAVKIIVNKYNSSLPINIKDFMVLPGVGSYTSAAVYSIVFLHPIPVVDGN  140
c_000003607531_2         38  KVPYKIWLSEIMLQQTTVTTVGP-YFQHFLKTWPTIDELANASLDDVLKVWQGLGYYSRARNLHKCAKLICNNFDGRFPNTEEQLEQLPGIGPYTAAAILAIAFDKPAIVVDGN  150
c_000003872363_4         36  PDPYHIWLSEVMLQQTTVAAVVK-YFNTFTKRWPNIQALATAQDADVMAEWAGLGYYARARNLLKCARMIVNDHGGVFPNDRAALLALPGIGPYTAAAISAIAFGRSACVVDGN  148
c_000004820107_1         43  PNPYYVWLSEIMLQQTTVVTVGP-YFTKFVERWPAIHDLAKADRDDVMHEWAGLGYYARARNLHKCAQIVSKELNGIFPEEQKDLEKLPGIGGYTSAAIRAIAFSKPANVVDGN  155
c_000005774797_1         29  PNPYHVLLSEFMLQQTTVATVKT-YFEQFTKKWPSVEHFSHATDDEIMAQWAGLGYYARARNLVKTIHTIH--NQGHFPASAYELQKLPGIGPYTSAAIAAIAFNEPILPRDGN  139
c_000005867021_3         40  PDPYRIWLSEVMLQQTTVVAVAK-YFHVFTKRWPNITALANAKDEDVMGEWAGLGYYARARNLLKCARMIVADFGGVFPDTRDDLLKLPGIGPYTAAAIASIAFGRSECVMDGN  152
c_000004546210_2         31  PSKYKTWISEIMLQQTQVNTVEP-YFHRFMERFPTVEELASSSPDEVLQIWSGLGYYARARNIHRSAQLIIKQHKGELPSDYESLLHLPGIGESTAGAILSLSGIEPKPILDGN  143
c_000004551008_5         31  PSKYKTWISEIMLQQTQVATVEP-YFIQFIQRFPEVTELASSSLDEVLQLWSGLGYYARARNIHRSARIIVNQHHSELPSDYESLLTLPGIGKSTAGAILSLSGIEPKPILDAN  143
c_000002511148_4         26  KTPYRVWVSEIMLQQTQVSTAIP-YFEKFIKEIPDVKSLSEASLDLVLSLWSGLGYYARARNLHKSAKIINLEHGGKIPSTRDELMALPGIGRSTAGAILALGFNKKAPILDGN  138
c_000000577378_2         14  KTPYRVWVSEIMLQQTQVSTVIP-YFKKFVREIPNIKSLSEASLDLVLSLWSGLGYYARARNLHKSAKIINLDHGGKIPSTGGELMALPGIGRSTAGAILALGFNMKAPILDGN  126
c_000000598175_2         37  INPYRVWVSEIMLQQTQVKTVIP-YFEKFMATFPDVQTLAHADQDLVLKHWSGLGYYARARNMHKAAKMICGDFKGQFPDNLEAMQSLSGIGRSTAAAILSIASNQPQAILDGN  149
c_000000754627_3         34  KTPYKVWVSEVMLQQTQVVTVIP-YFEKFMQSFPDIIALANADEDQVLHHWTGLGYYARARNLHKAAKIVRDKYQGQFPSTLEEVMDLPGIGRSTAGAVLSLSLGQHHPILDGN  146
c_000000811118_1         25  KDPYKVWISEIMLQQTQVTTVIP-YFNKFISEYPTIKTLASTSLDEVLSLWSGLGYYTRARNIHKTAKILKKDFDCKLPNEIEALMSLPGIGFSTAGAILSLGFEQSGVILDGN  137
c_000000899687_2         29  NDPYSIWVSEIMLQQTQVKTVIP-YYERWIKTLPTIDKLANAPEQKILKLWEGLGYYSRAKNLKKSAKIICKEMNGKLPNTVKNLQNLPGIGRYTAGAISSIAFGLKAPVLDGN  141
c_000001176522_24        50  RTPYRVWVAEVMLQQTQVKTVVP-YFERFMEAFPAVDVLADAEMDAVLSQWAGLGYYARGRNLHKAARQIVAQ--GGFPETLAGWQALPGVGRSTAAAIMAQAYGQRAAILDGN  160
c_000001345122_1          4  ISPYRVWVSEIMLQQTQVKTVIP-YYQRFMKSFPSIEQLASATQDEVLAHWAGLGYYARGRNLHKSAQIIQTEYYGKFPQIFDDIIALPGIGKSTAGAIMSIALKQRMPILDGN  116
c_000001515736_6         18  RDPYRVWLSEIMLQQTQVSTVLG-YYQRFLDAFPDVASLAGAPQDAVLALWSGLGYYSRARNLHKCAQTVMQQWGGAFPQTAEELATLPGIGRSTAGAISSFCFSERVPILDAN  130
c_000001682161_6         25  PTPYRTWVSEIMLQQTQVKTVIP-YYLRFMESFPTIQSLAKASQEQVLAHWAGLGYYARGRNLHKTAQTIVEQHKGQFPNRFEEVVALTGIGRSTAGAILSIAMQQRMPILDGN  137
c_000001923643_29        28  RDPYRVWLSEIMLQQTQVSTVLG-YYQRFLDAFPDVASLAAAPQDAVLALWSGLGYYSRARNLHRCAQAVVQEWGGAFPRRAEDLATLPGIGRSTAGAIASFCFSERVPILDAN  140
c_000002030994_5         26  INPYRVWVSEIMLQQTQVKTVIP-YYEKFMTSFPDVQTLASADQDTVLKHWSGLGYYARARNMHKAAQMVCDDFAGVFPDNLESMQSLSGIGRSTAAAILSIASNQQEAILDGN  138
c_000002038721_4         28  KSPYHIWISEIMLQQTQVKTVIP-YFHKFINSFPSIKSLAKSDLDKVLANWSGLGYYTRAKNIHQASKIIHQKYHGSIPKNYEQLIELPGIGESTAGALLTLAFDKPGIILDGN  140
c_000002523527_15        43  RDPYRIWLSEIMLQQTQVAAVIE-YFQRFVAALPTVAALAAASADEVMALWAGLGYYSRARNLHRCAKTVVDEHAGVFPTDPEVLVTLPGIGRSTAAAIAAFSAGVRSPILDGN  155
c_000002608528_2         26  INPYRVWVSEIMLQQTQVKTVIP-YYQKFMTTFPDVQTLANADQDLVLKHWSGLGYYARARNMHKAAKMVCDDFSGILPDNLEDMQSLSGIGRSTAAAILSIASNQSQAILDGN  138
c_000002961510_2         29  KDPYSIWVSEIMLQQTQVKTVIP-YYERWIKTLPTIGKLANAPEQKILKLWEGLGYYSRAKNLKKSAQIICKEMNGELPKTVNDLKNLPGVGRYTAGAISSIAFGLKAPVLDGN  141
c_000002979152_8         34  RTSYRVWVSEIMLQQTQVVTVIP-YYERFMQSFPAVEGLAKATQNEVLDHWTGLGYYSRARNLHKAAQFVVEQCNGVLPESLDGLIELPGIGRSTAAAILSLAGNKSEAILDGN  146
c_000003248034_1         46  KNPYHIWLSEVMLQQTRVTTVVP-YYERFLDRFPTLESLATASEQSVLQAWSGLGYYRRAKYLRASAQQIINKWNGQLPKEYDKLRALPGFGAYTAAAVSSIAFGRKHAAVDGN  158
c_000003294679_3         31  KTPYSIWVSEIMLQQTQVKTVIP-YFKKFIVSFPTLRVLAQASLDDVLHHWSGLGYYARAHNLHKTACLVNEKFSAQLPLDMKELCLLPGIGRSTAGAILALSCNQKHPILDGN  143
c_000003333364_1         28  YDPYEVWISEVILQQTQMERGVA-YIERFLERFPTMESLAAAEEDEVLKVWEGLGYYARARNLRKGAQHMVEEHGGTFPADVEAARALPGVGRYTAGAVLSQAYNLEEPIVDAN  140
c_000003402697_5         28  REPYYVWVSEIMSQQTQIDRVADQFFPRFIETFPTVEALAAADWETVYPCWDGLGYYNRGKNLLKAAKVLVDKHEGELPQTVAGLEALPGVGKYTAHAVLAFAFDKKVPAIDTN  141
c_000003492925_4         25  DDPYHIYLSEVMLQQTQVKTVLERYYFPFLKKFPTLKALGDANLDDVLKMWEGLGYYSRAKNLHKTAGLIEV-----LPSDIDELIRLPGIGKNTAHAVATFVYKKPVPIMEAN  133
c_000003766664_5         41  PTPYKVWVSEIMLQQTRVETVIP-YFQRFLSSFPDVHALAAASLDEVLSRWSGLGYYSRARNLHKASQFVVESWDGIFPSDAKMLEEMPGVGRYTAGAIASIAFGMPAPIVDGN  153
c_000003948186_72        28  ITPYRVWLSEIMLQQTQVATVIS-YFERFVQTFPDVGALARAPLDEVLHLWTGLGYYARARNLHKAAGLIVDTHGSELPDSVQQLCELPGIGRSTAGAICAIALRKHAVIMDGN  140
c_000005057120_4         28  ITPYRIWLSEVMLQQTQVATVIP-YFNQFIDYFPDIHKLATAEDDAVLHLWSGLGYYARARNLHKTAKILSKN-KGIFPDDLDSLMELPGIGRSTAGAILSIAFNNSYPILDGN  139
c_000005590109_5         28  ISPYRVWLSEVMLQQTQVTTVIP-YFNHFIQKFPDVHQLANAPLDSVLHLWSGLGYYARARNLHKTAKIISET-DGEFPNDLNLLMQLPGIGRSTAGAISSIAFNKSQPILDGN  139
c_000006063368_2         26  KSPYKVWISEIMLQQTQVTTVIP-YFNKFLLRFPNIEKLAESELDEVMSYWSGLGYYSRARNLYKAALEITCKHKSKLPNSYNELVALPGIGKTTAGAILALGFQKKAAILDGN  138
c_000000467631_2          7  STPYQTWLSEIMLQQTQVTTVIP-YFNRFIQTFPNIATLADAPLDAVLQHWAGLGYYARARNLHKTA-IIINKNGGVFPDDVDGLLELPGIGRSTAGAILSIAFNKSHPILDGN  118
c_000000788235_1         27  ITPYRVWISEIMLQQTQVATVIP-YYQRFITSFPDVTTLANADEDLVLQHWAGLGYYSRARNLHKAAQQVRDEFSATVPADLDSLQSLVGIGRSTAGAILSLAYEQRHAILDGN  139
c_000001187176_11        28  MTPYRVWVSEIMLQQTQVATVIP-YYERFMAALPDVHALAAAPADEVLHLWTGLGYYSRARNLHRAAKRVVEECAGQFPGDVQGLTDLPGIGPSTAGAIASLSMGVRAPILDGN  140
c_000001660697_64        28  GDAYRTWLSEIMLQQTQVSAVLG-YYARFIERFPTVQALAAAPADDVMAAWAGLGYYTRARNLHRCAQIVVAEHGGIFPRDPEVLASLPGIGRSTAAAIAAFSYGVRAAILDGN  140
c_000003378864_2         39  RDPYAIWLSEIMLQQTQVETVLP-YYDRFLTAFPTIADLAAAPLGRVLKMWEGLGYYARCRNLHRAAQKIVSDHNGRFPATFEDVLALPGIGQSTAGAILTFAYDQRHPLLDGN  151
c_000003910742_3         28  PTPYRVWISEIMLQQTQVVTVIP-YYQNFMDSFPDVRTLAHAEEDLVLQHWAGLGYYSRARNLHKSAKLICDEFSGVIPDDIDQIQSLPGIGRSTAGAILSLAYQQQQAILDGN  140
c_000004116181_2          1  -----VWVSEIMLQQTQVATVVP-YFNRFMAQFPDVKTLAQTSLDAVLQHWAGLGYYARARNLHKTAKIIAQQ--GCFPNTLETLMQLPGIGQSTAGAIMSIAFQQSCPILDGN  106
c_000006223903_1         29  RNPYRIWISEIMLQQTQVTTVIP-YYERFMARFPHIVTLASAKLDTVLAEWTGLGYYARARHLHQAAGVIVARHGGVFPDQFHDVVALPGIGRSTAGAILAFSHGQRHAILDGN  141
c_000001029068_2          2  -NVYYVWLSEVVLQQTQVASAVG-YFSNFIKRFPTIGALSAASEDDVLSAWSGLGYYARARNLHKTAQIINRQYSGVFPSTVEQVLALPGIGRSTAGAILSIAYNQACPILDGN  113
c_000001128125_11        36  ISPYRVWVSEIMLQQTQVATVIP-YFKRFMLSFPNVEVLAKATQDEVLSHWAGLGYYARGRNLHKAARQIVEQHDGIFPSQFDDILALPGIGRSTAGAILSISLNQRFAILDGN  148
c_000001595844_4         27  SDAYAIWLSEIILQQTRVAQGMP-YYIRFLEAFPTVFDLAKADEQEVMRLWQGLGYYSRARNLHFTAQQVANEFGGKFPDNYADLLALKGIGPYTAAAIASFAFGLPHAVVDGN  139
c_000001719155_2         31  RDPYRILVSEFMLQQTQVKTVIP-YFERWIKSFPTLQKLAEARESTVLKHWEGLGYYSRARNLRKAAQKIQQEYSGKVPESMNEIMKLPGVGRYTAGAVLSIAFGQKVPVLDGN  143
c_000002363038_6         36  INPYRVWVSEIMLQQTQVVTVIP-YFERFMQRFPEVSDLAAASQDEVLSHWAGLGYYARGRNLHKAAQLIVEQHNGVFPKSFDEVLNLPGIGRSTAGAILSISLQQRFPILDGN  148
c_000002561400_1          1  ---------EIMLQQTQVATVIP-YFERFMQRFPKVADLACADQEEVLTYWAGLGYYARGRNLHKAAQLIVEKHGGQFPSDFEEVLALPGIGRSTAGAILSISLEQRFPILDGN  104
c_000002566035_2         28  VSPYKVWVSEIMLQQTQVITVIP-YFKRFMKRFPDVQTLANSSLDEVLHYWTGLGYYARARNLYKTAKIITKEYQGNFPLSLNEIMSLPGIGRSTAGAVLSLSYDKCFPILDGN  140
c_000002598045_3         26  ITPYKVWVSEIMLQQTQVVTAIP-YFKKFIQEFPTVKSLSKATPDQVLKLWSGLGYYARARNLHKAAKLICSNHEGKIPSSINELVELPGIGKSTAGAILSLGFKKKAPILDGN  138
c_000004188575_41        28  PTPYQVWVSEIMLQQTQVATVIP-YYQRFMIRFPDNATLAAASQDDVLSYWTGLGYYARARNLHRCAQYVAEIHQGQLPAELDALIALPGIGRSTAGAIMALAYHQRFSILDGN  140
c_000000240049_1         26  KSLYRVWVSEIMLQQTQVTTVIP-YFARFMLQFNDIEALSNATQDDVLLYWAGLGYYSRARNLHKAAQIIQQQHGGCFPRQYNDVLALPGIGPSTAGAILAQSQGQRHAILDGN  138
c_000002139456_2         26  KSLYRVWVSEVMLQQTQVATVIP-YFARFMQQFSDIEALSNATQDEVLLYWAGLGYYSRARNLHKAAQIIQQQHGGCFPQQYDDVLALPGIGPSTAGAILAQALGQRHAILDGN  138
c_000003535614_2         28  RSLYRVWVSEIMLQQTQVATVIP-YYQRFMQRFKNIAALAEATQDDVLLHWAGLGYYSRARNLHKAAQIITASHQGKFPENYHDVLTLPGIGPSTAAAILAQATGQRHAILDGN  140
c_000002657784_4         25  KTPYKVWISEIMLQQTQVVTVIP-FYSAFINRFPNIQELATSSEEEVMSFWSGLGYYSRARNIYKTAQILQKKFESKLPSSLSQLISLPGIGPSTAGAILSLGYKKRAPILDAN  137
c_000003800129_15        30  TTPYRVWISEMMLQQTQVNTAKD-YFLNFIEQYPDLESIKNASEEEILILWKGLGYYRRAKFIYQAKEKIFKDYNGLFPDEFEDIISLPGIGKSTAGAILSIAYGKPFPILDAN  142
c_000003716781_1         17  DDAYSVWISESMLQQTRVETVIP-YFERFLKRFPTVEALAEADIDEVLARWSGLGYYSRARRLHAAAREIQDRHDGEFPQTRDEAEDLPGVGSYTAGAVLSIAYGLDEPLVDGN  129
c_000001556689_3         18  RDPYAILVSEIMLQQTRVETVIN-RYGSFLERFPTIADLAIASLDDVLAEWSGLGYYRRPRLLHRLATVVVDTHGGEIPDTLEELLELPGIGPYTSAAVGSIAFGIPALSIDGN  130
c_000001834452_1          1  ------WISEIMLQQTRVEVVVP-YFEAFIKRFPSIEVLANAQEEEVLSYWAGLGYYKRSRLLLKAARVIQSEMDGVLPCDSATLRRLPGIGDYTAAAIASISFQEQIPVVDGN  107
c_000004255004_2         35  RDPYAIWVSETMLQQTRVATVLP-YYYRFMKAFPSVEALDQAPLDAVRNVWSGLGYYQRAANLKKAASVLMNRHNGKLPADYQTLLGLPGVGQYTAGAVLSIAFDRRYPAPDGN  147
c_000000605438_3         31  QSAYEIYLSEIMLQQTQVKTVLERFYFQFLEKFPTLKAVANAPVDDILKAWEGLGYYTRARNLHKTAIVTKGI----LPKSAEALEQLSGIGKSTAHAVACFAFNEPLPILDAN  140
c_000000581237_15        31  QSAYEIYLSEIMLQQTQVKTVLERFYFQFLDKFPTLKDVANAPVDDVLKAWEGLGYYTRARNLHKTAVDTKGI----LPNNAEKLEELSGIGKSTAHAVACFAFDEPLPILDAN  140
c_000002529579_2         31  QNSYKIYLSEIMLQQTQVKTVLERFYFQFLEKFPTLEAVANAPVDDVLKAWEGLGYYTRARNLHKTAIATKGV----LPKRAEALEKLSGIGKSTAHAVACFAFNEPLPILDAN  140
c_000006097838_1          1  RDPYAIWISEVMLQQTQVKTVIP-YWRRWMEQLPDIASLAAADEDTVIKLWEGLGYYSRARNLQKAARLLCEELSGKFPRDLCGVLALPGVGRYTAGAICSIAYNQPEPLVDGN  113
c_000000583727_6         27  RDPYAIWVSEIMLQQTQVKTVIP-YWQRWMKQLPDIASLAAADEDTVIKLWEGLGYYSRGRNLQKAAKRIYDELAGRFPRDLAGMLALPGVGRYTAGAICSIAYNQPEPLVDGN  139
c_000005807640_2         29  RDPYAIWVSEIMLQQTQVKTVIP-YWQRWMQRLPDIASLAAADEDTVIKLWEGLGYYSRARNLQKAAKRIRDALSDRFPRDLAGALALPGVGRYTGGAICSIAYNQPEPIVDGN  141
c_000000134878_1         12  NDPYKIWISEIMLQQTQVSTVQD-YYLRWIKRFPTIQSVADASIDEVLKHWEGLGYYARARNFYHSCKALSTTT-SCVPKGMDEFQKLKGVGPYIGAAVQSIAFNIPAGVVDGN  123
c_000005136725_3         27  SDPYKIWISEIMLQQTQVSTVRD-YYIRWIKQFPDIQSVADASIDKVLKYWEGLGYYSRVRNFHKSCQMLIRNK-QDIPKGLDEFQKLKGVGPYIASAVQSIAFNIPTGVIDGN  138
c_000001684786_4         24  PEPYRVWVSEIMLQQTRVETVIP-YFERFLDRFPTPAALAEASEDEVLTLWSGLGYYRRARSLLEGAKAVVRDHEGVFPRSYEAALDIPGVGPYTAAAILSIAYGEPWPVLDGN  136
c_000002971826_23        34  PEPYRVWVSEIMLQQTRVDTVIP-YFERFLDRFPTLSDLAAASEDEVLTLWSGLGYYRRARSLLAGAKTVIEHHAGVFPRDYEAALEIPGVGPYTAAAILSVAYGEPWPVLDGN  146
c_000002040695_2         37  ANPYFVFISEVMLQQTTVATVVP-YFKKWIKKWPTLHALAEASLDDVLHTWQGLGYYRRARNVHRCAQEVVQSYGGLFPQNVSQLRKLPGIGPYTAGALSAIAFEQNSVAVDGN  149
c_000003263657_32        41  NDPYSVWISEMMLQQTQVTQAEP-YYERFMAQFPSVETLAGATLDDVLKAWEGLGYYARARYLHRAAVQVVDQYGGRIPESLSEISILPGIGPYTAAAILSIAYHRDHAAVDGN  153
c_000005989041_2         38  KDPYKIWLSEIMLQQTTVKAVGP-YFQLFLKTWPTIDKLAKATLDDVLRVWQGLGYYSRARNMHKCAKLICNNFGGRFPNTEEQLERLPGIGPYTAAAISAIAFDKPANVVDGN  150
c_000002391082_2         36  DNPYHIWISEIMLQQTRVDQMGA-YFERFVGAFPTVEKLAAASEDQVLKVWEGLGYYARARNMHKAAVQIAGQLGGCIPDTYAGLIELPGIGEYTAAAVSSIAFDRDHPVLDGN  148
c_000003839553_2         43  SDPYHIWISEIMLQQTRVDQMGG-YFERFITAFPTLEALAGASGDEVLKVWEGLGYYARACNMHKAAKRIAFELEGQIPNTYDGLIDLPGVGEYTAAAVSSIAFDCDHPVLDGN  155
c_000003996707_2         34  GNPYHVWISEIMLQQTRVDQMQP-FFERFTAAFPTVGALAAASQEEVLKAWEGLGYYARARNLHRAAQRVV-EAGGNIPDRYEELVGLPGIGPYTAAAVSSIAFDRDHPVLDGN  145
c_000002044706_1         16  DDPYRILVSEIMLQQTRVTTVLR-YYPAFLERFPTLADLAAADQEDVLAAWSGLGFYRRARNLHACARQVVDRYDGEVPRDPEELGSLPGIGRYTVGAILSSAHNARLPILDGN  128
c_000006007075_1         31  KREYYTLVSEFMLQQTQVSTVIP-YFKNFIKDIPDLNSLAKINEKKLLKYWEGLGYYSRVRNLKKTAKIVVSKYKKKLPTTLEELKTLPGIGDYTASAILSIAFNKPFIPLDGN  143
c_000002094036_4          7  QKEYYTLVSEFMLQQTKVSTVIP-YFNNFLKNIPNMQSLAKINEKKLLKYWQGLGYYSRAKNLKKTAKIIVDNYQGKLPSDFEELKKLPGIGDYTANAISAIVFNKPTIPLDGN  119
c_000002762689_1         31  KKQYYTLVSEFMLQQTQVATVIP-FFNRFIKNIPDLEALANFDNHKLIKLWEGLGYYSRVRNLKKTAQIVVKNFDKKLPRKYLDLKSLPGIGDYTASAISAIAFNKRIIPLDGN  143
c_000005516980_2         31  KREYYTLVSEFMLQQTQVSTVIP-YFKNFIKNIPNLKMLAKVQNKKLIKLWEGLGYYSRARNLKKTSQVIIKNFKGKVPENFEDLKSLPGIGNYTASAILAIAFNKPYIPLDGN  143
c_000003920004_1         37  PNPYYVLVSEFMLQQTTVNTVIS-RFKDFIKKWPNLKKLSMINENQILQFWSGLGYYARAKNLLNSAKIISLKFNNIVPDNYNDLIDLPGVGDYTAKAVLGIGYNKSVMPVDAN  149
c_000004008511_2         31  PDPYFVFVSEYMLQQTTVGTVKT-RFEEFILKWPSINDLARISEKTILNFWSGLGYYSRARNLLKAAKIIKKEFNSKIPNNYDDLINLPGIGDYTTKAILGIAYNKSVMPLDVN  143
c_000004481347_1         38  TNAYYVLVSEFMLQQTTVNTVTK-RFNEFIKLWPSIDKLSRISENRILRFWSGLGYYNRATNLLKTIKIISRDFKFKVPQHYDQLIELPGVGDYTAKAILGIAYNQPVLPLDAN  150
c_000003787733_3         30  NDLYKIWLSEVMLQQTRVNTVVP-YYLLWVKKYKSIKSVAKADYQNLLKLWEGLGYYSRCKNFYKSCLIIINDYDGKIPLDYLTFRSLPGVGDYIASAVLSIGLNQPHAAIDSN  142
c_000000754657_2         30  HDLYKIWISEVMLQQTRVNTVVP-YYLRWVEKYESIKSVAKADELKLLKLWEGLGYYSRCKNFHKACKIIVNDYDGEVPSDYQKFLLLPGVGEYTASAVFSIGLKQKYAAIDGN  142
c_000000990943_3         30  NDLYKIWISEVMLQQTRVNTVVP-YYLRWVEKYKSIKSVAKADDLKLLKLWEGLGYYSRCRNFHKACKIIVNDYGGEFPSDYQKFRLLPGVGDYTASAVFSIGLKQTYAAIDGN  142
c_000004474996_2         29  NDLYKIWLSEVMLQQTRVETVIP-YYLKWVEKYKTIKSVAEADGQKLLKLWEGLGYYSRCRNFHKACKIVVNDFTGEIPSDYQNFRSLPGVGDYTASAVFSIGLNQTYPALDGN  141
c_000001463500_11        17  NNLYHVYLSEVMLQQTKVKTAIP-YYKRWLKKYPTIESVALSNLDDLLKLWEGLGYYSRCVNFYKACIILYSQYNSQLPNNMTDFLKLPGVGNYISAAVYSIVDKIPAPAVDVN  129
c_000001286181_5         24  NDPYKVWVSEIMLQQTQVSTVIP-FYENFMKSFPDVLTLSKANLDEVLNNWAGLGFYRRAKNLHQTAKIITDKYHQVFPNNYEDLVALPGIGESTAGAILALAFNKKGTILDGN  136
c_000001293628_3         30  REPYRVWVSEIMLQQTRVETAIP-YYNRWMERFPTVDALAEGHEQAVLKAWEGLGYYSRARNLRRSALIVRERLGGVLPQDSRSLKTLPGIGEYSAGAIASIAYGEVVPAVDGN  142
c_000001535696_8         24  RDPYKIWLSEIMLQQTRVAQGAP-YYLKFIKHFATVYDLAKASEEQVLKLWQGLGYYSRARHLHATAKTIVNEYGGNFPNTYKELLKLKGIGDYTASAIASICFDRPEPVVDGN  136
c_000001614067_2         37  SNPYFVYLSEIMLQQTVVKTVIP-YFLKFVRKWPDINALAKAELHEINSYWAGLGYYSRAKNLHETAKIISNKYDGFLPTDKNSLMALPGIGEYTSSAIMAIAFDRKSNVVDGN  149
c_000001765289_1          3  DDPYKIWLSEIMLQQTQVKTVVP-YYNRWIKRYPSIKSVALADLGAVLKMWEGLGYYTRCRNFHTAAKIVVKKFNGIIPNNWENFSSLPGVGDYTAAAVLSIAFNKPYAVMDGN  115
c_000001961666_1         18  VDAYKVWVSEIMLQQTTVNAVIS-RYKKFISRFTNVKVLAKAPLEEVLEEWAGLGYYSRARNLHSSAKIIVNKYEGIIPSEENKLINLPGIGTYTSGAIRAIAFNRPSLAIDVN  130
c_000002018097_4         25  NEPYNIWLSEVMLQQTQVKTVVP-YYTKWIKKFPTLKSVAESDLNSLLKLWEGLGYYSRCRNFHKATQIVVEKYKSVVPNDYKLFRQLPGVGDYIASAVLSIAYNKKYPAVDAN  137
c_000002843512_36        26  DDPYHIYLSEVMLQQTQLKTVLERYYFPFLKRFPTLKTLGETPLDDVLKMWEGLGYYNRAKNLHKTATLIEE-----LPSNIDELIKLPGIGKNTAHAVATFAYKKPVPIMEAN  134
c_000002930199_1         22  NNPYKIWISEIILQQTQIQTGIR-YYHKFIRHFPDINSLALAEEVDVLNVWQGLGYYNRVLNMLYSAKIIVKQHKSTFPTKYDELIKLKGIGPYTAAAISSICKNEKRAVLDGN  134
c_000003033795_2         22  RTPYRVWVSEIMLQQTQVNTVIP-YYKKWIKKYPTLKSFKESNFDDVIKIWEGLGYYSRCHNMFNAAKLI----NSTFPNNYDDLINLPGIGDYTAKTILAIAFKKNFVGIDTN  130
c_000003888107_2         23  HDPYKIWISEIMLQQTQVSTVIP-YYNKWINKFPNATKLSKANYNDVLKLWEGLGYYSRCKNIYKTSHLV----QKNFPDSYSELIKLPGIGDYTAKTILAIAFDKNQVGIDTN  131
c_000004013286_6         44  RDPYAIWVSEIMLQQTQVNTVVP-FFERFIESFPTVEKLAAATEAAVLKRWEGLGYYRRARNLHRAAREVTDQYDGQLPRDPATLSRLPGIGQYTAGAILSIAFDCREPIVEAN  156
```

```
c_000004852258_1         6  SDPYKIWLSEVMLQQTQVETVIP-YYQKWINHYPTIKSVALENIDNLLKIWEGLGYYSRCRNFHEAIKDIFYNHNGVIPKQIEDFKSLKGVGIYISGAVMSIAFNQTHIAVDGN  118
c_000005254087_1        32  RDPYAVWVAEVMLQQTQIATVIP-YYERWLARFPTVQALAEASLDEVLKLWEGLGYYGRARNLHAAVQMVMNEYDGRIPTTAADLLKLKGIGRYTANAIASIAYNEAVPVLDGN  144
c_000005603677_1        29  QDPYKIWLAEIIMQQTRLEQGLP-YYERFIAAYPSIKELAETEEQKLMKLWQGLGYYARARNLQATAKRIVAEYAGIFPDNYKAILDLKGVGEYTAAAIASFAYDLPYAVVDGN  141
c_000003283462_3        17  TGFYRIWLSEIMLQQTQVKRVSI-FYTKWIKRFPTLESVANAKEEHLLKVWEGLGYYNRCRNFYKATKIIIDDYNGKIPNDYQTFRLLPGVGDYTASAVLSIAFGMPYPAIDGN  129
c_000002687221_1        31  SNPYFIWLSEVMLQQTRVSTASP-YYQHWISALPDIHSVAEAPIDNILKLWEGLGYYGRARNFHKACRIIIEKHGGVIPSDPLEFSKLPGVGPYICAAVMSIAFNLPIPAADGN  143
c_000003347358_19       57  DEAYYIYLSEVMLQQTQVKTVLERYYIPFIERFPTLKHLGEAPLDDVLKMWEGLGYYNRAKNLHKTATLVDQ-----LPSNIDELIKLPGIGKNTAHAVATFAFHQPVPIMEAN  165
c_000002701031_2        24  SDSYKIWLSEIILQQTRVEQGLP-YFKEFITKFPKIELLAKASEDEVLKLWQGLGYYSRARNMRLTAKAIVEKFNGVFPKDYEKILKLKGIGPYTAAAICSFAYNLPYAVVDGN  136
c_000000582753_3        32  KSSYKIWLSEILLQQTKVSQGLP-YYRSFIESYPRLEDLAHSSEEKVLKLWQGLGYYSRARNLHFTAQYIFNELSNEFPNNYKDLLKLKGVGDYTASAIASFAFDEVVPVLDGN  144
c_000001713769_5        24  NDPYSVWLSEIILQQTRIAQGLP-YYLKFINKYPTINSLAKANENDILILWQGLGYYSRARNLLKTAKFIVDERNGKFPTTYIELIKLKGIGEYTAGAISSICFNERRAVLDGN  136
c_000004369364_1        27  SDPYAIWLSEIILQQTRVAQGLP-YYLKFLEAFPTVQDLAQAPEQDIMLLWQGLGYYSRARNLHFTAKQVSDELNGLFPNSYTGLLSLKGVGPYTAAAIASFAYKLPHAVVDGN  139
c_000006057486_30       28  KDPYLIWLSEIILQQTKIAQGTP-YYLKFVEKFGNVSLLAKADEKQVLKLWQGLGYYSRARNLHKTAIYINNNLKGVFPNNYTDLKKLKGIGDYTASAIASFCFNLPEATVDGN  140
c_000005494072_10       24  KDPYKIWLSEIILQQTKIEQGLP-YYMSFVENYPNIETLAIADEQEVLKLWQGLGYYSRARNLHFTAKSIVKNHNGKFPDNYNELLKLKGVGDYTASAISSICFDEKCAVVDGN  136
c_000001742634_3        27  KKPYFIWLSEIILQQTRTTQGLP-YYIAFIKHFPTIFDLAKTSETKVLKLWQGLGYYSRARNLHFTAKYIVDNYNGKFPENYKELIKLKGVGDYTASAIASICYNEKTAVVDGN  139
c_000002826998_2        27  KNPYLIWLSEIILQQTRVEQGLP-YFLKFKEKYPTVTDLANAEEDEIMKLWEGLGYYSRARNLHFSAKYITAELDGIFPKKHTEILKLKGVGTYTAAAISSFAYDLPHAVLDGN  139
c_000003159439_6        24  SNPYHIWISEIILQQTRVDQGLD-YYKRFIATFPTINNLAKAKEEAVLKLWQGLGYYSRARNLHFSAQYIVNELDGKFPSSYKELLKLKGVGDYTASAIASICYNEVTAVVDGN  136
c_000004887214_1        29  REPYKIWLSEIILQQTRVAQGLP-YYQRFIESFPILQNLAAASEQNVLKLWQGLGYYSRARNMHFTAQYVVNELGGEFPCSYKELMKLKGVGDYTASAVASFCYDEVQPVLDGN  141
c_000005037037_2        27  KNPYYIWLSEIILQQTRVEQGTK-YFLAFKKNYLTIFDLAQAKETEVLKLWEGLGYYSRARNLHFTAKYIANDLEGVFPRNYTDLLKLKGVGSYTASAIASFAYDIPKAVVDGN  139
c_000001059964_1        19  YEPYQVWISEIMLQQTQVQTMLP-YYHRWMERLPRLQDVAEASEDLLIKLWEGLGYYRRVHNIKKTAVIICRDYGGTFPGSYRQQIQLPGIGPYTAGAIASIAFNFSEPVLDGN  131
c_000002498472_1        28  YSPYQVWISEIMLQQTQMDRVTL-FFTRWMDTFPDVNTLASASETRVLKLWEGLGYYSRARNILKTAQLLVTEYQGQIPDDPKQLLSFPGIGPYTAGAIASISFNHDVPVVDAN  140
c_000004615912_3        17  KNPYLIWLSEVILQQTRTAQGLP-YYEKFKNRFPTIDLMANAHEDEIMKLWEGLGYYSRARNMHFTAKYITEELGGEFPDTLEGLKKLKGVGPYTAAAIASFAYDLPHAVLDGN  129
c_000004766858_2        30  YHPYEIWVSEIMLQQTQVKTMLP-FYTRWMSELPTVHSVAAASEEMVLKLWEGLGYYSRARNLQKAARVICDEHGGELPADFDHIRALPGIGPYTAGALASIAFNQPRPVVDGN  142
c_000002747260_18       36  PDAYAVWLSEVMLQQTTVAAVRD-YFRRFTTRWPTVADLAAAEDADVMGEWAGLGYYARARNLLKCARVVVAEHGGHFPDTRDGLLKLPGIGPYTAAAIAAIAFDEPAVVVDGN  148
c_000005371561_38       36  PDPYRVWLSEIMLQQTTVAAVRD-YFHRFTARWPTVADLAAAEDGDVMGEWAGLGYYARARNLLKCARVVVSEHGGRFPDDPDALLALPGVGPYTAAAIAAIAFDKPETVMDGN  148
c_000000169465_2        32  VTPYKVWVSEIMLQQTQVTTVIP-YFKRFMASFPTVHDLAKASQDDVLHHWTGLGYYARARNLHKAANTLVEKYNGEFPYSLEEVMDLPGIGRSTAGAILSLSRNMRFPILDGN  144
c_000003254110_11       32  VTPYKVWVSEIMLQQTQVTTVIP-YFERFMASFPTVHDLAKASQDDVLHHWTGLGYYARARNLHKAANRLVDEYNGEFPFSLEEVMDLPGIGRSTAGAILSLSRNMRFAILDGN  144
c_000004750284_20       32  VTPYKVWVSEIMLQQTQVATVIP-YFNRFMASYPSVFELANASQDDVLHHWTGLGYYARARNLHKAAKTLVEDYEGVFPPTLDEVVSLPGIGRSTAGAILSLSRDERYPILDGN  144
c_000000141782_15       37  ATPYRVWISEVMLQQTQVATVIP-YFERFLQRFPDVATLAAADADSVLHLWSGLGYYARARNLHRAARVVVEAHGGEFPRRFEEVQALPGIGRSTAGAILSLGCGQRHAILDGN  149
c_000002717847_8        31  ITPYKVWISEMMLQQTQVVTAQS-YYSKFLKKYPSLTSMKMATEDDILSLWSGLGYYRRASFIYQAKEKIFLDFNGRFPATYDELITLPGIGRSTAGAILSIAYQKPFPILDAN  143
c_000004054799_1        18  ISPYRVWISEIMLQQTQVKTVIP-YFYKFIKKYPNLGALLQASEDEILAQWSGLGFYRRAKNIYKACRIISEDFNSKLPTNINDLESLPGIGRSTAGAIMSIAFNEGHPILDAN  130
c_000006126673_1        43  PNPYFVWLSEIMLQQTTVSAVIP-YFLKFTQRWPDVWALANADVDDIMSAWAGLGYYARARNMHKCAKTIVSDYDGIFPQEQAVLKTLAGIGDYTSAAITAIVFDKYATVVDGN  155
c_000000339186_3         1  -DPYSIWVSEIMLQQTQVSTVIA-YWERWMRALPTIDKLARAKPTQVLKLWEGLGYYSRARNLQVAAKEIVKNHDGQFPRDRPAIRALPGIGRYTAGAISSIAFGLPEPIVDGN  112
c_000003029168_2        32  RDPYAIWISEIMLQQTTVESVIP-IYERFMELFPTVDVLAVANPTDVLQAWAGLGYYKRARHLHEAAIEIVNRFGSRIPDHLSDLQSLPGIGRYTAGAIASIAFDQCVPVVDTN  144
c_000001863436_1        48  GDPYAIWVSEVMLQQTQVVTVID-YYTRWLARFPDVETLAAAEIDEVLELWAGLGYYRRARLLHRAAQHVVEECGGALPQTAGELEDLPGIGPYTAGAIASIAFGEAAPLVDGN  160
c_000002830137_4        41  RDLYHIWVSETMLQQTQVATVVP-YFQRFVERFPNITSLAAADEEDVLRLWEGLGYYRRARNLHRAAQQVVQQHHAQFPADFDTLLSLPGIGRYTAGAILSIALDQRLPILEAN  153
c_000004612302_3        35  RDPYAVWIREIMLQQTTVKAVVP-YLERFLDQFPTIEVLAASETDEVLRVWEGLGYYSRARNLHRAARMIVEEWGGQWRETAVELQALPGVGPYTAGAIASFAFDQPAAIVEAN  147
c_000001933926_1        21  TDPYRVWLSEIMLQQTTVATVTP-YFLRFVERWPTVHDLAAADLDRVLHAWQGLGYYARARNMHACAKRISGDMAGVFPDQENELRRLPGIGPYTAAAIAAIAFDRPATPVDGN  133
c_000003136334_1        33  MDPYRVWLSEIMLQQTTTITVRG-YFEDFVSRWPSIEDLAAAELDAVLHAWQGLGYYARARNLHKCAKLITKEYGGKFPEREEALVKLPGIGPYTAAAIAAIAFGHIATPVDGN  145
c_000005849454_9        30  RDPYRTWVSEIMLQQTRVEAVIP-YFEAFLKRFPDVHALATATEEEVLQRWAGLGYYRRARFLHAAAQKVVEDWDGRFPETADTLRAMPGVGAYTSAAIASLAFGEAVPVVDGN  142
c_000001169194_1        41  TDPYRILVSEIMLQQTRVETVLR-YYESWLKRFPTLGALASANSREVLKAWEGLGYYRRARNLHSAARRIRELPGGHFPTSYRDLRKLPGVGQYTAGAVASIGFGEIVPAVDGN  153
c_000002786947_2        41  TDPYRILVSEIMLQQTRVETVLR-YYESWLKQFPTLGTLASADSTEVLKAWEGLGYYRRAKNLHRAAKHIRNVLGGRFPTSSRDLRQLPGVGQYTAGAVASIGFGEIVPAVDGN  153
c_000001279808_52       28  RSPYYVWISEIMSQQTQIDRVAEKFFPRFIATFPTVEALAAASWDEVYPVWEGLGYYNRGKNLLKAAQIIVEKHDGEIPQTAVELEALPGVGKYTAHAVLAFAFDQKVPAIDTN  141
c_000000358065_2        31  QKQYFILISEFMLQQTQVKTVIP-YFENFINKIPNLKSLAKVNNRKLMKCWEGLGYYSRAKNLKKSAKIIIDNYKGRLPNDFEELKKLPGIGDYTASAISAIAFNKPIIPLDGN  143
c_000003745941_1        22  AALYPTVVSEFMLQQTQVKTVLP-YFHRWLKRFPDFKSLAKAKEETVLKHWEGLGYYSRARNLHRLAREILALN--SIPEDPESWQQFKGIGAYTAAAITSISFNHPVACVDGN  132
c_000004187032_4        31  KKHYYTLVSEFMLQQTQVVTVIP-YFDRFINKIPNLKKLSTIQDNELIKLWEGLGYYSRARNLKKTAQIIISKHHGKIPNNYEDLISLPGIGNYTANAILAIAFNKSYIPLDGN  143
c_000006067315_3        31  QTEYFTLVSEFMLQQTQVKTVIP-YFNNFINKIPNLKKLANVNETRLMKCWEGLGYYSRARNLKKTAKIIISEFNSNLPNTIEELKNLPGIGDYTSRAVLAIAFNKPIIPLDGN  143
c_000005543774_1         1  KKLYYRILSEFMLQQTQVKTVIP-YFNNFVKKVPSLKALSKSNEKKILKLWEGLGYYSRAKNLHKTSKILVKKYDSQIPDSFEKLKELPGIGDYTANILLALIYNHPRIGIDGN  113
c_000006211484_1        23  KKQYYRILSEFMLQQTQVKTVIP-YFNNFVKKIPSLKALSNSDEKTVLKLWEGLGYYARAKNLHKTSKILIKKYNGQIPKSFVKLKELPGIGDYTANILLALIYNQPCIALDVN  135
c_000002718976_3        32  KKLYYRLLSEFMLQQTQVKTVIP-YFNKFTKKFKTIKALSKSNEREILKMWEGLGYYRRARNLLACSKILVKNYKSRLPRSIIEIKKLPGIGDYTANALLGLVYNEPRIAVDGN  144
c_000002992548_1        33  KEQYYRILSEFMLQQTQVKTVIP-YFKNFINKIPNLKVLSSSNEKTILKLWEGLGYYRRAKNLHKTAKILTKNYKGKLPKEFDEIKKLPGVGEYTANALLALVYNQPRIPFDGN  145
c_000000456751_1         1  ----YVLVSEFMLQQTQVKTVIP-YFTKFVKKIPNLKSLSTSNEKKILKLWEGLGYYRRARNLHKTAKILIKNCKGKLPKKFAEIIKLPGVGEYTANSLLALVHNKPSIPIDGN  109
c_000002655634_1        28  KKEYYVLVSEFMLQQTQVKTVIP-YFKKFVTKIPNLKTLSTSNEKQVLKLWEGLGYYRRVRNLHKTAKILLKQYKAKLPKKFQEIIKLPGVGEYTANSLSALIHNKPCIPIDGN  140
c_000004232403_2        27  NDPYKIWLSEVMLQQTRVETVIP-YYNTWLENYKTIQSVAQANLDDLLKIWEGMGYYSRCKNFYYAAKEIICKYKGTIPNQFDVFKSLPGVGDYIAGAVMSIAFNQPHPSIDGN  139
6u7t.before.V147.pdb    28  RDPYKVWVSEVMLQQTRVETVIP-YFEQFIDRFPTLEALADADEDEVLKAWEGLGYYSRVRNLHAAVKEVKTRYGGKVPDDPDEFSRLKGVGPYTVGAVLSLAYGVPEPAVDGN  140
Consensus_aa:               .ssY.l@lSElMLQQTpV.hhhs.Y@.p@hpp@PslpsLA.tpbppllphWpGLGYYsRt+Nlhphtp.l.pph.t.hPpshp.l..LPGlG..TAtAlhtht@sp..shlDtN
```

```
Conservation:                 95  9                                                       75 87 85   9    5 9      8     9 5
sp_P17802_MUTY_ECOL    141  VKRVLARCYAVSGWPG------KKEVENKLWSL----SEQ-VTP---------AVGVERFNQAMMDLGAMICTRSKPKCS----LCPLQNGCIAAANNSWALYPGKKPK-----  225
sp_P83847_MUTY_GEOS    147  VMRVLSRLFLVTDDIA------KPSTRKRFEQI----VRE-IMA---------YENPGAFNEALIELGALVCTPRRPSCL----LCPVQAYCQAFAEGVAEELPVKMKK-----  231
c_000003652391_2       150  VERVMARLFDISTPLP--------GAKPALKAR----AAS-LTP---------KLRPGDYAQAVMDLGATICTARAPTCG----VCPLNDCCHARRAGTAAELPKRTPK-----  232
c_000000031207_6       139  VERVLSRYLKIDGNPK------SGTARKRIQQA----ADD-ALD---------QENPGDHNQALMDLGRSICTPRSPDCQ----RCPLASGCGARASGEPTRWPTRRQK-----  223
c_000000430975_4       151  VERVVSRIFQITQPIP--------LSRPTLRQL----AAG-LTP---------SKRPGDYAQAIMDLGSLVCTPRKPKCN----VCPLHDICLANISGTPSDFPKRTPR-----  233
c_000001007148_4       148  TARFYARLLALRSDLH------SGEGRRQLWSL----AER-IVP---------RDRPGEFNQALIDLGATVCGV-TPRCQ----ECPVRGSCRSLELGEVERIPARASR-----  231
c_000001092848_3       161  VERVITRLRAIGTPLP--------AAKPQVRKA----VAA-LVP---------AERPGDFAQAMMDLGATICTPRRPSCI----LCPLQAFCAARGAAAQESFPVKASK-----  243
c_000001151029_1       108  VERVVSRIFQITQPIP--------LSRPTLRQL----AAG-LTP---------SKRPGDYAQAMMDLGSLVCTPRNPKCN----VCPWHDICLANISGTPSDFPKRTPR-----  190
c_000001797282_1       170  VERVLSRAYAVEAPLP--------GSRPEIRRL----TQA-LVP---------PDRPGDFAQALMDLGATICTPKRPACA----LCPWMRPCRARSLGTQESFPRKIKV-----  252
c_000001803648_25      153  IERVMARMHDIHSPLP--------SAKPELLAC----AAA-LTP---------DFRAGDYAQAVMDLGATICTPKSASCG----VCPWSFACLARINGTAAVLPKKTPK-----  235
c_000002078955_4       153  VERVMARLHNIHTPLP--------AAKPELFSK----ATA-LTP---------DLRAGDYAQAVMDLGATICTPKSPTCG----ICPWSCACVALISGTAAELPKKTPK-----  235
c_000002106160_4       141  VNRVMSRLLRLLRFTP--------YNKKRVLST----LIR-WLD---------RSRPGDFNQALMELGSTVCLPKNPKCE----NCPLKLSCGGTLSGSPESYPSPKPK-----  223
c_000003607531_2       151  VERVITRVFGIFKPLP-----EARPIIKDCAAK----LAP-KTT---------RGRPGDYAQAVMDLGATVCRPRKPLCD----LCPWEKYCIANQQNFVDQVPRKAPK-----  236
c_000003872363_4       149  VERVMARLFNVITPLP--------AAKPELTAH----AAA-LTP---------VDRAGDYAQAVMDLGATICTPKSPGCG----ICPCTEYCAALPEGDAANLPLRPPK-----  231
c_000004820107_1       156  IERIMARLYAITEPLP------DSKANLKNLAA----GLS-EER---------KDRPGDYAQALMDLGATICTPTSPKCS----LCPVNDSCTAKRQGIAESLPIKKKK-----  240
c_000005774797_1       140  INRVLSRVYNIDDAIN--------RPSARLARI----AET-LAH---------PKRSGDVAQAMMDLGAAICKPKNPNCE----TCPICDQCKAHSLGRTHELPVKAPQ-----  222
c_000005867021_3       153  VERVMARLYNIHSPLP--------AVKPMLLTH----ATA-LTP---------EFRAGDYAQAVMDLGATVCTPKSPTCG----ICPWSFDCIAHAKGTAAQLPKKTPK-----  235
c_000004546210_2       144  VKRVLSRFFGIKGWSG------ESKVSKELWDL----SAK-SLP---------VDNFQVYTQGIMDLGATICLSRNPNCS----ICPIQRRCYAHQNHLVDEIPSKKPK-----  228
c_000004551008_5       144  VKRVLSRFFGVKGWSG------ESKVSKELWDL----SAK-SLP---------VDNFEIYTQGIMDLGATVCLPKNPNCS----NCPIVNKCYSYLNQLVDEIPSKKLA-----  228
c_000002511148_4       139  VKRVLARHFKIEGKLE------TSSKRKELWQI----SED-LLP---------DNKIEVYTQAIMDLGATLCIKSSPACN----LCPLNQDCLALKEDLVTVLPSKTKP-----  223
c_000000577378_2       127  VKRVLARYFKIEGKLE------TSSKRKELWQV----SEG-LLP---------DNKIEIYTQAIMDLGATVCTKSNPVCN----LCPLNQDCLALKEDLVTVLPSKTKP-----  211
c_000000598175_2       150  VKRVLTRVFVVDGWPG------KADVLKKLWLL----AEQ-TTP---------ASRNADYTQAIMDLGATLCTRSKPRCE----DCPLKADCLAFQQGNQTVYPTSKPK-----  234
c_000000754627_3       147  VKRVLARFFMVEGWYG------VKKVENQLWHL----SEQ-LTP---------KNNVTEFNQAMMDLGASLCSRSRFDCE----ACPLNSRCGAFNAGKVKEFPHSKPK-----  231
c_000000811118_1       138  VKRVLLRISGSKSPMN------KYKTEKSLKEF----ANI-LLP---------NSKHKEYSQGLMDLGAIICRPNNPNCS----ECPVDEDCAANQKEIQDEIPIKITK-----  222
c_000000899687_2       142  VKRVLSRLFCINKNGT------TSAFENCLWGE----AED-LLP---------VRRPGDFNQALMELGATVCIPASPICQ----QCPLRTICKAFLKNKVNEFPPSKNK-----  226
c_000001176522_24      161  VRRVLCRLFGVEGWPG------ERQVEAHLWTL----AEC-LLPA--------QSEVMPDYTQAQMDLGATLCTRTKPRCE----ACPFQAECVAYRQGRVAELPAPRPR-----  247
c_000001345122_1       117  VKRVLCRFDAVTSWSG------NKKTEKVLWDR----ADE-FTP---------QERFDDYTQAIMDLGATLCTRSKPKCD----VCPVEADCDAKKMDKVIQFPYSNPK-----  201
c_000001515736_6       131  VRRVLTRVLAFDGDLA------QSRNERQLWEY----AQQ-LCPTE-----NLHEAMPRYTQGMMDLGASVCTSRKPTCL----VCPLDSECRAARAGNPENYPVRTRR-----  219
c_000001682161_6       138  VKRVLCRFDAINSWSG------NKQTESQLWQR----AEQ-LTP---------SKRVKDYTQAIMDLGATVCTRSKPSCE----ICPVAENCQARQLNQVAKYPFSKPK-----  222
c_000001923643_29      141  VRRVLTRVLAFDADLA------VARNERELWDL----AQQ-LCPTE-----DLQQAMPRYTQGLMDLGATICTPRKPSCL----VCPLQPQCRAARFGNPENYPVRTRK-----  229
c_000002030994_5       139  VKRVLSRVFTVVGWPG------KADVLKKFWVL----AEQ-TTP---------KTRNADYTQAIMDLGATLCTRSKPRCE----DCPLKTDCLAFQQGNQTEYPTKKPK-----  223
c_000002038721_4       141  VKRVLLRVLGNKSPIN------HSKTQKELKKF----AAE-LLP---------KNDFRTYSQGIMDLGSMICTPINPTCK----NCPIQNKCKSFQLNLQNMIPVKTSK-----  225
c_000002523527_15      156  VKRVFARVFGIHGYPG------ERAIENRMWAL----AEQALPPAG----RHQADHMVAYTQGLMDLGATICSRGKPACLADAEACPLVADCVARKDNLTAVLPTPKPR-----  250
c_000002608528_2       139  VKRVLTRVFTVEGWPG------KSGILKKLWLL----AEQ-TTP---------QTRNADYTQAIMDLGATLCTRSKPRCE----DCPLRAGCLAFQQGNQTDYPTKKPK-----  223
c_000002961510_2       142  VKRVLSRLFCINENGT------TSASENRLWQQ----AED-LLP---------VRRPGDFNQALMELGATVCIPKSPICQ----QCPMRTLCKAFLKNKITEFPPSKKK-----  226
c_000002979152_8       147  VKRVLARYHAIEGWPG------KSAVLKELWEK----AEL-HLP---------AERNADYTQAMMDIGATLCSRSKPACE----RCPVVDGCAAARKSSWALYPGKKPR-----  231
c_000003248034_1       159  VLRVLTRVLDEKGNVA------STAIQQKLTAA----ARL-LVES------VGPHSAGTWNQALMEVGATVCTPQSPSCD----RCPINRWCSAWKAGTQKTRPVKARP-----  246
c_000003294679_3       144  VRRVFTRHKAIKGWPG------TTMVQNVLWKI----AED-NTP---------SNRVADYTQAIMDLGATICTRSHPRCE----LCPVNKDCIAHSRGQEAEFPHKNIK-----  228
c_000003333364_1       141  VERVLARLFDVDAPIK------SSQAQSFLWET----ARS-LIP---------TGQAREFNQAIMEFGNLVCGR-KPSCM----FCPLTEECEAHYLGIVEHRPVPAER-----  224
c_000003402697_5       142  ISKIITVLWPG----------------QDTVEV----ANE-LVS--------LSQSGYAWNSAMMDLASSLRAGNPVE-------GALGETYFSDEAICRLFKPKRKKPEKKIK  219
c_000003492925_4       134  VKRILCRLHKLKTP-----------SDKELWRI----AYN-LVD---------KKNPFDYNQAMMDIGATLCTPKKPDCE----TCPLSDICQGQNDPTLYPTKKKR-------  211
c_000003766664_5       154  VIRVLSRLCDLEEEVG------ETKAKERIWDW----AER-LVD---------PQNASAFNQGMMELGAMLCTPTSPGCD----QCPWREDCRARAAGTVLERPIKKAK-----  238
c_000003948186_72      141  VKRVLSRVHRVAGWAG------RASVNKELWQL----AER-YTP---------KRRVERYTQAIMDLGATVCVRGQPRCH----ECPVAKHCEALRHDCVQSFPQPKAR-----  225
c_000005057120_4       140  VKRVLTRYFAISGWTG------NSKISNELWEI----SSR-YTP---------KIRVADYTQAMMDLGATLCTRSKPQCT----ICPIQADCLAKKQNKTAELPTPKPA-----  224
c_000005590109_5       140  VRRVLARFHVISGWTG------GTKVSNKLWQL----SSD-YTP---------LLRIADYTQAIMDLGATICIRSKPKCT----QCPINSACLAKIEGKISELPTPKPK-----  224
c_000006063368_2       139  VKRVLTRHRKIETDLS------KSSTLKKLWKL----SEL-LLP---------DVRIDTYTQAIMDLGAIVCKKKSPLCE----ICPVNKDCLALKANLVDCLPVKRKI-----  223
c_000000467631_2       119  VKRVLARYHAISGWTG------SAKISSELWKI----SAR-YTP---------AERCADYTQAMMDLGATLCTRSKPRCG----ECPISSDCVARIGDKVKLLPTPKPA-----  203
c_000000788235_1       140  VKRVLTRYHAIEGWSG------KGHILKQLWSI----AES-HLP---------DTRYANYTQAMMDLGATVCTRSKPKCL----GCPIHADCSAFQAGTPTAYPTPKPK-----  224
c_000001187176_11      141  VKRVLARYHAVQGWPG------EKPVHDRLWAI----AEA-HTP---------HSRVAHYTQAMMDLGATLCTRSKPSCL----ICPLQAGCQARMLGEPTAYPQARPR-----  225
c_000001660697_64      141  VKRVFARVFGIDGFPG------DKRVEDTLWRI----AEAVLPP---------ADGIQPYTQGLMDMGATVCTRGKPACLTGERACPLESLCEARSTDRVMELPVPRPR-----  230
c_000003378864_2       152  VKRVLSRLYDVSDP-----------VGTAAATRTLWAYSTELLN--------QADDAYTHNQAIMELGATLCTPKKTACT----RCPVSVRCDALARDTVPLRPIKRPR-----  237
c_000003910742_3       141  VKRVLTRYHAVEGWTG------KSQVLKQLWLL----AES-HLP---------ATRYANYTQAMMDLGATVCSRTKPDCD----HCPLNSNCAALSKGNPTAYPTPKPK-----  225
c_000004116181_2       107  VKRVLARFNAVQGWPG------NASVNKQLWAI----SAR-YTP---------VERVADYTQAMMDLGATVCTRTRPGCQ----ICPVNENCLARKKSLTEIIPASRPV-----  191
c_000006223903_1       142  VKRVLSRYHAVEGWSG------RAEVARRLWSL----AEA-HTP---------TRRVAAYTQAIMDLGAMVC-RRRPDCA----TCPLAGGCKAHKQNQVHDYPASKPT-----  225
c_000001029068_2       114  VKRVLARYHQVDGHYG------QGKTIKQLWKL----SEK-HTP---------NTRNNDYTQAIMDLGSIVCTRLNPVCD----CCPVALRCLAKKTHTQHLYPASKPK-----  198
c_000001128125_11      149  VKRVLSRYFGIHGWPG------EKKIEADLWHK----ADL-LTP---------PKRFADYTQAIMDLGATTCKRSKPLCG----VCPVQKSCVALKREETHSIPASKPK-----  233
```

```
c_000001595844_4      140  VFRVLARYTANFIDIG------TPAGKKEFTLL----ANQ-LLD---------KKQPGKFNQAIMDLGALVCKPTNPDCS----NCPLQIKCQAFSQDTIAQLPVKEKK-----  224
c_000001719155_2      144  VKRVLSRLFLLKENGE------TRKSENILWET----MQQ-LLP---------ETGAGNFNQAIMELGATVCLPKNPLCL----LCPLKRNCQAQKSGKQNLYPPRKRA-----  228
c_000002363038_6      149  VKRVLSRYFAIEGWPG------EKRVENQMWGL----ADN-LTP---------ETDFADYTQAIMDLGATLCKRSKPNCN----LCPVKTDCRALKTDQVASFPNSKPK-----  233
c_000002561400_1      105  VKRVLTRFFGVHGWPG------EKQVENRLWHR----ADE-LTP---------SVRFADYTQAIMDLGATLCKRSKPNCI----TCPVHSGCQALKKGEVHLLPSFKPK-----  189
c_000002566035_2      141  VKRVLTRYHGIKGWPG------EKKIENKLWSL----AEL-YTP---------RKLTKQYTQAIMDLGATVCTRRKPDCD----ICPLTLTCFAREMGKQHDFPGKKPT-----  225
c_000002598045_3      139  VKRVLARHFKIEGNLQ------ASSTTKQLWQI----SES-LLP---------GDRIDIYTQAIMDLGATVCTKSNPLCS----NCPVNQDCSAFKEGLVDTLPLKVKR-----  223
c_000004188575_41     141  VKRVLARFYAIASWPG------NKATEQQMWQQ----ADN-LLP---------DQRIAAYIQAQMDLGATLCTRSKPNCD----CCPLQSNCQAYARGTPTDFPIAKPK-----  225
c_000000240049_1      139  VKRVLARHQAIEGWSG------QSPVSKKLWTI----AEQ-FTP---------ENQLADYTQAIMDLGATVCTRSSAKCG----VCPLSEDCLAYQQQRVADFPGKKPK-----  223
c_000002139456_2      139  VKRVLARYQAVEGWSG------QSQVSKKLWAI----AEQ-LTP---------KEQLADYTQAIMDLGATVCTRSTAKCG----GCPLSEDCLAHQQQTVADFPGKKPK-----  223
c_000003535614_2      141  VKRVLARYQAIEGWPG------KPAIEKQLWVF----AER-YTP---------TEELADYTQAIMDLGATLCTRASPKCG----SCPLNNGCKALANNCVAALPTRKPK-----  225
c_000002657784_4      138  VKRVLARYKKIEGDLS------KALNIKTLWRI----SES-LTP---------EEKVDLYNQAIMDLGALVCTKSSPKCR----ICPVSRDCIAFNKKLIQVLPRKKLT-----  222
c_000003800129_15     143  VKRVVGRLYNQESY-----------QEKKFWEL----SKK-ILD---------KSNPFLFQQGIMDIGATICHIHNPQCD----ECPLGRWCLSKKNGSFYTYKKKK-------  220
c_000003716781_1      130  VRRVLARWFAVEGVV------TKAEPRREIEGL----AAA-LVG--------GVNDPGSWNQALMELGATVCLPKDPRCD----SCPVMTECRARARGLERAVPELPVR-----  215
c_000001556689_3      131  VARIMCRLAGIADDTR------RSKVRQRLEAM----AAD-AIE---------CHPPGTFNQAIMELGARVCTPRSPRCD----QCPCSSYCEAHELGIEEQIPPRRSK-----  215
c_000001834452_1      108  VCRVAARFLGLKSPLG------SSALRGQARDW----GET-LHAG------VSAGLAGSLNEGLMELGATVCRPRAPLCG----TCPISEKCVARATNRVTEIPKKAKR-----  195
c_000004255004_2      148  ARRVYARLLSLTN-------------LRAINET----AEQ-MVS---------HSRPGDFNQAVMELGATVCLPGKPRCE----RCPLAYRCQARSVGAFKLQLRRPTR-----  225
c_000000605438_3      141  VKRILYRFFAVKEA-----------NDKKLWEM----AYD-LYD---------KDNAYIYNQTMMDIGSAICTHKNPLCT----QCPFVSLCQGKDEPL--LYPTKKKK-----  218
c_000000581237_15     141  VKRILYRFFAMTSC-----------NDKKLWEM----SYE-LYD---------RENSYIYNQTMMDIGSAICTHKNPACD----VCPFEPLCQGKESPL--LYPEKKVK-----  218
c_000002529579_2      141  VKRILYRFFAVKEA-----------NEKKLWEM----AYD-LYD---------KENAYIYNQTMMDIGSLICTHKNPLCT----QCPFVSLCQGKDEPL--LYPTKKKK-----  218
c_000006097838_1      114  VARVLTRLLGIERPSK------QKQVVEQLWSV----TAD-LVKHAVDLQKTSRRNATAFNQAMMELGALICTPRNPLCH----DCPLKGHCIARKQNKVNRIPVSAKR-----  207
c_000000583727_6      140  VARVLTRLLGIEQSPK------DKPVTDRLWSV----ADE-LVKHTVTLQKPRQRNASAFNQAMMELGALVCTPRNPLCG----DCPLKGRCTARKQNKVETIPALAKR-----  233
c_000005807640_2      142  VARVLTRLLGIEESPK------ARPVADRLWSV----ADE-LVNHAVTLRKPSQQNASAFNQAMMELGALVCTPRNPLCG----DCPLKGRCIARKQNKVETIPALAKR-----  235
c_000000134878_1      124  VNRVVSRFYAYQDPPS--------KNKKKMEKF----MSL-IIN---------DNRPGDINQAIMDLGRYVCKPSTPLCD----ECPIHNGCKALKLGIQSDLPIKIKK-----  206
c_000005136725_3      139  VNRVVSRFYGYRDPPL--------KNKKKIEKF----MNA-IIN---------DKRPGDINQSVMDMGRYICKPRTPLCD----RCPIQYGCKALKLGIQADLPVKIKK-----  221
c_000001684786_4      137  VERVMTRILRLAENPR------QVSTKKRLKEI----AGG-WMP---------ADKASSFNQAMMELGALICSPVSPDCP----SCPVREVCAAAEQGDPENFPRLPPK-----  221
c_000002971826_23     147  VERVLTRILRLADNPR------TAATKKYLKEI----AGG-WMP---------RGKASSFNQAMMELGALICVPVSPNCA----ACPIHDACAAAEHGDPENFPKLPPR-----  231
c_000002040695_2      150  IARVFSRVLQYPGSKE--------ALTTSLRQP----ISL-FLP---------FKRIGDYTQALMDLGALLCRPCNPQCS----LCPIKTLCKSAHHNTVHLFPHKTKK-----  232
c_000003263657_32     154  VIRVLSRLYRIHEDAT------KGPAKRRFQEL----ADG-LLQ---------KGQAADFNQAMMELGATVCLPQKPRCT----VCPVQCHCRAFHELSDPSGLPYKPP-----  238
c_000005989041_2      151  IERVITRIFGICTPFP-----EAKTIVKDCAAK----LAP-KTT---------RGRPGDYAQAVMDLGAIVCRPKKPLCD----LCPWKKCCIANQRNFVDQVPQKVSK-----  236
c_000002391082_2      149  VTRVLCRLLRIEEDPR------RTAIEAELIAA----GEA-LLA---------RGEAGDFNQAMMELGARVCTPRNPLCP----TCPVGQWCRAQAELDDPSALPYKVP-----  233
c_000003839553_2      156  VIRVLCRHLRIEGDPR------RAQIKAELIAA----VEA-LLA---------RGQAGDFNQAMMELGARICTPRKPLCE----TCPVGATCRARAELDDPTALPYKAP-----  240
c_000003996707_2      146  VTRVLCRLLRLEGDPR------KAAVKTELIAA----GER-LLA---------RGRAGDFNQAMMELGARICKPTSPACG----QCAVVEMCRAHAELDDPAQLPTKVR-----  230
c_000002044706_1      129  VIRVLTRLFRIPGDPG------TAAVSRRLWQL----ATD-VLP---------QNRPGDFNQALMDLGATVCKPIAPLCE----QCPLAGLCAVRAAGNAEDFPNAGRR-----  213
c_000006007075_1      144  VERIIKRILNLKTEKE--------ISKENIIKK----KKI-LGM---------SDRSSDYAQALMELGALVCKPKNPYCK----KCPITKNCLSYKKKDFEI---ISKN-----  223
c_000002094036_4      120  IERVLKRILNLKSEVK--------TKKENLHKQ----KKV-FGQ---------TNRSNDYVQALMEIGALICKPKIPHCN----KCPITKNCLSYKKKDFKI---KPKN-----  199
c_000002762689_1      144  IERVLKRYLYLKKENE--------INKENLIKK----KKI-FGY---------SNRASDYAQALMELGALICKPNNPHCE----QCPISNKCIALKKKDFLL---TKIK-----  223
c_000005516980_2      144  IERVLKRYLYLKKENE--------IQKENLIEK----KSV-FGL---------SQRSSDYAQALMELGALICKPSNPLCN----QCPISKKCKSFRKKDFNL---IKNT-----  223
c_000003920004_1      150  IKRMLARLYGLDQSIN--------LINKKITSL----SKF-YES---------KKQSSNLIQAFMDYGSIICVPRNPKCG----ICIISRECIANQRKISNIIPKKIKS-----  232
c_000004008511_2      144  IERILVRLYGLKLPII--------KIKNDLRKK----SDN-FIS---------KNSSSDLIQAFMDFGSLICTPRNPDCV----NCLIQKNCNAFNYNLQDIIPVKLKS-----  226
c_000004481347_1      151  IKRIIARLYGIKTSLL--------LNKKTIEDI----ASK-YQS---------SNKASDLIQSFMDYGSVICLPRNPKCD----ECLIEKFCEARKKNIQHLIPFKNLS-----  233
c_000003787733_3      143  VKRVMARILKIKHITK--------RNNRRIRNR----LIK-WMD---------PERPGDINQALMDLANKICRVDHAYCN----TCPIDKVCMAEKMSIPESYPTKLNK-----  225
c_000000754657_2      143  VRRVMSRLLRIKNITK--------RNKKRIHNT----LIE-WMD---------RERPGDINQALMDLANKVCRVDHAQCN----SCPIDEICMANKMSIPESYPTRIKR-----  225
c_000000990943_3      143  VRRVMARVLRIKNITN--------RNKKRINNT----LIK-WMD---------PVRPGDINQALMDLANSICRVDHAHCS----ICPIDEICMAEKMPIPESYPIKVKK-----  225
c_000004474996_2      142  VIRVMARLLRIKNITK--------RNKNRIYNT----LIK-WMD---------KERPGDINQALMDLANKICCVDHALCS----TCPIDEICMANNMSIPESYPAKLEK-----  224
c_000001463500_11     130  VTRVLSRILAIRNLTP--------YNRRRIFNV----LEK-FID---------KERPGDVNQALMDLGSSICTPINTECI----ICPVSQFCKAKKTLLPFGYPIKINK-----  212
c_000001286181_5      137  VKRVVSRYLNVENNPR--------ELKKSIKPF----LYQ-NSP---------KANYQSFGQGMMDLGSLICVKEKPKCD----LCPIQKTCLSFKKQDFIKTKKNT-------  217
c_000001293628_3      143  VRRVLSRLFDLADP-----------TDASLRTR----AGE-LVD---------PDRPGDWNQALMELGATVCTARSPRCS----VCPLTKQCRAETEGTQELRPAPKRR-----  222
c_000001535696_8      137  VYRVLSRYFGVDLPIN------TTEGVKYYKEL----AFK-VMD---------SQNIRDYNQGIMEFGAIQCAPKNPNCF----SCPLSDSCVALQENKIDTLPVKLKK-----  221
c_000001614067_2      150  VERVFSRFYAVEKPIK--------ESKIFIKNI----AEK-HLP---------DNRHGDYAQALMDLGSLICIPKSPRCK----MCPLLAICDVGGTTSAKQYPIKLPK-----  232
c_000001765289_1      116  AKRVMSRILGIKNL--------TSWNLSRINKT----LSN-IIP---------EHTPGNFNQSVMELGATLCRPRSPSCN----KCPLSFGCKA-FKTNKPDYYPKPAA-----  197
c_000001961666_1      131  VERVITRLFKIDNNK---------KVKNKIKKY----TQS-LIP---------QNKPGDFAEALMDLGSLICKRANPTCS----LCPLKLNCKGYISNQIKVSVKNKKL-----  212
c_000002018097_4      138  LKRVISRYLGIKKLTR--------RNTLRIHNQ----LNK-MLT---------NGRPGDINQALMDVGSLICKPNDVICI----KCPLINKCKGYSYGNPIMYPDKNKR-----  220
c_000002843512_36     135  VKRILCRLQKLKTP-----------TDKELWKI----AYD-LVN---------KKNPFDYNQAMMDIGATLCTPKNPNCE----ACPLSDICQGQSDPALYPTKKKR-------  212
c_000002930199_1      135  VYRLISRLYNINTAIN------TNKGREEFQSI----ANN-LLP---------NKNTGLYNQAIMDFGSIQCKKYNPKCN----ICPLQKECQSAILGIVNERPVKTLS-----  219
c_000003033795_2      131  LERIGYRILGLKTKTK--------RNKKRVVEY----LEK-NQC---------ANNPGDYNEALMDLGSSLCKASITYCN----QCPLKKICKAYTSSSPILYPTPKVP-----  213
c_000003888107_2      132  LERIGFRLLGLKKRSK--------FNQNKVKKF----LEK-IQD---------KNNPGDFNQALMDLGSNICQSQSVNCR----VCPINDSCRAFLSLNPVDYPAPIKP-----  214
c_000004013286_6      157  SRRVLSRVFAIKGDLS------RQPMKHLLWDL----AKF-VLP---------EKNVGDFNQAMMELGSQCCQKQYPRCM----QCPIKEWCRAFQEGLQEELPFTPIQ-----  241
c_000004852258_1      119  HSRIIARVLGVKNQTS--------RNLNRINNY----LKK-LVR---------EGNPGEINQALMDMGSNICKSNKVICV----NCPFHFSCKAFQSGHPLSYPKKILP-----  201
c_000005254087_1      145  VVRVLSRLTDFAEDVT------TTPAKKQLWQM----AAK-LVP---------ADRPGDYNEAIMELGQTLCLPTKTDCV----ACPLTAVCLARQRGTQMERPVRPPR-----  229
c_000005603677_1      142  VYRVLSRYFGQALPID------SSAGKKWFKAK----AQA-LLW---------EDDPANYNQAMMDFGALHCRPKNPSCG----DCPLAKNCQAKANKQQLELPKKGKK-----  226
c_000003283462_3      130  IKRIMARMLGVKKITN--------FNYKRIKKT----LLN-LIS---------SSRPGDFNQALMDLGSQVCRPNQAHCY----RCPLKLFCKAGNTLNPEKYPRPLSK-----  212
c_000002687221_1      144  AIRVISRMNSIDSPYP--------KSRKKIINF----LSE-HID---------TIRPGYFNQALMDLGRVICTYKSPSCY----ICPVNTYCYSYVNNTVDKFPTKIKK-----  226
c_000003347358_19     166  VKRILCRLEKLRTP-----------SDTELWDI----AYT-LVD---------KINPFDYNQAMMDIGATVCIPKKPQCD----ICPLNDICKGQEEPTLYPTKKRR-------  243
c_000002701031_2      137  VIRVLSRYFGIQTPFD------SSDGKKEFQLL----AEE-LLI---------KEKAGENNQAIMEFGALQCTPKSPNCN----VCPLQNTCFAFNNNLILELPKKAKK-----  221
c_000000582753_3      145  VYRFISRLYGVSTPIN------SGKANKEFKVI----LNA-IID---------EKNPATFNQAIMEFGSLQCTPKSPNCE----SCTFSEICFAYKNNVIKDLPVKLKA-----  229
c_000001713769_5      137  VYRVIARFYGIDVPVN------NHLGKKFYMDY----AQK-LAP---------KKSCGDYNQGIMDFGSLICKPKAPLCD----KCMLAKDCIASKMKNVNYFPVRLKK-----  221
c_000004369364_1      140  VFRVLSRYTANFSDIG------TPVGKKEFTLL----ANQ-FLD---------KKQPGRFNQALMDLGALVCKPVKPICP----DCPLHSDCLAFAQDIISSLPVKAKK-----  224
c_000006057486_30     141  VYRVLSRYFGVSTPIN------SAKGIKEFKQL----AQI-LIN---------PKNPGTHNQSIMEFGALMCKPQKPNCT----NCPLNDSCFALQNNQIKTLPIKTNK-----  225
c_000005494072_10     137  VYRVLSRYFGIEYAIN------SSKGIKKFKTL----AQS-LLL---------NENFGLHNQAIMDFGATICTPKKAKCD----SCIFCESCSALQHNKVSELPVKTPK-----  221
c_000001742634_3      140  VYRVLARYFGIKTAIN------STNGIKEFKLL----AQQ-LLP---------NNKIGAYNQAIMDFGATICTPKKPKCD----ICIFNDSCIALQKNKITELPTKTNK-----  224
c_000002826998_2      140  VFRVLSRYFGIETPMD------TSEGKKQFTHL----ANE-LLL---------KKKPAIYNQAIMDFGAIQCTPKSPDCK----HCFLKKKCQALQENKISKLPVKSKK-----  224
c_000003159439_6      137  VYRVLARYFGIRTSTN------STKGIKEFKQL----AQE-LID---------TKDPATFNQAIMEFGAIQCKPKNPNCN----NCPLNTSCIALQKKLITILPIKDKK-----  221
c_000004887214_1      142  VYRVLARYFGIRTSIN------TSKAKKEFKEV----AFE-LID---------SKNPGLFNQAIMEFGSLQCTPKNPTCS----ICPLNDTCVALQTNQVEVLPKKQPK-----  226
c_000005037037_2      140  VIRVLSRFFGIENTFQ------TVKEKKEFQEI----ADV-LIS---------KKSAADYNQAIMDFGAQICTPKNYQCE----KCPISANCFAYKNKQQNNLPLKKKK-----  224
c_000001059964_1      132  MIRVISRMMNNQEEPR------LAKTRKRLWQL----ARE-WIP---------EGEARRFNQAMMEIGALICLPSNPKCL----LCPMNNHCRALQKGTVSLIPLKPPK-----  216
c_000002498472_1      141  VERVFARIFNIDLIPG------SPEARQLNWEK----AEE-LLP---------KGHARNFNQALMELGALICKPKKPLCT----SCPLVTHCLALKYDLVPERPVPKKS-----  225
c_000004615912_3      130  VYRVLSRFFGIKAAID------ISKNKKIFEVL----AQE-VLD---------ASQAGKYNQAIMDFGATMCKPKSPDCP----LCPLSKKCIALKEELVTTLPIKSKK-----  214
c_000004766858_2      143  IERVLCRIEDIGEPPK------STPVQKQLWSL----AAE-WIP---------HGHAREFNQGLMELGATLCTPRNPSCL----LCPANAWCVAFHTGRTDTIPAPRVR-----  227
c_000002747260_18     149  VERVMARLFQIETPLP--------GAKPELTEL----AGR-LTP---------DIRPGDHAQAVMDLGATICTPKSPACG----ICPVMTGCTARAAGAQADLPRKTPK-----  231
c_000005371561_38     149  VERVMARLFDIHEPLP--------AAKPVLKAQ----AAA-LTP---------ATRPGDHAQAVMDLGATICTPRNPACG----ICPLRAPCAARAAGTQADLPRKTPK-----  231
c_000000169465_2      145  VKRVLARYYSIGGWPG------QKKVENQLWEV----AEK-NTPTN-----SEGGRCANYTQVMMDLGAMICTRSKPKCD----ECPLQADCIAYAQGAQADYPGKKPK-----  233
c_000003254110_11     145  VKRVLARYYAISGWPG------QKKVENQLWEV----AEK-NTPTN-----PEGGRCANYTQVMMDLGAMICTRSKPKCD----ECPLQPDCIAYAQGAQTDYPGKKPK-----  233
c_000004750284_20     145  VKRVLARYYAIEGWPG------QKAVENALWDV----AEK-NTP---------QNRCANYTQVMMDLGAMVCTRSKPKCD----ECPLQRDCVAFAQGKQTDYPGKKPK-----  229
c_000000141782_15     150  VKRVLARYHAVAGWPG------ETAVSRRLWAL----AER-YTP---------DNRTADYNQAMMDLGATVCVRRRPRCG----VCPLADGCRARREGNPEAYPGSRPR-----  234
c_000002717847_8      144  VKRVLARLFLQQEF-----------KEGLFWNL----SAQ-CLD---------EDDPYSYQQGIMDIGATLCTPKAPLCP----ECPLNKNCLAFKENDFQLPSSKKPI-----  223
c_000004054799_1      131  VKRVLGRYKKISFKT-------ENEKQKKLWLI----SEE-LTP---------NKESFEYTQGIMDLGATHCSKSRPRCD----LCPVSNDCDSAFKEVSNKVSLRKAI-----  214
c_000006126673_1      156  IERVIARYHAINTPLP------KAKKEIKERAT----IYT-DNL---------PERPGDYAQALMDLGATICRPKSPLCY----SCPLKEGCTAYSLGQAEEYPKRQKK-----  240
c_000000339186_3      113  VIRVLTRIFGIRKNPK------DKATREYLWSL----AET-LVA--------KAENSSALNQSLMELGATVCTQRRPDCA----ACPVARSCTARRERATEIIPAKGEP-----  198
c_000003029168_2      145  VARVVSRLFALEGDLK------DTALQKKLWEI----TED-LVP---------NKRPGDFNQAMMELGAIICSTDSPRCT----LCPLAKPCHARVTGEPWRFPSESVT-----  229
c_000001863436_1      161  VARVLARLFAVDGDPR------SGDNQRRFWRL----AGA-LID---------RGRPGDFNQSLMELGATVCTPQGPSCL----LCPVREYCQAFATGDPTRYPAVAKR-----  245
c_000002830137_4      154  TTRVLSRLLAFHGDPS------QRVGQQLLWAF----AES-LLP---------RKRVGEFNQSLMELGSEICIPRKPNCE----RCPVRMLCAAHRQGEQLSIPISRRR-----  238
c_000004612302_3      148  TARLFSRLLALSVDPS------SSEGRGRLWSL----AER-VVP---------RTRAGEFNQALIDLGATVCGV-EPRCQ----ECPVRSSCRSLEQGVVDRVPVRSPR-----  231
c_000001933926_1      134  IERVMSRLHRLETPLP--------AAKPEIAEV----AQR-LTP---------RQRAGDFAQALMDLGATVCRPRRPNCG----ACPWNASCLAHAAGIQETLPRKLPK-----  216
c_000003136334_1      146  VERVTARLHRISKPLP--------GAKKYLKEK----AAE-LTP---------SERSGDYAQAVMDLGATVCTPRKPNCP----LCPWSYICKAYLAGDAEKFPKRVPK-----  228
c_000005849454_9      143  VKRVAARWAGLELAADDLALEKAARALGEAWMT----DLP-LGK---------LSAPGELNEGLMELGATVCSPRNADCG----ACPLASGCVALADGMVDVLPLPKRA-----  233
c_000001169194_1      154  VRRVLARLFDKREP-----------RARWLNRT----AAE-LVD---------KDRPGDWNQALMELGAMICSPKNPRCE----DCPVQLWCTAHEQGTQALSPAGVRR-----  233
c_000002786947_2      154  VRRVLARLFDKHEP-----------GVRWLNHT----AAS-LVD---------KDRPGDWNQALMELGAMICAPKNPRCE----DCPVQRWCTAHEQGTQALSPAGIRR-----  233
c_000001279808_52     142  ISKIITVLWPN----------------KGTVKV----AES-LVA--------AANSGYIWNSAMMDLASSLRAGNEIE-------AELGSTYFADVEVRDQFKPKRKKPSRTAK  219
c_000000358065_2      144  IERVLKRILNLKTEKE--------TKKENLHKL----KKV-FGI---------TNRSCDYVQALMEIGALLCKPKNPYCE----NCPITKNCLSFKKKDFEI---KKKN-----  223
c_000003745941_1      133  VIRILCRLTADNTVYT-----DNTQAMKVFTPL----AEV-LLD---------TKTPGQHNEAMMEFGAMVCSKVNPTCD----ICPFNSVCLGRNHQPER-LPIKAAK-----  217
c_000004187032_4      144  IERVLKRYLHLKKDNE--------IQKNNLIKK----KSI-FGI---------SSRASDYAQALMELGAMICKPKNPECS----ACPILKSCKSYKKKDFDL---AKIR-----  223
c_000006067315_3      144  VERVLKRVFYLKNEKE--------ISKENLINK----KFF-FGK---------SNRSSDYAQAIMEIGALICKPANPLCF----ECPISKNCRSFKKNDFEI---TSKN-----  223
c_000005543774_1      114  VKRVLLRLFNLETKD----------------------PAN-LFK---------TKRNGDLAEALMEFGAIICKPKDPLCY----KCLLKKNCLYYKSESKI-RFNKKIK-----  185
c_000006211484_1      136  VKRVLFRLDNININD----------------------AVN-LFK---------TQRNGDLAEALMEFGALVCRPKDPRCH----ECKIKKMCAYFKSKSKI-KFNRKIK-----  207
c_000002718976_3      145  VRRVFSRNLNIEENK--------INFDKFIKKN----KKK-FFI---------TKRNDDFVEALMEFGALICKPKDPNCL----TCCLNKTCKYFKSSKKI-KNIKNKM-----  226
c_000002992548_1      146  IKRVFSRLF--NANL---------EKNEKRIKRI----TKE-QLY---------TDRCGDLAEALMEFGAVICKPSNPMCN----ICNLKANCYFFKNKTSV-LVNKKRR-----  225
c_000000456751_1      110  VKRVFSRLLLINTSN---------QNIDNEIKKI----TYK-LPK---------IDRNADFAEALMEFGALVCKPQNPLCK----ICHLQKYCKFFNSENYI-SLNKKFS-----  191
c_000002655634_1      141  VKRFFSRLFLIDVSS---------QNFENEIKKI----TNK-LPK---------VNRNADLAEALMEFGAVVCKPKNPLCK----ICNLKKYCKFFNGENYT-LPNKKFF-----  222
c_000004232403_2      140  HRRVISRILGIKNLSR--------HNKNRINNY----LKQ-LVA---------IDRPGEINQAIMDIGSIICKPKEVFCD----FCPVQFSCKACVSSKPLEYPNKIYN-----  222
6u7t.after.N146.pdb   141  VMRVLSRLFLVTDDIA------KPSTRKRFEQI----VRE-IMA---------YENPGAFNEALIELGALVCTPRRPSCL----LCPVQAYCQAFAEGVAEELPVKMKK-----  225
Consensus_aa:              l.RVhsRhh.lp.............ppph..h....s...hhs.........ppssp@sQAhM-lGthlC..ppP.C......CPl...C.t........hP.........
```

```
Conservation:                                                                     5   5
sp_P17802_MUTY_ECOL    226  -----QTLPE-------RTGYF----LLLQ-----HEDEVLLAQRPPS-GLWGGLYCFP---QFADEESLRQ---------------------------WLAQRQIA------A  281
sp_P83847_MUTY_GEOS    232  -----TAVKQ-------VPLAV----AVLAD----DEGRVLIRKRDST-GLLANLWEFP---SCETDGADG-----------------KEKLEQ----MVGEQYGLQ------V  294
c_000003652391_2       233  -----QPKPT-------RYGIV----YLARD----AHGAWLLERRPDK-GLLGGMLGWP---TSDWNDAPG------------------------------DQPPFA------A  286
c_000000031207_6       224  -----RTTEQ-------QWWAA----AVVV------CGDQVLVWIGDG-ELLAGHRGTP---LARLSGSGL----------------DADKMIG----KEFKRLGLD-----GA  286
c_000000430975_4       234  -----RKKQR-------RYGVV----YWIER----RDGAISLRQRPEN-GIFGGMTEIP---GSLWKASPL--------------------SRH----SIHAQAPIP------A  293
c_000001007148_4       232  -----RDVTD-------VFELL----VVMR-----RGKRLLLIQHPDG-ERWGGMWGFP---ALSRDRALARS-------------RFDERLIE----SEVADWGCD------V  297
c_000001092848_3       244  -----AAKPS-------RRGAA----FVAVR----ADDAVLLRRRPES-GLLGGMSEVP---TSGWSAGSD----------------GEDGP---------SAASFP------A  302
c_000001151029_1       191  -----TKKRK-------RYGVV----YWIER----RDGAIVLRQRPEN-GIFGGMTEIP---GSLWQAAPL--------------------SRH----SIHAQAPIP------A  250
c_000001797282_1       253  -----AKGAL-------RRGAA----FVAIR---SGDEAVLLRTRPPE-GLLGNMAEPP---GSAWEPDYD----------------VAAAL---------LDAPLD------A  312
c_000001803648_25      236  -----PVKPT-------RYGIA----YIARR----GDGAWLLERRPDK-GLLGGMLGWP---GAQWGDQAV------------------------------DKPPVS------A  289
c_000002078955_4       236  -----PVKPI-------RYGIA----YIARR----TDGAWLLERRPDK-GLLGGMLGWP---GAEWGAKAV------------------------------ENPPVS------A  289
c_000002106160_4       224  -----QSLPW-------QNVLT----GVIW-----KRDSFLIIKRAGY-KHLNNLWELP---GGRTLTKNN----------------PHPQLEE----LLKNKFKLE------V  286
c_000003607531_2       237  -----KVKAV-------RKGVV----FLALM----SDGSLFLRRRPEN-GLLGGMMEFP---STVWLERSL--------------------SPK----EICEAAPFK------T  296
c_000003872363_4       232  -----PAKPI-------RYGTA----YIARR----HDGAWLLERRPDN-GLLGGMLGWP---GSEWGEEAV------------------------------ENPPFS------A  285
c_000004820107_1       241  -----KPKPQ-------KRGHV----FWISD----DSGNILFIRRPEN-EMLGGMLGLP---TTEWDIRP---------------------------------IKLP------Q  291
c_000005774797_1       223  -----RSKKK-------WNAFA----YVIYN----ADGNIGFVRRDKD-ALLGGMWALP---TSDWSTSPV--------------------FQP-----------PI------L  275
c_000005867021_3       236  -----PVKPT-------RYGIA----YVARR----PDGAWLLERRPDK-GLLGGMLGWP---GAEWGAQAV------------------------------EKPPIV------A  289
c_000004546210_2       229  -----KKKRT-------ESMYF----LMIVF----DGKKLFLEKRKPK-GIWGGLWSFP---QIQLADDPQI--------------------------WCEQQFTNN------I  286
c_000004551008_5       229  -----KKKRT-------ESTYF----LMIIF----DGKKLFLEKRKPK-GIWGGLWSFP---QIQLADDPKT--------------------------WCEQQFTNK------I  286
c_000002511148_4       224  -----KSKPT-------KKVVW----LLPQG----PSGEVLLEKRKPK-GIWGGLWAFI---EAEKKNELEL--------------------------ELSRRFKTD------C  281
c_000000577378_2       212  -----KSKPT-------KKVVW----LLPQG----PSGEVLLEKRKPK-GIWGGLWTFI---EAEKKNELEL--------------------------ELSRRFKTD------C  269
c_000000598175_2       235  -----KVMPE-------KQAIM----VILKN----SKQEVFMQKRPPV-GIWGSLWCFP---QFEKRAFAEE--------------------------WLKSNYGKL------I  292
c_000000754627_3       232  -----KAVPK-------KSCHQ----LIIK-----HNDKVLMEKRPNS-GIWGGLFGFF---EFNEYSELET---------------------------FLAQQGLK-------  286
c_000000811118_1       223  -----KEKIE-------KKIDW----ILIK-----TKNKVLLKRNKEK-GIWQNLWVLP---EKDFFSSKKI--------------------------------SKI------L  273
c_000000899687_2       227  -----ISSKK-------IEVSA----GIII-----KNKKVYIQQRVKN-GLMGGLWEFP---GGKREQGES----------------PEECLKR----EIKEELRVN------V  289
c_000001176522_24      248  -----KVMPV-------RKVCW----QVSLS-----QGCVWLVQNPAR-GLWGGLWVLP---EGNR------------------------------------------------  288
c_000001345122_1       202  -----KIIPT-------KEKFF----LILVT----EAFQVALVKRPEK-GIWGGLWSLP---EFDSVQEIDL--------------------------FLDGNEMVAT----LK  261
c_000001515736_6       220  -----IKRSS-------ESWWL----LLAVD----ARRRVWLQKRPQA-GIWAGLYSPP---VFEDYAALQS--------------------------YAQAAWSQD-----GT  278
c_000001682161_6       223  -----KTIPT-------KERWF----LVLLT----KEGNIGLIKRPAK-GIWGGLWSLP---EFDEVKLLEN--------------------------FITDNNVKCL----LK  282
c_000001923643_29      230  -----LKRSS-------EAWWL----LIAVD----GQGRVWLERRPQQ-GIWAGLYAPP---VFNEREALEQ--------------------------AAARHWPAA-----RS  288
c_000002030994_5       224  -----KEMPE-------KHTVM----LILQN----DQQEVLMQKRPPV-GIWGGLWCFP---QFDKTALAEE--------------------------WLDDSFGMS------L  281
c_000002038721_4       226  -----SQKKE-------KNINW----LLIA-----SKDKVLLKRNVNK-GIWQNLWLFP---EANSLNYEK---------------------------------FNL------L  275
c_000002523527_15      251  -----AAIPE-------RSTVM----LIVRH-----GRDVLVQLRPSS-GIWGGLWSLP---EMPVDTVPFD--------------SEAAEESA----LELARAYGS------P  315
c_000002608528_2       224  -----KVMPE-------KQTVM----LILKN----GKQEVFMQKRPPA-GIWGGLWCFP---QFDNHENVLE--------------------------WLDNNYAVS------F  281
c_000002961510_2       227  -----VPSKK-------IEVSA----GIII-----KNKKIYIQQRSKN-ALMGGLWEFP---GGKREKGES----------------PEECLKR----EIKEELRVN------V  289
c_000002979152_8       232  -----KDKRI-------KQSVM----LYVSN----ENADVYLERRSST-GIWGGLWSLP---EFETNVKAND--------------------------WLVKTFALE------S  289
c_000003248034_1       247  -----RKKKR-------VMTSV----IACE-----QGGQILMKQRSVNARLMPGFWELP---EVEGPFLNQ----------------ADL-------------ARWS------L  301
c_000003294679_3       229  -----KTNPL-------KTTNM----LFLRF-----EDRIYLERRPSS-GVWGGLWSFP---EISKDCSVSD--------------------------WYQSRFGAY------P  285
c_000003333364_1       225  -----KPAVP-------VDVAS----GVLV-----HDGYIFIQKRPED-GVWPGLWEFP---GGTVEKGET----------------PAQCVVR----EFQEELEWD------V  287
c_000003402697_5       220  TEKPKKRKHR-------IEVGI----ACIW-----REGKYLVQARPKG-KSFEGSWEFP---GGKREKGEN----------------FRECVKR----EIQEEVGID------V  287
c_000003492925_4       212  ------VVPT-------REENI----MLYV-----YNDKLSLTQRKG--KFLHGLWGFE---NTEMPL----------------------------------------------  252
c_000003766664_5       239  -----RPPKK------IALVAC----VIRD-----EQGAVLLTQRPEK-GLFGGLWEVP---LKEVDETPS-----------------KAGVGR----LVKETVGLT------A  301
c_000003948186_72      226  -----NNIPT-------RHTQM----LLIEN----TAGEVWLEKRPAQ-GIWGGLWSLP---ELATGQNAIT--------------------------YCEQELQIA------V  283
c_000005057120_4       225  -----KVLAV-------KQLIF----LMLQD----QQTQFLLEKRPAS-GIWGGLWSFP---EFQSFAEIKS--------------------------WCLEN-DIA------M  281
c_000005590109_5       225  -----KSLPV-------KKITF----LMCQD----QQGRILLEQRPKT-GIWGGLWSFP---EYESLANIQS--------------------------CFLEK-GIP------I  281
c_000006063368_2       224  -----KTKTT-------KHVHW----LIPYS----HKGRFLLQKRLNK-GLWEGLWVFI---EKEKEEMLKK--------------------------ECKSIFQIK------D  281
c_000000467631_2       204  -----KTLPV-------KNIMF----LMLKD----ELGLVLLEKRPAT-GIWGGLWSLP---EFETLSEIQS--------------------------FCQQN-NFA------I  260
c_000000788235_1       225  -----KTLPE-------KKAVF----LLLIN----INHEIFIIKRPPS-GIWGGLWSLP---QFDTYLQAQQ--------------------------WYEQYFSTH------L  282
c_000001187176_11      226  -----KRIPV-------RQCLM----PLLVN----PQGDIWLQRRPDS-GLWGGLWCPP---QLDSREQLDA--------------------------LVIAQ-GWQ------A  282
c_000001660697_64      231  -----KAIPE-------RAATM----VIALH-----GETVLLQRRPQR-GIWGGLWSLPLVGEMDDALDAHP--------------------------LDADTVRQAALAYGTV  296
c_000003378864_2       238  -----KPVPH-------HHIGV----GVIWK-----DDQVLIQLRPPE-GLLGGLWEFP---GGKQEPAET----------------IEETVRREIREELGVD----------V  300
c_000003910742_3       226  -----KVIPE-------KNAVM----LMLIN----NKDEVFIMKRPSP-GIWGGLWSFP---QFDDYDQAQA--------------------------WHENYFGFP------C  283
c_000004116181_2       192  -----KKQAT-------RTCVF----ILIVN----AKQEILLQKRSPV-GIWGGLWSLP---EVEDTIEAIH--------------------------WCSSR-KIT------V  248
c_000006223903_1       226  -----RPRPR-------REVVM----IMIKD----SRDRVLLERRASS-GIWGGLWSFP---ECPVEEALEP--------------------------WCRMRFGAG------I  283
c_000001029068_2       199  -----KPKPV-------KTTAM----LIFDN----GKGGVYLQKRPAK-GIWGGLWSFV---ECSATDKAIT--------------------------KTAHNFHPQ------A  256
c_000001128125_11      234  -----KDKPV-------KRAFF----LLLLN----SERQVLLRKRPQK-GVWAGLWSLP---EFDSLQGFKE---------------------------SIESQTTV------I  290
c_000001595844_4       225  -----IKRKT-------RYFYY----FVFR-----KGSDYYIQKRTKK-DIWQSLYEFP---NIESKTPLN----------------DNEVKIV----FQEKYPRIK------F  287
c_000001719155_2       229  -----APSTK-------IAVSA----GVLF-----RRNRIYIQKRKAE-GLMGGLWEFP---GGKFESGES----------------PEQCLHR----EIKEELGVT------I  291
c_000002363038_6       234  -----KNKPV-------KQKWL----LLQKN----SAEQVFLYKRPQK-GIWAGLWSLP---EFDSYDRAVD--------------------------FLNGEIHRH-----DA  292
c_000002561400_1       190  -----KEKPV-------KQAWF----VVQLN----AKNQIRLIKRPQK-GIWAGLWSFP---EFCSVTDAKT---------------------------YLQTQTPG------L  246
c_000002566035_2       226  -----AVIPK-------RDTVF----AIMEN----NNGEILLEQRPSS-GIWGGLWCFP---EFSSPLRIEE--------------------------MIKRKYGFN------I  283
c_000002598045_3       224  -----KSKLT-------KKVLW----LLPQG----PSGEILLEKRKEE-GIWGGLWSFI---ETEKKAELEL--------------------------ALSRNFDSN------I  281
c_000004188575_41      226  -----KTIPT-------RQSHW----LILLT----DDRRVLLSKRPEK-GIWGGLWCFP---SFDDTHTL------------------------------EQHIDNT------K  279
c_000000240049_1       224  -----KILPV-------REKRL----LIIRN----KQGHYLMEKRPPS-GIWGGMWSLP---ELTLDKSIAE--------------------------SIERNWQLS------V  281
c_000002139456_2       224  -----KILPV-------REKRL----LIIRN----KQGYYLMEKRPSS-GIWGGMWSLP---ELTLDESIAE--------------------------SVERNWQLN------V  281
c_000003535614_2       226  -----KILPI-------QQKRL----LIIRN----EQGAYLMEKRPPT-GIWGGLWSLP---ELALEAPIIT--------------------------EVKNTWQII------V  283
c_000002657784_4       223  -----KQKPF-------KTVFW----LVVMN----KNGKVLLKKRNNL-GVWKGLWSFL---ESENIQDLGK--------------------------ECLTMFEKR------K  280
c_000003800129_15      221  -----TPKKK-------VFLNY----EIYK-----RDGCIYMIKNMDL-GFWNELWMPP---YKIVE-----------------------------------------------  262
c_000003716781_1       216  -----PTPID-------VELEM----FLVH-----DHGRVLLRRRDEG-GRMAGLWELP---TREVAAAELRLF----------PAELELEL----------------------  272
c_000001556689_3       216  -----LVKA--------VTEYA----AVIE-----DNGRLLLFRGQRP-SLVSDMWEFP---TLDSRLADTSSRSLTEKPASSHSRAQEAKLSR----YIKEQLGWS------V  293
c_000001834452_1       196  -----INWKE-------VHLLY----GVAS-----CPSGVLLEERKS--GWNQGLWEPP---SVPYDQKEE--------------------PDL----AWREFQPQR------G  253
c_000004255004_2       226  -------TKA-------VQWPL----TLAK-----WRSRILLHRRPDK-GILASLWELP---TPENLPAEL----------------IDETL----------------------  274
c_000000605438_3       219  -----IKKPI-------RKRAL----IIYA-----KNDKYALAQNEE--RLLSGLWGFK---QEEDFEF--------------------------------------------I  262
c_000000581237_15      219  -----KKKPI-------RKRTL----IIYT-----KDDKYALSQNSE--RLLSGLWGFK---QEEEFEF--------------------------------------------S  262
c_000002529579_2       219  -----IKKPI-------RKRTL----VIYH-----KANKYALAQNEE--RLLSGLWGFK---QEEDFEL--------------------------------------------L  262
c_000006097838_1       208  -----APVSK-------RRFIG----FIIA-----WHGNVFVQRRPSG-EVNSGFWEFP---NWLVDDTQ-------------------VQPAD----LASTKLALG------Q  267
c_000000583727_6       234  -----AATTN-------RRFIG----FLIA-----WRGKVFVQRRPSG-EVNAGFWEFP---NWQVNDAE-------------------LKPAA----LAKRELKLG------Q  293
c_000005807640_2       236  -----AATTK-------RRFIG----FLIT-----WHGKVFVQRRPSG-EVNAGFWEFP---NWEV-DAA-------------------ATADQ----LAKRELRLG------Q  294
c_000000134878_1       207  -----ETKPH-------YDVAV----GIIW-----DKKKLLITKRKEE-GLLGGLWEFP---GGKIKKNEK----------------IESAIKR----EIKEELSIN------I  269
c_000005136725_3       222  -----HTKPH-------YDVAV----GIIW-----DKQKLLITKRKEE-GLLGGLWEFP---GGKMKKNET----------------IKSAIKR----EIKEELSIN------I  284
c_000001684786_4       222  -----RKTIA-------VELEA----GIIR-----RGKRCLLERSESF-DFLEGLWLFP---LSLPGD---------------------GGIAA----RLGQALETD------I  279
c_000002971826_23      232  -----RKTVA-------VELEA----GIIR-----RGKRYLLERNEDL-DYLEGLWSFP---LARPDS---------------------GGIAA----RLRKRLATS------I  289
c_000002040695_2       233  -----TKKPC-------RWGHF----FFLVHIPSSGSPVSVLLEKEHG-PLLEGLWRPL---TQSWVHKKT----------------EAH--------------FPY------K  290
c_000003263657_32      239  -----KKERP------HHDHVA----GVIK-----LDDKFLIIQRPLD-GLLGGLWEFP---GGRRRESES----------------LTEGVTR----TILESTGLT------V  302
c_000005989041_2       237  -----KVKPV-------RKGIV----FLALM----SDGSLFLRRRPET-GLLGGMMEFP---STNWLERRI--------------------SPE----EICEAAPFK------S  296
c_000002391082_2       234  -----KKQKP------HYQVAC----GVIC-----KGDRLLLAQRPSA-GMLGGLWEFP---GGKQEEGET----------------LQQCLQR----EIAEELAIE------I  297
c_000003839553_2       241  -----KKKKP------HYQVAA----GVIS-----KGRQLLIAQRPAE-VMLGGLWEFP---GGKQEEGET----------------LEECLVR----EIREELGIE------I  304
c_000003996707_2       231  -----KPTRP------HYEVTA----GVIW-----KGSKLLVAQRPSD-GMLGGLWEFP---GGKREPGES----------------LQECLRR----EIREELTID------I  294
c_000002044706_1       214  -----TKVTF-------VQRVA----LFLEK----QDGSFLLVRRPAD-GLLAGMWELP---GRELGDGEAPE--------------RLASTLCG---EFGLTIRPD-------  270
c_000006007075_1       224  -----KKKID-------KYFLI----KIYK-----KNKKILLIRNTKF-NFLKNFKIFP---MEEIPKP---------------------------------------------  267
c_000002094036_4       200  -----KKIIN-------KFYLA----TLYK-----NQNQILLIKNDKF-KFLKNLLIFP---MREITYP--------------------------------------------D  244
c_000002762689_1       224  -----KKNNN-------KYYLL----KVYK-----DKNKYLLIKNTKF-NFLKNFSIFP---MQELSKP---------------------------------------------  267
c_000005516980_2       224  -----KKNIN-------KYFIL----KVYK-----KDQRYLLIKNTKF-NFLKNLRIFP---MEELFQP---------------------------------------------  267
c_000003920004_1       233  ----KKSRMK------KYTRAY----IIIN-----GSEEILVRRRASK-GMLPSMLEVP---NDKWVTEKK----------------LLVRD------SSVSLFK---------  292
c_000004008511_2       227  ----KSTKRK------KYSRAY----IFYN-----EKNEILIRKRPSK-GMLASMLEIP---NDNWVINKK----------------SLVTD------TIAIKLK---------  286
c_000004481347_1       234  ----LEQKKI------KFTRAY----IIMN-----KKNEVLVNRRKSK-GMLASMLEVP---NDEWVDVKK----------------KLKRH------DVYKDLS---------  293
c_000003787733_3       226  -----KIIPH-------KEIVA----GIIW-----QRDKFLITKRPEN-ALLGELWEFP---SAEIQFNET----------------PIGALRR----QISKDFNIY------I  288
c_000000754657_2       226  -----KVIPH-------KEIVA----GIIW-----QREKFLITKREEN-ALLGGLWEFP---GGEIASSET----------------PVGALRR----QIKEKWDID------I  288
c_000000990943_3       226  -----KVIPH-------KDIVT----GIIW-----RGKKFLITKRSEN-ALLRGLWELP---GGEFESNET----------------PIEALRR----KIKEECDID------I  288
c_000004474996_2       225  -----KVIPH-------KEFVA----GMIC-----QGNKFLITKRNEN-ALLGGLWEFP---GWEIVSSET----------------PIGALRR----KIKEEWAID------I  287
c_000001463500_11      213  -----SIKNI-------KHFIA----AIIW-----YNKEFLIMKRPVN-SMLGGLWELP---NTQIDLKIS----------------DDIQLFN----YIKNSLNFT------I  275
c_000001286181_5       218  -----VTKTQ-------ESFHW----FIYQ-----KNNKVMLCKNPDE-GIWPNLWVFP---KRELFQT--------------------------------------------K  262
c_000001293628_3       223  -----PRVRE-------ASYAV----LVTLN----PVGELLMVRRPPD-GLLGGLWEFP---AVEMEAEERNPWGGELR-VGDSLHGRCSARLE----DLGVQLSAR------R  301
c_000001535696_8       222  -----TKVRN-------RYFNY----IIPIAEADDGSRKTLLNQRKGK-DIWQNLWQFP---LIETKREID-----------------LEEVKK----RLCEVMHLAD---DPQ  291
c_000001614067_2       233  -----KEKEE-------RYGLF----FYLQN----KDGAVLFETNKSS-GLLANMDVLP---SIGWYEESN----------------RFKKSPK----FNKKKPNFL-----GM  297
c_000001765289_1       198  -----KKRKP------HYTIVA----GIIW-----RDNTFFIQRRPEK-AMLGGLWEFP---GGKVEEGES----------------LEAALKR----EIKEECGVV------P  261
c_000001961666_1       213  -----KTIRK--------GKCY----IIKRL----NDSKFLFIRNPAK-GLLGSMLLFP---TYGWLHSDH------------------DDYVK----SMIEKFIKN------I  273
c_000002018097_4       221  -----KPIPT-------KEYVA----VFIS-----YNDHIFINQRSED-GLLGGLWELP---MIEVFENSN----------------KIKALKT----YAKNKLGLS------I  283
c_000002843512_36      213  ------VVPT-------REQNI----MLYF-----YNDKLSLTQRTG--KFLHGLWGFE---AIEVPL----------------------------------------------  253
c_000002930199_1       220  -----RKPKI-------RYFNY----LFIK-----KNQQFLIQQRGTN-DIWKKLYELP---LIESKEKLN----------------REKIMA------HQYFKPFK------I  280
c_000003033795_2       214  -----KKIPI-------YDIAV----SVVE-----YQNKILITKRQNK-NFLPGLWEFP---GGKIEKKET----------------ATQAIIR----EVKEETNLT------V  276
c_000003888107_2       215  -----KTIPT-------IKVST----CIIR-----KQNKLLILQRPID-TMLGGLWEFP---GGKIQNNES----------------RKKAVIR----EVMEETNLA------I  277
c_000004013286_6       242  -----PKIQA-------VREVA----VIVR-----KGKRVLVVQRPSD-GRWAGMYDFP---RFECSFRSKE--------------KIRQLMVR----GVGQQTGVP------I  306
```

```
c_000004852258_1        202  -----KTIPT-------KTIAA----ALVN-----HGDNIFITKRPLK-GLLGGLWELP---NIELVNGEI----------------PEDLLKI----KFADQFGLT------I  264
c_000005254087_1        230  -----KRTPHYDVVAGIIWQNG----VSPG-----EGGRFLIAQRPLN-GLLGGLWEFP---GGKQEPDET----------------LPQALER----EIREEMDMM------I  299
c_000005603677_1        227  -----LKIKK-------RYFYY----FVWRG----TNGGYYLRKRQEK-DIWQGLYEFP---MIELDKPLK----------------TSELKT-----LWAHWELEE------L  289
c_000003283462_3        213  -----KNIPH-------YDVVV----GMIW-----KEGRFLIQKRENQ-KHLSGLWELP---GGKIENNER----------------SELALIR----EIKEECDFE------V  275
c_000002687221_1        227  -----VSRPH-------HNVAV----GLIW-----KDNRILISKRNAS-GLLGGLWEFP---GGKIRSGES----------------GSSCVVR----KTQEILNVL------V  289
c_000003347358_19       244  ------VVPT-------REENI----LVRV-----YDDKLSLTQREG--KFLHGLWGFE---SVEVPH---------------------------------------------  284
c_000002701031_2        222  -----LKIRK-------RYFEF----LIIN-----ENEKILIQKREK--GIWKGLYQFP---LIENSVKKSE--------------RDIENSKS----WKKLFKHNS------I  285
c_000000582753_3        230  -----RKSTN-------RYFTY----YYIS-----DSNHLYIKKRVGK-GIWESLYEFP---LSENLIAFE----------------NEVLDK-----QIFKNSIND------F  291
c_000001713769_5        222  -----NAPKI-------IHFNY----LVLL-----DSDHMICMDRIKN-GIWKNLFQFP---MIESKKELN----------------KTQVLSN----EIFKSIAPIS---NSD  287
c_000004369364_1        225  -----IKKRT-------RYFYY----LVFK-----EKDLFLIQKRTQK-DIWQSLFEFP---NIEGMEELN----------------ESEIREA----IQKKYPNLK------Y  287
c_000006057486_30       226  -----IKIKK-------RHFNF----IVI-N----NNQHTYIHQRKDN-DIWKNLYQFP---LIESKHEIDTN-------------KVIKSSIF-----QDFFKNNSY------T  291
c_000005494072_10       222  -----IKIKK-------RYFNY----LVVLS----KKNKITLNKRINK-GIWQNLFEFP---LIETVETITIK-------------TLVEHEYL-----EKIINKKQL-----VD  289
c_000001742634_3        225  -----IKIKK-------RFFNY----LILIT----KDNKTIINKRINK-DIWLNLFEFP---LLETKTEINEN-------------KLVKNDFF-----QKLILNDTT-----IN  292
c_000002826998_2        225  -----IKKKT-------RYFNY----LVLNF-----GKNTWVQKRTKK-DIWQNLYEFP---LLETDKIIS----------------QKELMKS----ELWKESVSDF----DF  289
c_000003159439_6        222  -----IKIKK-------RYFNY----LVIIN----SQNKIALQRRKHK-DIWKGLYEFP---LIETLTAIDIE-------------QLLETEEF-----HQIINEDQA------I  288
c_000004887214_1        227  -----LKIKK-------RYFNY----LILKT----KNEETLVKQRKGK-GIWQNLYEFP---LLETEKEINYL-------------ELIDHSIF-----KKMIEENNF------D  293
c_000005037037_2        225  -----VKVKE-------RFFYF----LVLN-----IDNTTYIEQRQNN-DIWKGLFQFP---MIETQQLIN----------------KPKELEK----LITNKINVR------F  287
c_000001059964_1        217  -----KKVKI-------LYRGC----AVIL-----FQDGLILCKRNQP-GLLEGLWEFP---SVELGKGDN----------------LRKALQE----HLLEELGKP------F  279
c_000002498472_1        226  -----KKIIP-------IDMAT----GILI-----HNGMLFIQQRLPD-DVWGSLWEFP---GGRMELKET----------------PEQTVVR----EFLEETEFK------V  288
c_000004615912_3        215  -----IRKKK-------RYFHY----FISHF-----EGKILLRKRIQK-DIWQNLYEFP---MLETKDEELK---------------ISRVQKT----QLWQQLFHGNEKVKVL  284
c_000004766858_2        228  -----PEITR-------IEKLT----ALVQ-----NGDCWLVRKRPEG-GLMGGLWEFP---TWEELPSENS---------------AAAWLTQ----QLHEDYGVA------A  291
c_000002747260_18       232  -----KPKPT-------RHGIA----YLGRR----ADGAWLLERRPDK-GLLGGMLGWP---GGDWAETAT---------------------------------EAPPID------A  285
c_000005371561_38       232  -----KPKPT-------RRGIA----YLGRR----ADGAWLLERRPEK-GLLGGMLGWP---GSDWAESPP--------------------------------EASPPCP------G  286
c_000000169465_2        234  -----KALPE-------KSTYM----MVAQ-----FNSQVYLEQRPST-GLWGGLYGFI---EVSSIEEGIE----------------------------QLAKRGIS------V  289
c_000003254110_11       234  -----KALPE-------KATFM----MVAQ-----FNSQVYLEQRPST-GLWGGLYGFI---EVSSIEEGIE----------------------------QFKKRGVN------V  289
c_000004750284_20       230  -----KVTPV-------RQTYM----LIPM-----FGQQVFLEQRPQS-GIWGGLYGFL---EAQSAEEGIS----------------------------LLAQRGIE------V  285
c_000000141782_15       235  -----REKPL-------RRTRM----LLIVD-----GGRVLLERRPPS-GIWGGLWCPP---ECPPEVDVES----------------------------YCRHELGLE------V  291
c_000002717847_8        224  -------KKD-------VYLEF----SLYK-----DGPKYFLAQTEAL-GFWKKLWMPP---VKIVN-----------------------------------------------  263
c_000004054799_1        215  ------QKPT--------VKLN----FILPH----TKNEILMHKKQAS-EYWESLWIPI---DGDEVKIK-------------------------------------------G  259
c_000006126673_1        241  -----KARPQ-------RYGYV----YWISD----DDGNILVHKRPEK-GLLGGLYGLP---TSSWETDIE--------------------TVEH----PIFISEQIK------P  301
c_000000339186_3        199  -----IRYED-------KQIDV----YLIT-----RGPRYLVQQRPEG-GVNAGFWEFP---NSDSGNKYS--------------------APK-------------------  247
c_000003029168_2        230  -----VPLQSREE-VGIVIWAG----KPYN-----ADSLIFIVQRPPT-GLWAGLWEFP---HDEQTTDEP----------------HNAAALR----IAEATVGPT------M  298
c_000001863436_1        246  -----ARQKP-------VAARTAVVTSGGA-----EGRRFLMVKRPAD-GLLGGLWEFP---TVEAAGDVA----------------PTAQAVS----RLLARLTGGP---VPA  315
c_000002830137_4        239  -----TQTEH-------VVEAA----VVIR-----RGSRVLLRRHEDR-ERWAGLWDFP---RFPFDPDATN--------------NGHQQLVD----KSRELTGFE------I  303
c_000004612302_3        232  -----VRTTD-------LYQLL----LVIR-----RDDRLLLIQHPER-ERWGGMWGFP---ALSRTGPLLKS-------------RIDELQIA----SEAAEWGCD------V  297
c_000001933926_1        217  -----VARPT-------RHGVV----FWATR----GDGCVLLRRRPEH-GLLGGMMEFP---STEWRECPW----------------RIRSAV--------KAAPFA------A  276
c_000003136334_1        229  -----KRKPT-------RYATV----FWLLD----GRGNVLLRRREEK-GLLGGMMEVP---STDWLEENT----------------PTKENS--------MFAAAA------V  288
c_000005849454_9        234  -----KKWVD-------LEMVF----LVHR-----VGVRVGLQKRTS--GWSPGLYEPP---SAICESFATANE-----------ASSAHESTAH----SLAREHR--------A  299
c_000001169194_1        234  -----VPARE-------ISFLL---VILQT-----EEGEVMLVQRPPE-GLLAGMWAFP---EQELAKPLD----------------CAATSRD---RAIELAMSLGA---EVV  301
c_000002786947_2        234  -----APARE-------IFFLL---VILQT-----EEGEVMLVRRPDK-GLLAGMWAFP---EQELHKPLD----------------CSTTSRD---RAIGLAMTLGA---EVV  301
c_000001279808_52       220  KKTTKRRKHR-------IEVGI----ACIW-----REGKYLVQARPKG-KSFEGSWEFP---GGKREKGES----------------FRGCVKR----EIEEEVGLN------V  287
c_000000358065_2        224  -----KKIIN-------KFYLA----TLYK-----HDDQVLLIKNDKF-KFLKNLLIFP---MKEISQS--------------------------------------------C  268
c_000003745941_1        218  -----KIKQE-------TIDRA----WVEQ-----GGKLLLHRANDKS-RRLSGILELP---TLDVMGY-----------------------SAT----ALMK-----------  268
c_000004187032_4        224  -----KKNKE-------KYFIL----KVYK-----KNQKYLLVKNTKF-NFLKNLAIFP---MEELSKP---------------------------------------------  267
c_000006067315_3        224  -----KFNKI-------KYFEA----NIYQ-----NQNKYLLIKNSKF-KFLNNLLIFP---MKEVEKN--------------------------------------------K  268
c_000005543774_1        186  -----IESKT-------YDIFC----YLRK-----NKKQIALTKNNDL-GFLKKFNLPE---IKATSKK--------------------------------------------N  230
c_000006211484_1        208  -----IQSKN-------YDIFC----YLQK-----NKKQIALTKNNDL-GFLKKFNLPK---IKETSEK--------------------------------------------N  252
c_000002718976_3        227  -----IKSKN-------YDIFC----YI-N-----TKKQIALTKNNQI-SFLKDFNIPE---IKE-TNNVSN-----------------------------------------N  272
c_000002992548_1        226  -----KKEQK-------YNVYC----YLKK-----KKKEIALTKNKHL-SFLSNFNIPE---TKIETSNNKK-----------------------------------------N  273
c_000000456751_1        192  -----IKEKK-------INIYC----YLNR-----YKKEIALTKNKNL-SFLNNFKMPE---IQMVVANNNLK----------------------------------------K  240
c_000002655634_1        223  -----TKEKK-------FNVYC----YLNK-----YKREIALAKNRNL-GFLYNFKMPE---IQMEVTNNNLK----------------------------------------R  271
c_000004232403_2        223  -----KKIPT-------RNLAA----ALIE-----YGDYILISKRPIN-GQLGGLWELP---NIELKNGKS----------------PEKSLKE----SINNHYGFT------I  285
6u7t.after.N146.pdb     226  -----TAVKQ-------VPLAV----AVLAD----DEGRVLIRKRDST-GLLANLWEFP---SCETDGADG----------------KEKLEQ----MVGEQ-----------  283
Consensus_aa:                .....................h.....hh.........phhl.pp....shh.shh.hP.....p...................................p...........


Conservation:                            6
sp_P17802_MUTY_ECOL     282  DNLTQLTAFRHTFSHFH---LDIVPMWLPV----SSFTG-------------------CMDEGNA---------LWYNLAQPPSVG--LAAPVERLLQQLR--TGAPV------  350
sp_P83847_MUTY_GEOS     295  ELTEPIVSFEHAFSHLV---WQLTVFPGRL----VHGG----------------------PVEEPY---------RLAPEDELKAYA--FPVSHQRVWREYK--EWASGVRRPD-  366
c_000003652391_2        287  DWRTLRGEVRHTFTHFH---LILRIRVAEL----PDDI-----------------------TPAVG---------FLLSKHAFRPSD--LPTV---------------------  338
c_000000031207_6        287  ELIGHADRFQHAITYRK---LFIHPLVYRY----QGE------------------------IPAGV---------ETIPIDGGDR----LPALHRKSIAAAR--ELLLEVFR---  352
c_000000430975_4        294  DWKPHPGRVYHSFTHFD---LELKIMTINS----EVT-------------------------PEGT---------FWCRKEEVKAQA--LPSLMHKIMTHMY--KA---------  354
c_000001007148_4        298  EFQEWMDEFQHGVTRFR---LHLQPIVAMW----RSGSW--------------------EGPVTA---------EWVGIDELATRP--LSVAGRRVATRVE--RILRHNGAQA-  370
c_000001092848_3        303  PWEPC-GTVTHTFTHFT---LALSVYRARV----ADMP-----------------------APAGS---------WWCAPGLLAGEA--LPTVMKKVVEAAI--SGATKARRPIG  373
c_000001151029_1        251  DWKPQPGLVYHSFTHFD---LELKI-----------------------------------------------------------------------------------------  272
c_000001797282_1        313  RWKRLPGLVRHGFTHFP---LELTVFVARV----ALATP----------------------APAGT---------RFTPRSALDDEP--LPGLMRKVLAHAF--DPKPEPEKKPR  385
c_000001803648_25       290  DWQDPGAEVRHSFTHFH---LRLSLRISVV-----GNA-----------------------KPTAG---------HFIADADFDPET--LPTVMRKAYRIAR--TKFDGDANG--  358
c_000002078955_4        290  DWQVLNAEVRHTFTHFH---LRLSLRVATI-----GNK-----------------------PPATG---------DFISKNAFDPET--LPTVMRKAYRLAC--TQFDDN-----  355
c_000002106160_4        287  TIKNKIGEIRHRYSHFG---ITLSGFHCRL----INGSK-------------------PEVNRPF---------RWIRLNEINQYA--FPKANHKLFNLMQ--E----------  351
c_000003607531_2        297  QWVKIAGTVNHAFTHFQ---LQLQVRVAVV----NDQT-----------------------QISGG---------IWVDRDRLDEFA--LPNLMKKVWAFAS--KRIQPLPRLDC  368
c_000003872363_4        286  DWQRLDAQVRHTFTHFH---LRLSLRIVVA-----KNV-----------------------IPTVG---------MFIPSDKFDPDD--LPTVMRKAHALAD--AAFDHG-----  351
c_000004820107_1        292  ESEQTNLSVKHSFTHFD---LIL-------------------------------------------------------------------------------------------  311
c_000005774797_1        276  SEWKGIGQIRHIFTHID---L---------------------------------------------------------------------------------------------  293
c_000005867021_3        290  DWHVLGAEVRHTFTHFH---LRLMVHVATV-----NEN-----------------------SPDVG---------KFIADDCFDPAD--LPTVMRKAHKLAL--GSFVGD-----  355
c_000004546210_2        287  LWVDSWEPFKHSFSHFD---LYIHPIQIRM----DGQFM------------------NTAYKSKT---------QSFSLNQLDSLG--LSAPVKGLVNKLY-------------  351
c_000004551008_5        287  LWVDPWEPFKHSFSHYD---LYIHPIQIRM----DGQFM------------------NTAHTPKT---------QSFSLNQLGSLG--LSAPVKGLVNQLY-------------  351
c_000002511148_4        282  LNIKKLSKVKHSFSHYN---LEAIPYLAKL----ESNK-----------------------KYKNS---------VWVDYKNVESLG--LPAPIKKTINQLT--KP---------  344
c_000000577378_2        270  LNIKKLGKVKHSFSHYN---LEAIPYLATL----GSNK-----------------------KYKNS---------VWVDYKNVESLG--LPAPVKKTINQIT--KP---------  332
c_000000598175_2        293  QESIALAEIKHTFSHFH---LYIQPLIINV----ETPIE-------------------LGVMEDNES---------LWYNITTEFNGG--LAAPVQTLINITK--NIEKEIENGTN  369
c_000000754627_3        287  SDLNEVAPFTHVFSHFE---LTINPHVLNI----EKAPD--------------------VVNDKQL---------VWYPLDQSIEVG--LAAPTKKLVKQIT--AIV--------  353
c_000000811118_1        274  PVEEDFSSFEHHLSHKK---LLISVKVLTI----KNKDR-------------------LSIDRRIF---------NWVNISDSVNMG--IPKPVKDILKKYD-------------  338
c_000000899687_2        290  VSLNKVMTIKHTYTQFR---VTLTVFNCKL----QKKQI------------------RPDGCEQW---------KWVSLSKLKKYP--FPAANVKIVKYLT--EK---------  356
c_000001176522_24       289  --GEALSAFRHTFTHFH---LDITPYLWHT----HSDD---------------------MPQQAG---------QWLPLAQALKMN--IPAAVKRVLQEVI--EYEQNR-----  354
c_000001345122_1        262  EQAVI----KHTFSHYH---LRMKPMM---------------------------------------------------------------------------------------  281
c_000001515736_6        279  RSWQDLPGFLHVLTHKD---LHLHPVLVPV----DQAYA------------------ALVSEADES---------CWADAAAWAELG--LPAPIRKLLDAEL--A----------  345
c_000001682161_6        283  DSVEILKQLKHTFSHYH---LMMNPVMLQI----ERE------------------------AIEEV---------SWFELENLNGVG--LPTPIKKILRESF--A----------  343
c_000001923643_29       289  ADWTELPAFLHVLTHRD---LHLHPVRVPV----HGSGP------------------DAAMADGQG---------CWADARAWADMG--LPAPVRKLLDAQF--AG---------  356
c_000002030994_5        282  SEADKLSKIKHTFSHFH---LHIQPLIINT----ETPLK-------------------MGVMEHDDS---------LWYNVTTEFNGG--LAAPVQTLLNITK--DIVKGTQNDTN  358
c_000002038721_4        276  EEKESLPLIVHNLSHQK---LSIFTKKYFL----KNKDR-------------------LPIDRRGF---------VWVNKKESVKMG--IPKPVKTILENHL-------------  340
c_000002523527_15       316  ARAYLAGELTHVFTHFR---LLIRAIRVDL----DTVAL----------------------EGDAAS---------RWLSLDDLDMLG--TPAPVRKLLEDQA--RFGLF------  383
c_000002608528_2        282  DEAIKLSEIKHTFSHFH---LHIHPLIINM----ETPLK-------------------IGVMEHDD----------------------------------------------------  321
c_000002961510_2        290  ISLNKVMTIKHTYTQFR---VTLNVFNCEL----QKKQI------------------RPDGCEQW---------KWVSLSSLKKYP--FPAANVKIVKYLT--EK---------  356
c_000002979152_8        290  PSLQAYAEIEHEFSHFH---LSAKVLRIEL----DSDKR-------------------LGVMEETNA---------LWYNMHKDNPGG--FPAPIKKLLSRNL--L----------  356
c_000003248034_1        302  KHPKKLCKFRHSITFRL---YDVSIYTAIL----EGE-------------------------LPEPF---------CWVPQRKLATIP--LTTVTRKALAVLS--PNSSDRNDEQN  372
c_000003294679_3        286  LKISRWKTLQHKFSHFD---LKIHLFKAEP----DIAIK------------------NIANNDSA---------LWYKSDSQTRIG--LAAPVKKLLETQF--NIQ--------  353
c_000003333364_1        288  AASETIAVIRHGYTSFR---VTLTAFLLRF----GEPPEGW--------------PEPVLHDATEG---------VWVSFAELDRYT--FPAGHRKLIDQMS--RDMRYAWLV--  366
c_000003402697_5        288  SVRPHFYEELHEFERTE---LLLRFHRCQI----QAGEP------------------SPQEEQEI---------KWVSPDDFGDID--FLKTNHKALDKLK--EMRV-------  356
c_000003492925_4        253  CASEYIGEVTHAYTHFK---LICKVYVYDE-------------------------------LDAEQN---------SYFTSSKIQKLA--ISKVDDKILKLYL--DTIQTY-----  316
c_000003766664_5        302  RIEGQCSSVRHVLTHRD---MDIAVFALSV----KGREPS-----------------PALSTYTGG--------MWIHSPEALEDLG--VGRVTRKILEAAA--THEISGQER--  378
c_000003948186_72       284  DSCTELAPRRHTFSHFH---LLITPLHIKS----SQQTT------------------HVMEDQQQ---------LWYNMRKPASVG--LAAPVKRLLRQTS--NQ---------  350
c_000005057120_4        282  QSVQHIEKQRHTFSHFH---LDYTAVLVKT----ENRIN------------------NVMESNQS---------VWYKSDKINALG--LPAPIKKLLQNQN--RNEV-------  350
c_000005590109_5        282  KLIKQLDEQRHTFSHYH---LDYTGIMVKI----ENPIN------------------IVMEANQS---------VWYKSTQLKFLG--LPTPIKKLLQNQY-------------  346
c_000006063368_2        282  PSLIQIKKVKHSFSHFN---LEAIPYLVKI----KEKK-----------------------SIKNT---------VWVDSKNIESLG--IPSPVKKTIKEIR--VP---------  344
c_000000467631_2        261  TSKKQLDGQRHTFSHYH---LDYTPIEVKI----ENPIN------------------NVMEANQS---------VWYKSEQINSLG--LATPIKRLLQNQ-------------  324
c_000000788235_1        283  PSATTESHFSHTFSHFR---LQAQTLLITQ----STPIK------------------RVMEGTPS---------LWYNSNTTFMGG--FPAPIKTIINKLN--EDF--------  350
c_000001187176_11       283  AEPEALSPLRHTFSHFH---LDIQPLLVRV----DAPDR--------------------VTESGQ---------VWYNLREPSRLG--LAAPVKKLLQRAE--LNTQADERTSP  356
c_000001660697_64       297  SDVEAAGALTHTFTHFR---LHMHLLHADI----TKP--------------------ATLDDDW---------RWVPLAQLNSVG--LPAPVKLALETLV--QPSLL------  363
c_000003378864_2        301  EVGPLVTQVRHAYSHFKITLHAYHCRLLRG------TPH-------------------PKVAVES---------RWVPLNGLDQFA--FPKANKRVLEVLEQPPAMPEVTP---  375
c_000003910742_3        284  ENTEKETKLSHTFSHFR---LHIHALLIRQ----TPPIK------------------RVMEGTPS---------LWYNRSTKFTGG--FPAPIEKLFTRIL--T----------  349
c_000004116181_2        249  KTSEVLPTLRHTFSHFH---LQYTTLVVRT----DNPLN------------------FVMEVDQA---------VWYKAEQIPGLG--LPAPVKRLLDNLI--LNEDNND----  320
c_000006223903_1        284  LFESAWSVLRHSFTHFE---LAITPQPARA----PKRLT------------------RRMQSTGL---------LWYKPGEPF-----------------------------  332
c_000001029068_2        257  QVRKKLPLINHSFTHYY---LTI-------------------------------------------------------------------------------------------  276
c_000001128125_11       291  ESLVEWNYFRHTFTHYH---LDIYPLVYRLD--EGEVSPVFTIG--------KSQNNDEKASAEM---------NWIELDRLNKGEVGVPAPVNKMLERLS--ETLDK------  374
```

```
c_000001595844_4      288  NIKNKLGPVTQLLTHQK---VIGQFFDITL----VGE-----------------------KDLDI---------APISKVDLQVFP--FPKMMMMYINRIM--A----------  348
c_000001719155_2      292  HIDEKLMTVKHSYTRFR---VILHVFLCRV----LSGRV-----------------SPTHCEEW---------DWVKTEELDRYP--FPAANVKIIKSLR--KNQGRS-----  362
c_000002363038_6      293  HSLSEGEPFRHSFSHYH---LDIFPLIF-------QASTDYEIGQP---SNRVQETPAEYSVAHH---------QWFDINRLKSGEIGVPVPIRKMISSL--------------  370
c_000002561400_1      247  EGLIEWDDFRHTFSHYH---LDIYPLIFDE----RGAPKNTAKI--------ISEPGGVYDAEQG---------SWFDIQLLLDGGLAVPAPVNKLIGRLD--ELVE-------  327
c_000002566035_2      284  KQQKEYKPIKHAFTHFH---LTIRPIHIKI----RIYKD------------------KDYDTKVL---------KWIHPKTNPNLG--LPAPVLSMLIDLN--NTKRAVLNES-  358
c_000002598045_3      282  SNIKKLKKVKHNFSHFN---LEAIPYLVKV----KKGK----------------------KIKNT---------VWVNFKNVESLG--LPAPVKKTINQIT--MP---------  344
c_000004188575_41     280  TVSKQPAVFRHIFTHFK---LDIHPHLYHA----KQSAA-------------------ENITGQA---------IWYKIEDALTLG--LPAPVKKTLLTLS--KG---------  345
c_000000240049_1      282  NSHNDLAAFRHTFSHFH---LDITPCEVDI----TQSAY------------------AIAENDRF---------QWH--ADITQLA--LAAPISAIFRENN--SLK--------  347
c_000002139456_2      282  NSHNDLAAFRHTFSHFH---LEITPCEVDV----AQSAH------------------AIAENDRF---------QWH--ADITQLA--LAAPISAIFRENN--SLK--------  347
c_000003535614_2      284  KKHHDLPVFRHTFSHFH---LDITPCEIEI----KSISN------------------AIGDDSCY---------QWQ--VDISKLA--LAAPVSAIFNAK--------------  345
c_000002657784_4      281  KDLKTYPDINHSFSHLN---LNAKLFLLKL----NEDIN------------------YNINKGKY---------VWVDKKGLKALG--IPTPVKNIFEVLK--LNDQTS-----  351
c_000003800129_15     263  ---NNNREIKHGLSHRE---LHLEFLVTSG------------------------------TPKGG---------KWINKNEFNNLP--TPKPISDKLEQYA--KSFL-------  320
c_000003716781_1      273  LQREEVGELRHSITKHR---IRARVLRAVP----GRDE----------------------LPEGW---------AWFERSEIEALG--VTGMTSKALTLGR-----ARSR----  337
c_000001556689_3      294  RLGPKLGEIRHGMTDRR---ISCHVYEASL----SGSQTTMAGA-------PTGLEKSDHNIPDS---------GWFTIEEVMTLA--LAASARKTLTTLL--DIDLTTAHNPG  380
c_000001834452_1      254  KLGEMMGSARHTITRHR---IQARVHQVEG----------------------------------W---------NGKGAVDPSSVP--LSSLGRKVLSIAG--VLGGLLLLSPD  317
c_000004255004_2      275  QQGDPVALIHHAITDMR---ITAPVYLLQC----KIPV----------------------KGPEW---------RWVSLANLHAYP--LSSLSTKALSAAR--QHLPTTRKLGG  346
c_000000605438_3      263  ENMTGLGEFKQHYTHFT---LDARVVLLKD-------------------------------KKQE---------NYFTVEEIHDLA--LSGADRKALALLE--KYLQ-------  322
c_000000581237_15     263  KDMIKLGEFKQHYTHFT---LEASVVLVQN-------------------------------KEQE---------RYFRINEIHALA--LSGADRKGLALIE--KHLA-------  322
c_000002529579_2      263  NNMTELGEFKQHYTHFT---LDGSVVLVTN-------------------------------KKQK---------NYFTIDEIHDLA--LSGADRKALALLE--KYLQ-------  322
c_000006097838_1      268  VEPESLCIINHSITCYR---NRLEMFVIFR----TRKPL--------------------LAKLKG---------KWVPLAKLDEIP--LTTAHRKAANQFA--G----------  331
c_000000583727_6      294  AKFQPLCNINHSITRYR---NRLEMIVAEP----KRKPR--------------------LAKLKG---------EWLPLAKLAELP--LTAAHRKASDYFL--KTANSR-----  362
c_000005807640_2      295  AELEPLCTINHSITRYR---NRLEMFVIEP----ARKPR--------------------LAKLNG---------EWIPLAKLDELP--FTTSHHKAANQFC-------------  357
c_000000134878_1      270  KPETIIHQVDHHYSHFS---VTINAINCKY----NGGDI------------------KLSGPTDF---------KWIHTSKLHSYA--F-------------------------  322
c_000005136725_3      285  KPEKLIHQVDHHYSHFS---VTINAINCYY----NGGKI------------------KLSGPTDF---------RWVKTSRLHSYA--FPKASIKLFNAIK--SIE--------  352
c_000001684786_4      280  ETGEELKAIKHSITFRR---LTLRPRLLET----GSFTI--------------------RGKPSF---------RWALPEELGSVL-PVSSLCLKIADRLS-------------  343
c_000002971826_23     290  EEGAELEAIRHSITFRR---LTLRPRILGA----GSFST--------------------RAKPSF---------RWGRADELGNVL-PVSSLCLKIADRLS-------------  353
c_000002040695_2      291  ASWTLLGQIQHVFTHFT---LKLDLWYTIR-----------------------------TVKTQG---------QWVLLNQMNDYP--LSSLTKKSLAFLV--KNLELEKEQAR  359
c_000003263657_32     303  KTDLVLPTVLHVFTHFK---ITLYGFGCTF----IKGAA------------------KALTCHDA---------RWVSQTQLADFA--FSRAHNRLIDIMK--KEAAKGQVEMF  378
c_000005989041_2      297  QWVKITGTVTQPFTHFE---LQLQVRVAVV----ENKP----------------------KSSEG---------IWVDRNRLDDFA--LPNIMKKVLAFAL--RHNQHFSREFF  368
c_000002391082_2      298  EVGARLTSVDHAYSHFS---ITLHALEARY----LRGEP------------------QAIGCADW---------KWIVPDELDDYA--LPRADRLIVEFLR--QDGRQLGLFDS  373
c_000003839553_2      305  EAVARLTSVDHAFSHFS---ITLHAIAARY------------------------------------------------------------------------------------  331
c_000003996707_2      295  EVGGLLAKVDHGFSHFS---ITLNLFEAMY----RGGEP------------------EAVECDDW---------RWIDPAELDDLP--MPRADRRALEELS-EEEGQRVPVLMA  371
c_000002044706_1      271  ----LVVTSEHRFSHRH---WTTRVYRA------RVEDE----------------CSGPADASHWSEGL-----RWTCAGDLGKLA--VPEASRKTLRPCLRASRGSLPG----  353
c_000006007075_1      268  --KNLQKTLNFKMSNMN---MNIVIKIMKI----SSN------------------------ISDS---------SWIKETKFNHYT--FPTFTKKIFNSLE--NYL--------  327
c_000002094036_4      245  SLLKSKNKLNLKMSNMN---MNICIDFVKI----KKK------------------------PKNG---------LWIESSKLENCM--IPTFTKKIFASVK--NNL--------  306
c_000002762689_1      268  --LSSTDIVNFKMSNMN---MNIKIEHKNK----VGS------------------------LPTS---------HWIDPKKLKNYT--LPTFTKKIVRYLE--SNK--------  327
c_000005516980_2      268  --KNFNENLNFKMSNMN---MNIKIQYSKN----NIK------------------------LPSS---------YWVDQKKLNNYM--LPTFTKKVVKYLE--NNK--------  327
c_000003920004_1      293  KKFTKCKPILYSFSHFD-------------------------------------------------------------------------------------------------  309
c_000004008511_2      287  NKLKLKGFVDYSFSHFD---LETEVFFTKV----KKDY-----------------------FANQ---------KWIKKNNIKISG--LPTVMKKIIEVAL-------------  346
c_000004481347_1      294  VKYKRIGKITYSFSHFD---LDIGIYKSKV----VKKK-----------------------FKNF---------NWIKSNKIDTSG--MPTVMKEIVKKSL--KI---------  355
c_000003787733_3      289  NIGKKIGHVKHAYSHFR---ITLTLYQCKT----NRSVK--------------------NIYKEY---------KWIIPTEVNNYP--FPKSNHKLFK----------------  348
c_000000754657_2      289  NIKKKVGNVKHAYSHFK---ITLTLYRCQS----QGSVK--------------------SNNKEY---------RWITPAEIKYFP--FPKSNHKLFDVLN--SNGWNV-----  357
c_000000990943_3      289  NIEKEVGYVKHAYSHFK---ITQTLFQCQT----QESVK--------------------SINKEY---------RWITPIEVNNYP--FPKSNHKLFTILN--SDGWNV-----  357
c_000004474996_2      288  NIKNKVGYVKHAYSHFK---ITLTLYQCQT----QESVK--------------------IMNKEY---------RWITPSEIQYFP--FSKSNHKLFNLLN--SNGWNV-----  356
c_000001463500_11     276  QIQNKICTIKHSYSHFD---IRVSLYHCRI----NDINI--------------------KNMKNV---------CWIRPKDINNYA--FSKSNHKLFKILN--TNGWTI-----  344
c_000001286181_5      263  QNINELPSFKHSLSHKD---FHISPRIINI----PDDME--------------------TDDKKT---------IWIEKNKIAKLG--APKPVLDIVKKIL--NQNDKGLL---  333
c_000001293628_3      302  VRFSPLPEVRHTFTHLK---AVYRPMLVIG----TRCEG-------------------SNPDSEH---------KWVLPDQVEDLP--LPVAQRRILDLAW--AALEGC-----  371
c_000001535696_8      292  VLRYNHKIIVHKLSHQQ---LHTHFWIVVI----SNELD-----------------------------------GSILLSEIENYP--VPVLIANFINTFK--NSYFWPNNSIR  359
c_000001614067_2      298  KWKILDQNLNHIFTHFK---LNCSLAIATI----NDENAL----------------VNDLSKSSY---------RFVQKTNINELA--LPSLIKKILNALK--NNEILDF----  371
c_000001765289_1      262  SIKKRIGAVDHAYSHFS---ITFHGYHCIE----NGEK----------------------------------------------------------------------------  292
c_000001961666_1      274  NRILSKNVVRHEFSHFK---LELEVYYIET----RFP-----------------------QITHG---------EWLKTKQAGKQ---LPSVMKKVLDNI--------------  331
c_000002018097_4      284  DILNCFDKVKHSYSHYK---LLVTPFECEV----LNA-----------------------DSRIG---------RWIRVGEIDKYA--FSKVNHKLFNAIG--IKNY-------  347
c_000002843512_36     254  CASKYIGEVTHAYTHFK---LIIQVYVYDE-----------------------------LDAEKN---------SYFSSQEIQKLA--ISKVDDKILNLFL--NSIECD-----  317
c_000002930199_1      281  SNIKNKYTIEHNLSHQK---LKISFWDVDA----KNIN----------------------TQKGF---------EKITLDNISNYP--FAKPLEKYFQTQH--LIT--------  344
c_000003033795_2      277  SNPIFLGNITHKYSHFG---VNISFFISFP----KSIKS-------------------FNSSQEY---------RWIRIKNILNYP--LPKANHKMLDILK--KL---------  342
c_000003888107_2      278  KNPNYLGQIKHQYSHFK---VLISLFLADV----NDISS-------------------LKPNQNY---------KWTTRKQLDKFA--LPKANYKMLEILD--KLN--------  344
c_000004013286_6      307  QIGNKIGQIRYVVTRFR---TTVDCYEAHF----SNIGKPNRSKNDISVNTLQKEQQEAGSLWHM---------QWATRKQIDSFP--MSVPARRVIRLLA--DFD--------  392
c_000004852258_1      265  KVGHKLGMINHTFTHFK---MNVLLYNCQA----KDQP---------------------LSKNTE---------KWVSFSDLDQYA--FSKANHKLFKLVE--LRND-------  330
c_000005254087_1      300  RTDRFLTSIKHAYTHFR---ITLHAFHATH----LRGEP------------------QHIGVADH---------AWVTLADLDKYA----------------------------  351
c_000005603677_1      290  VWALISGQEQQQLSHQK---IIARFLWPKQ----PLPQ---------------------ALSADW---------ELVDAQSWPNYP--LPRLIAQYWEKKQ--LAFF-------  355
c_000003283462_3      276  VPKKKIESIKHKYSHFS---ITLTAFHCKI----KNGAK-------------------VPSKQST---------RWITPGEIGDYP--FPRANHKLFNQLE--NFYSNG-----  345
c_000002687221_1      290  APVAFVKQIKHAYSHFS---ITVDAYSCTF----RGGRP------------------RALGCADY---------RWIYPHETGKYA--FHSAGHKLFDKIL--ETAS-------  358
c_000003347358_19     285  CASEYIGKVTHAYTHFK---LICKVYVYYE-----------------------------NHPEEA---------DYFSEEEIQKIA--ISKVDEKIVNLYG--NFLKYD-----  348
c_000002701031_2      286  QILKISATIKHQLTHQT---IFARFWHIDT----KIQ-----------------------KVENS---------KYISKKELKNYP--ISRLLEKYLENIG--MI---------  347
c_000000582753_3      292  AIRKVHLKIKHVLSHQN---IYAKLIEVEV----LDVI----------------------RLENY---------IKVEKEDFESYP--IHRLMEKMMEQVG--K----------  353
c_000001713769_5      288  IILFNSSPIIHKLSHKT---IYAKFWILPV----NGSNS-----------------------------------NSIKFSDVNKYP--VPRLIEKFLDKFN--YKHF-------  348
c_000004369364_1      288  KIKHKSAPSKQLLTHQK---IIGIF-----------------------------------------------------------------------------------------  309
c_000006057486_30     292  ISKYNQKQIVHKLTHQH---LFTTFWIINT----NTCSK-----------------------------------NTVNWQNLSNFA--LPTLIQNFVDDFK--K----------  349
c_000005494072_10     290  VSLFNPKPIIHKLSHQH---IYTSFWIVNY----NGSVS-----------------------------------NTISIDELKKYP--VPVLIDNFIKDFE--M----------  347
c_000001742634_3      293  PYLFNEKTVIHKLSHQH---IYTKFWIVKL----DVNLE-----------------------------------QTIPLNSIEKYP--VSTLIQNFIKEFN--F----------  350
c_000002826998_2      290  QIKKISLPFRQNLTHRK---IIATFFEIDF----MDKFS--------------------NKNNNY---------IKIERKNLSKFA--FPKIIDLYLKDNS--LNLAMK-----  358
c_000003159439_6      289  TSLYSDNIKAHKLSHQH---IFSKFWIVET----KQNIL-----------------------------------NSILIEDLEKYP--VPKLIQNFIEKFD--NIKLNPATK--  354
c_000004887214_1      294  LQLLTPKVVVHKLSHQH---LYIKFWEIRV----PTFRS-----------------------------------ATISWQELLKLP--FPIVIFKFIREFL--ETSMPNFDTPL  361
c_000005037037_2      288  KVKKRSKNFTQRLTHQK---LNAIFIEIDL----KTK-----------------------INKQF---------INTDINNLSKFA--FP------------------------  336
c_000001059964_1      280  QLGKEIHTLEHRYTSFR---TRVSFFTATV----HVLPE--------------------SSQKRV---------RFVKTSEVE-------------------------------  326
c_000002498472_1      289  EIVEKITNTVHHYTRYK---VTLHCFLLRL----KKGSDT---------------DPVLHAAQDF---------NWVPFKALQEYA--FPAGHRKLITYL--------------  355
c_000004615912_3      285  QTKK----YQQELTHQK---ISAYFWEIN-----LNVAP--------------------VNIYDF---------ILIDSKNLAKFA--FPKIIALYLENNS--LNL--------  345
c_000004766858_2      292  RIGPELFKMEHDYTRFH---ATLHCRLCTV----AEIPS--------------------VLPDNA---------QWLTLGEISALA--LPRVFQRLRKRLL--DEVSA------  359
c_000002747260_18     286  PWRDPGAEVRHTFTHFH---LRLALRVADL----PASA----------------------TPRTG---------QFIPHHAFRPGQ--LPTVMRKAYDLAS--ATFLGN-----  352
c_000005371561_38     287  NWRTVPGEVRHTFTHFH---LVLELRRADL----PEDC----------------------TTMRG---------QFLAPGGFRPSD--LPTAMRKAFDLAR--DG---------  349
c_000000169465_2      290  DETRTLEGFRHTFSHFH---LDITPVVAVV----NSAPS------------------KRVAETAF---------RWFSLNEPIEVG--LAAPTTKIIQQLM--R----------  355
c_000003254110_11     290  EETKTLETFRHTFSHFH---LDITPVVAVV----NSPPT------------------KRVADTAF---------RWFSLGEPIEVG--LAAPTTKIIKQLI--G----------  355
c_000004750284_20     286  EEIQQLDEFRHTFSHFH---LDITPLIAVV----NSTPQ------------------KRVAENES---------RWFLLDEPIEVG--LAAPTTKIIKTLA--K----------  351
c_000000141782_15     292  TRPQAWPGLRHTFTHFQ---LDIQPVRLRL----TAAPA------------------RLMDGDRH---------VWYNVESPDARG--LAAPVARLLQGLA--LEDTRA-----  362
c_000002717847_8      264  ---KFNAANKHALSHRH---IHFRFKQIAN----LPT------------------------GMKG---------EWFSKKDLANIA--LPKPISDKLLQDG-------------  319
c_000004054799_1      260  NQKYSKIKVNHPLSHLN---LDMKIKTFKI----DKKCD-------------------------------------------LDSN--L-------------------------  296
c_000006126673_1      302  KPYKQLPFIMHVFTHFS---LKLYLCKTSE----FICDN---------------------VPEEH---------KWVFISTLEDIG--FPSVFEKVKKII--------------  362
c_000000339186_3      248  -NGQPFASARHSITRYR---IQLNAHLIHA------------------------------KRGKG---------MWCTSDEIRSLP--FSSAHLKLLGKLP--HRLDK------  308
c_000003029168_2      299  QKLTKLVTITYTVMRTR---TTLHVFEGML----DLETS------------------TMQEASSR---------LWVRLDGLHKHP--FSSPQHEIIKTLD--KEAALQLAGLV  374
c_000001863436_1      316  TPRVELGEVVHHFSHIR---MTITAEHRVA----SSEQVRL--------------RSDDEVGQEA---------RWVSAVELVDLP--MSAAMRKIEGLYR--ELGSGAI----  391
c_000002830137_4      304  EPVRQLTTIQHGVTRFQ---ITLQCHLAQC----VSGRK---------------------RGPHL---------SWRMPHELEHLP--LSVTARKIGRLVA--KLAD-------  369
c_000004612302_3      298  EVEEWLQEFRHGVTRFR---LHLQPVVASW----RAGHW--------------------IGPVVA---------EWVEIDELTARP--LSVAARRVADGVQ--AGQRCGGASG-  370
c_000001933926_1      277  AWRELPGAVRHSFTHFH---LELQIMTVET----NEEVEG--------------------------------------------------------------------------  309
c_000003136334_1      289  DWQMLSGKVRHTFTHFH---LEL-------------------------------------------------------------------------------------------  308
c_000005849454_9      300  KLGAYQGQVRHTITHHR---IRAHVYAAEG----------------------------------Q---------APTHLEDPTQVP--LTGLARKVLDRIT--SA---------  354
c_000001169194_1      302  GVASALAEVQHRFTHLQ---ARYRPWVVPV----AKL----------------------LNGEGN---------VWMTPGEPVDFP--IPVAQHKVLDALA--ECGATLGSSES  373
c_000002786947_2      302  GHASALAEIQHRFTHLQ---ARYRPWVVPV----AKL----------------------LTGEGS---------MWMTPGEPVDFP--IPAAQHKVLDALA--ECRATPDSLES  373
c_000001279808_52     288  SVRSHFYEELHEFERTE---LLLRFHRCQI----QAGEP------------------QALENQEL---------KWVSPDEFDSVG--FLKTNHKALEKLK--SMRV-------  356
c_000000358065_2      269  SLSKPDHKLNLKMSNMN---MNISIDFSNN----KKK------------------------LKNG---------LWVEKTKLENHM--IPTFTKKIFASVK--HNL--------  330
c_000003745941_1      269  RGNTPIVTRKRGISNSQ---ITEPIWRLSD----NSEA----------------------DHAEM---------EWIDLEQIKKVT--LSGPHKRWINEIL--TDFNKQK----  336
c_000004187032_4      268  --KNFNENFNFKMSNMN---MNIKIQYLKN----LKK------------------------FPSS---------YWFDKRRLDNYM--LPTFTKKVVKYLE--KN---------  326
c_000006067315_3      269  FNTSLNKKINIKMSNMD---MKIILNKKNK----LIK------------------------IKNS---------YLVDKKNIKDLI--LPSFTKKIFNSVS--NYL--------  330
c_000005543774_1      231  KNWKFLCNYKNSISNKK---LNLNLYYKFS----SRI------------------------PSKY---------NWYSL-KKNKEF--IPTFTKKIFKQIT--HLY--------  291
c_000006211484_1      253  KNWRFLCNYKNSISNKK---LNINLYYKFT----SKI------------------------PSEY---------NWYSL-NKNNEF--IPSFTK--------------------  303
c_000002718976_3      273  QNWIFLKNYKNSISNLK---LNINLYYKFS----KKI------------------------PRSF---------NWYSL-KENKEF--IPSFTKRIFRQVS--TLF--------  333
c_000002992548_1      274  NGWIYLCNYKNNISNIK---MNINLFYKFI----KNK------------------------PKKY---------MWYPIDRRSNEF--IPSFTK--------------------  325
c_000000456751_1      241  RGWYYLCSYKNNISNIK---MNIDLFYKFT----KKI------------------------PKKF---------NWYSIDRSNQEF--IPSFTKKIFKKIA--KVYS-------  303
c_000002655634_1      272  REWNYLCNYKNNISNIK---MNINLFYKFT----KNK------------------------PKKF---------DWYSVDKSSQEF--MPSFTKKII-----------------  326
c_000004232403_2      286  EVGKNIGLIQHTFTHFK---MNITLFCCLL----KYDQ---------------------KIDSSA---------KWVRLFELDQYA--FSRGNHKLFQLYQ--KENV-------  351
6u7t.after.N146.pdb   284  ELTEPIVSFEHAFSHLV---WQLTVFPGRL----VHGG---------------------PVEEPY---------RLAPEDELKAYA--FPVSHQRVWREYK--EWAS-------  349
Consensus_aa:              ........hp+.ho+bp...l.hp.h.h...............................................@h..pph.phs..hs...p+hhp.h..............
```
