# Supplementary material for: Metagenome mining and functional analysis reveal oxidized guanine DNA repair at the Lost City Hydrothermal Field
Source: PLoS One. 2024 May 8;19(5):e0284642. doi: 10.1371/journal.pone.0284642 (PMC11078426; doi:10.1371/journal.pone.0284642)
Supplement: S1 Table — We were interested in determining how similar the amino sequences of LCHF MutY representatives were to existing MutY enzymes. We visualized this in the form of a percent identity matrix that was generated by Clustal Omega [86]. (PDF) [file pone.0284642.s005.pdf]

S2 Table. Percent identity matrix.

	Gs MutY	LCHF MutY 4	LCHF MutY 1	LCHF MutY 2	*Ec* MutY	LCHF MutY 3
Gs MutY	100	37.5	31.2	31.2	38.0	33.3
LCHF MutY 4	37.5	100	34.4	33.4	33.3	33.7
LCHF MutY 1	31.2	34.4	100	65.5	37.0	34.8
LCHF MutY 2	31.2	33.4	65.5	100	39.9	34.8
Ec MutY	38.0	33.3	37.0	39.9	100	48.3
LCHF MutY 3	33.3	33.7	34.8	34.8	48.3	100

We were interested in determining how similar the amino sequences of LCHF MutY representatives were to existing MutY enzymes. We visualized this in the form of a percent identity matrix that was generated by *Clustal Omega* [86].
