# Supplementary material for: Metagenome mining and functional analysis reveal oxidized guanine DNA repair at the Lost City Hydrothermal Field
Source: PLoS One. 2024 May 8;19(5):e0284642. doi: 10.1371/journal.pone.0284642 (PMC11078426; doi:10.1371/journal.pone.0284642)
Supplement: S2 Table — a Marinosulfonomonas MutY contig belongs to two separate MAGs and each are reported separately as MAG 1 and MAG 2, respectively. b KEGG ID gene not identified in any MAG and not reported in Table 2. c Completeness and contamination scores generated by CheckM v1.0.5 as described in Brazelton et al 2022 [35]. A KEGG ID analysis was used to identify the potential metabolic strategies of the MutY encoding organisms at the LCHF. The full metabolic KEGG ID search is shown above. (PDF) [file pone.0284642.s006.pdf]

S4 Table. Metabolic gene identification.

Metabolism	Gene	KEGG id	*Marinosulfonomonas* [a] MAG 1	*Marinosulfonomonas* [a] MAG 2	*Rhodobacteraceae*	*Thiotrichaceae*	*Flavobacteriaceae*
Cytochrome Oxidases	UQCRFS1	K00411	X	X	X	X	
	coxA	K02274			X	X	
	ccoN	K00404	X	X	X		
	cydA	K00425		X			
	cyoB	K02298			X		
Aerobic CODH	coxS[b]	K03518					
	coxM[b]	K03519					
	coxL[b]	K03520					
Methanogenesis	mcrA[b]	K00399					
	mcrB[b]	K00401					
	mcrG[b]	K00402					
Methane Oxidation or Nitrification	pmoA[b]	K10944					
	pmoB[b]	K10945					
	pmoC[b]	K10946					
Sulfur Oxidation	soxA	K17222	X	X	X		
	soxX	K17223	X	X	X		
	soxB	K17224	X	X	X		
	soxC	K17225	X	X	X		
	soxY	K17226	X		X		
	soxZ	K17227	X		X		
General Nitrogen Metabolism	narG	K00370	X	X	X		
	narH	K00371	X	X	X		
Dissimilatory Nitrate Reduction	nirB	K00362		X	X	X	
	nirD	K00363		X		X	
	nrfA[b]	K03385					
Denitrification	nirK	K00368			X		
	norB	K04561			X		
	norC	K02305			X		
	nosZ	K00376			X		
MAG Completeness (%) [c]			88.4	88.2	93.7	66.1	44.3
MAG Contamination (%) [c]			16.4	0.6	1.4	11.8	1.6

[a] *Marinosulfonomonas* MutY contig belongs to two separate MAGs and each are reported separately as MAG 1 and MAG 2, respectively.
[b] KEGG ID gene not identified in any MAG and not reported in Table 2.
[c] Completeness and contamination scores generated by CheckM v1.0.5 as described in Brazelton et al 2022 [35].

A KEGG ID analysis was used to identify the potential metabolic strategies of the MutY encoding organisms at the LCHF. The full metabolic KEGG ID search is shown above.
