# Supplementary material for: Metagenome mining and functional analysis reveal oxidized guanine DNA repair at the Lost City Hydrothermal Field
Source: PLoS One. 2024 May 8;19(5):e0284642. doi: 10.1371/journal.pone.0284642 (PMC11078426; doi:10.1371/journal.pone.0284642)
Supplement: S3 Table — * Binding affinities are reported for the binding modes generated by AutoDock VINA. Each mode represents a predicted ligand pose, which differs by a combination of position, orientation, and rotamer conformation. The receptor structure was obtained from PDB ID 6u7t for Gs MutY and through structure prediction for the LCHF Marinosulfonomonas MutY, Rhodobacteraceae MutY, Thiotrichaceae MutY, and Flavobacteriaceae MutY. The binding mode representing the starting complex for molecular dynamics analysis is highlighted. (PDF) [file pone.0284642.s007.pdf]

S6 Table. Ligand binding affinity (kcal / mol) *.

**Adenosine**	*Gs* MutY (6u7t)	*Marinosulfono-monas* MutY	*Rhodobac-teraceae* MutY	*Thiotrich-aceae* MutY	*Flavobac-teriaceae* MutY
Mode 1	-7.3	-6.8	-6.8	-7.0	-7.2
Mode 2	-6.9	-6.3	-6.5	-6.9	-7.1
Mode 3	-6.8	-6.2	-6.3	-6.3	-7.0
Mode 4	-6.7	-6.2	-6.3	-6.3	-6.9
Mode 5	-6.7	-6.2	-6.1	-6.3	-6.8
Mode 6	-6.7	-6.0	-6.0	-6.3	-6.6
Mode 7	-6.6	-5.9	-6.0	-6.2	6.5
Mode 8	-6.5	-5.8	-6.0	-6.2	-6.4
Mode 9	-6.5	-5.7	-5.8	-6.1	-6.3

**OG**	*Gs* MutY (6u7t)	*Marinosulfono-monas* MutY	*Rhodobac-teraceae* MutY	*Thiotrich-aceae* MutY	*Flavobac-teriaceae* MutY
Mode 1	-7.7	-7.5	-7.7	-8.0	-8.0
Mode 2	-6.9	-6.8	-7.6	-7.5	-7.5
Mode 3	-6.9	-6.8	-7.5	-7.3	-7.4
Mode 4	-6.8	-6.8	-7.3	-7.0	-7.3
Mode 5	-6.7	-6.7	-7.1	-7.0	-7.2
Mode 6	-6.6	-6.7	-7.1	-6.8	-7.2
Mode 7	-6.5	-6.4	-7.1	-6.8	-7.2
Mode 8	-6.5	-6.2	-7.0	-6.6	-7.1
Mode 9	-6.3	-6.2	-6.9	-6.6	-7.0

* Binding affinities are reported for the binding modes generated by *AutoDock VINA*. Each mode represents a predicted ligand pose, which differs by a combination of position, orientation, and rotamer conformation. The receptor structure was obtained from PDB ID 6u7t for *Gs* MutY and through structure prediction for the LCHF *Marinosulfonomonas* MutY, *Rhodobacteraceae* MutY, *Thiotrichaceae* MutY, and *Flavobacteriaceae* MutY. The binding mode representing the starting complex for molecular dynamics analysis is highlighted.
