# Supplementary material for: Metagenome mining and functional analysis reveal oxidized guanine DNA repair at the Lost City Hydrothermal Field
Source: PLoS One. 2024 May 8;19(5):e0284642. doi: 10.1371/journal.pone.0284642 (PMC11078426; doi:10.1371/journal.pone.0284642)
Supplement: S4 Table — a Confidence intervals (95%) determined by a bootstrap method, see Materials and methods for details. b Mutation frequency reported as median number of resistant colonies per 108 viable colonies. Fold change was calculated by dividing RifR frequency by the frequency measured for cultures expressing Ec MutY. (PDF) [file pone.0284642.s008.pdf]

**S9 Table. Rifampicin resistance assay.**

| MutY Expressed | Median (ci.low[a], ci.high[a]) | Mutation Frequency[b] (per $10^8$ cells) | Fold Change of EcMutY (ci.low[a], ci.high[a]) | n |
|---|---|---|---|---|
| *null* | 101 (81, 145) | 20.26 | 8.78 (6.44, 13.3) | 90 |
| *E. coli* MutY | 12 (9, 14) | 6.70 | 1.00 (NA, NA) | 79 |
| *Marinosulfonomonus* MutY | 7 (4, 10) | 4.13 | 0.57 (0.27, 0.91) | 30 |
| *Marinosulfonomonus* MutY (catalysis-) | 70 (51, 97) | 30.77 | 6.09 (4.32, 9.30) | 29 |
| *Marinosulfonomonus* MutY (recognition-) | 74 (59, 90) | 11.53 | 6.39 (4.69, 8.80) | 40 |
| *Rhodobacteraceae* MutY | 14 (8, 22) | 3.48 | 1.22 (0.71, 2.00) | 30 |
| *Rhodobacteraceae* MutY (catalysis-) | 61 (50, 80) | 41.37 | 5.26 (4.00, 8.00) | 38 |
| *Rhodobacteraceae* MutY (recognition-) | 70 (50, 82) | 37.65 | 6.09 (4.12, 8.75) | 30 |
| *Thiotrichaceae* MutY | 54 (42, 63) | 14.77 | 4.70 (3.23, 6.44) | 47 |
| *Thiotrichaceae* MutY (catalysis-) | 98 (50, 157) | 28.89 | 8.48 (4.46, 14.28) | 48 |
| *Thiotrichaceae* MutY (recognition-) | 72 (48, 183) | 10.75 | 6.26 (3.77, 18.00) | 20 |

[a] Confidence intervals (95%) determined by a bootstrap method, see Materials and Methods for details.
[b] Mutation frequency reported as median number of resistant colonies per $10^8$ viable colonies. Fold change was calculated by dividing $Rif^R$ frequency by the frequency measured for cultures expressing *Ec* MutY.
