# Supplementary material for: Metagenome mining and functional analysis reveal oxidized guanine DNA repair at the Lost City Hydrothermal Field
Source: PLoS One. 2024 May 8;19(5):e0284642. doi: 10.1371/journal.pone.0284642 (PMC11078426; doi:10.1371/journal.pone.0284642)
Supplement: S1 Movie — Structures for each MD trajectory were sampled at 200-ps intervals from 0–10,000 ps and at 1,000-ps intervals from 10,000–100,000 ps and movies were recorded with ChimeraX. Residues belonging to the NTD are depicted with a traditional ribbon style cartoon and colored light gray. Residues belonging to the CTD are depicted with a licorice cartoon style and colored dark gray. Solvent molecules that are within 4 Å of both the ligand and protein are shown (O, red; H, white). Each movie highlights particular features and events with time paused, the scene rotating about the y axis, and a brief caption. Molecular animations may be viewed via the YouTube playlist MD simulations for LCHF MutYs at the channel @biochemuu7993. (PDF) [file pone.0284642.s009.pdf]

S8 Movies. Molecular animations.
Structures for each MD trajectory were sampled at 200-ps intervals from 0 – 10,000 ps and at 1,000-ps intervals from 10,000 – 100,000 ps and movies were recorded with *ChimeraX*. Residues belonging to the NTD are depicted with a traditional ribbon style cartoon and colored light gray. Residues belonging to the CTD are depicted with a licorice cartoon style and colored dark gray. Solvent molecules that are within 4 Å of both the ligand and protein are shown (O, red; H, white). Each movie highlights particular features and events with time paused, the scene rotating about the y axis, and a brief caption. Molecular animations may be viewed via the YouTube playlist *MD simulations for LCHF MutYs* at the channel @biochemuu7993.

(A) Molecular animation for *Gs* MutY NTD complexed with adenosine. Adenosine remains within the active site pocket throughout the entire 100,000-ps simulation. At ~45,000 ps adenosine rotates within the active site to place its sugar in close proximity to the catalytic Glu43 residue, demonstrating a limited degree of flexibility within the active site pocket.

(B) Molecular animation for *Gs* MutY complexed with OG. OG remains wedged between NTD and CTD for most of the trajectory. Interactions with the Hoogsteen and Watson Crick face of OG relevant for OG recognition are highlighted at pauses. A new pose emerges at 90,000 ps just prior to departure of the ligand from the NTD-CTD interface.

(C) Molecular animation for *Marinosulfonomonas* MutY NTD complexed with adenosine. Adenosine slips back toward the entrance of the active site pocket within 1ns and exits completely by ~6,000-7,000 ps. It then settles in a pocket on the surface of the protein defined by the loop containing Ser24 and a helix from Ala58 to His65. It remains there until ~25,000 ps when it begins to move freely in the solvent, engaging, disengaging, then re-engaging with the surface of the protein for the remainder of the 100,000-ps simulation.

(D) Molecular animation for *Marinosulfonomonas* MutY complexed with OG. The two domains adopt a different disposition with a new inter-domain interface early in the simulation. The OG ligand finds two new binding sites on the NTD, each distinct from the original binding site, and persists complexed with the NTD until the end of the 100,000-ps simulation.

(E) Molecular animation for *Rhodobacteraceae* MutY NTD complexed with adenosine. Similar to the *Marinosulfonomonas* MutY NTD simulation, adenosine is completely outside the active site pocket relatively early in the simulation by ~5,000 ps. It then settles on the surface of the protein and wedges into a groove with residues Gly126 and Tyr128 on one side and Gln49 and Arg93 on the other side, and remains at this binding site for the rest of the 100,000-ps simulation.

(F) Molecular animation for *Rhodobacteraceae* MutY complexed with OG. The animation features a highly dynamic OG-MutY complex that dissociates completely by 48,000 ps. The OG ligand disengages from functionally relevant interactions at the NTD-CTD interface to find a new site on the NTD by 4,400 ps, nearly escapes at 13,000 ps, and samples several alternate sites on the NTD or the CTD or at a new site at the NTD-CTD interface prior to exiting this region

and exploring new sites on the surface of the NTD. The molecular animation is discontinued at 48,000 ps with the complex dissociated. The NTD-CTD structure remains intact for the remainder of the 100,000-ps simulation but the OG ligand did not rebind (not shown).

(G) Molecular animation for *Thiotrichaceae* MutY NTD complexed with adenosine. Adenosine remains in the active site pocket for the entire 100,000-ps simulation. Similarly to the *Gs* MutY-adenosine simulation, the ligand rotates within the active site pocket at ~43,000 ps to place its sugar within close proximity of the active site Glu46 residue, demonstrating limited flexibility within the active site pocket.

(H) Molecular animation for *Thiotrichaceae* MutY complexed with OG. The complex persists for the entire 100,00-ps simulation. The initial complex features interaction of Ser306 with the Watson-Crick-Franklin face of the OG base. This pose persists until transition to a new pose at ~69,000 ps with the deoxyribose sugar closer to Ser306 and the base wedged between two helixes that converge at the NTD-CTD interface.

(I) Molecular animation for *Flavobacteriaceae* MutY NTD complexed with adenosine. Adenosine remains within the active site pocket for the entire 100,000 ps, It starts with its sugar facing the catalytic Glu33 residue. At ~3,000 ps it rotates to bring the base portion deeper within the active site. It remains in this general orientation for the remainder of the 100,000 ps with the sugar engaging Glu33 in the ~60,000 – 80,000-ps time window.

(J) Molecular animation for *Flavobacteriaceae* MutY complexed with OG. During the first 9,800 ps Ser305 makes hydrogen bonds with the Hoogsteen face of OG in a manner relevant for recognition. At 10,000 ps a new pose emerges with the base wedged between helices in the NTD and CTD and thus removed from the FSH recognition loop. The complex with this new pose persists for the remainder of the 100,000-ps simulation.
