# Supplementary material for: Metagenome mining and functional analysis reveal oxidized guanine DNA repair at the Lost City Hydrothermal Field
Source: PLoS One. 2024 May 8;19(5):e0284642. doi: 10.1371/journal.pone.0284642 (PMC11078426; doi:10.1371/journal.pone.0284642)
Supplement: Attachment — Submitted filename: Response to Reviewers for second revision.docx [file pone.0284642.s021.docx]

## Response to Reviewers and Editor Comments

Responses from the authors are provided for each point raised in the initial and second review. The responses for the first review are highlighted in blue; responses for the second review are highlighted in red. Direction as to location for additions or changes have been updated to reflect the line positions in the current, second revision of the manuscript.

## Second Review

Additional Editor Comments:

**MD simulation which is an important part to the research work, but here the time consideration only for 10-nano second i.e. a very small time limit. Again the authors have mentioned in result and supportive data that the study outcome is unstable in majority of cases.**

**As a suggestion the time limit should be minimum 100 ns or more, which will give us more data to analyze it in depth.**

Thank you for this opportunity to extend the MD analysis. The current, revised manuscript describes 100-ns MD trajectories for all 10 enzyme-ligand complexes. The more extensive analyses reinforce ideas from the shorter treatments and provide a more complete sense of dynamic properties. The text, figure and tables describing results from the MD simulations have been updated. Please see Table 3 (see Lines 327-343), Results section Molecular Dynamics (see Lines 368-438), Materials and Methods (see Lines 690, 701, 705-706), Supporting Information Figure S7 (see Lines 1103-1169) and Supporting Information Movie S8 (see Lines 1171-1226). To better illustrate the structures encountered, we have built molecular animations with certain events highlighted by pauses in time. These movies can be downloaded from the link provided here: [Movies PLOS ONE](https://drive.google.com/drive/folders/1xlm3MhU9fr6UN-q7vYTMbyy8mqCB3YNE?usp=drive_link)

The study reveals instability in many cases, but the degree of instability correlates positively with biological activity under mesophile conditions. This compelling finding speaks to the idea of “corresponding states” as described in the initially revised Discussion section. “Corresponding states” hypothesizes that enzyme flexibility is necessary for function and therefore adapts to conditions. Enzymes adapted to extreme temperatures are flexible at high temperatures to the same degree as mesophile enzymes are at moderate temperatures, and therefore function poorly at lower temperatures due to rigidity. Together, the molecular modeling and biological function assay reinforce the idea that some of the microbes inhabiting the Lost City Hydrothermal Field are adapted to extreme conditions.

The current, second revised manuscript makes these points regarding general instability and its correlation with biological activity more clear by adding text in the Results section Molecular Dynamics (see Lines 375-379, 433-438) and the Results section Testing mutation suppression activity (see Lines 467-468).

## Initial Review

When submitting your revision, we need you to address these additional requirements.

1. Please ensure that your manuscript meets PLOS ONE's style requirements, including those for file naming. The PLOS ONE style templates can be found at
https://journals.plos.org/plosone/s/file?id=wjVg/PLOSOne_formatting_sample_main_body.pdf and
https://journals.plos.org/plosone/s/file?id=ba62/PLOSOne_formatting_sample_title_authors_affiliations.pdf

The authors have carefully reviewed the PLOS ONE style requirements, including those for file naming, and the revised version matches these style requirements as far as we know.

2. Thank you for stating in your Funding Statement:
"This work was supported by National Science Foundation (https://www.nsf.gov/) awards to Martin. P Horvath (CHE:CLP- 1905249, 2204229) and to William J. Brazelton. (OCE-1536405). This work was also supported by the NASA Astrobiology Institute Rock-Powered Life team."

Please provide an amended statement that declares *all* the funding or sources of support (whether external or internal to your organization) received during this study, as detailed online in our guide for authors at http://journals.plos.org/plosone/s/submit-now. Please also include the statement “There was no additional external funding received for this study.” in your updated Funding Statement.
Please include your amended Funding Statement within your cover letter. We will change the online submission form on your behalf.

The funding statement as been amended as requested, added to the cover letter, and this statement is also provided here. Thank you for updating the online submission form. That is a big help.

Updated Funding Statement:

This work was supported by NSF awards to MPH (CHE:CLP- 1905249, 2204229) and to WJB (OCE-1536405), by the NASA Astrobiology Institute Rock-Powered Life team, and by UROP funding from the Office of Undergraduate Research at the University of Utah awarded to PHU. The support and resources from the Center for High Performance Computing at the University of Utah are gratefully acknowledged. There was no additional external funding received for this study.

End of Updated Funding Statement

3. Please amend your manuscript to include your abstract after the title page.

The Abstract is now included after the title page.

Additional Editor Comments:

**The author should confirm the similarity and identity between the query sequence and database sequences in BLAST research**

Supporting Information **Table S2** reports the pairwise identity among query and database sequences. The text clarifies the % identity cutoff for finding MutY candidate genes in the metagenome and the E-value threshold (see Lines 162-163). Additionally, observations regarding % identity between candidate genes and query sequences are included in the text (see Lines 233-238)

**In case of protein structure prediction, how the authors have assessed the modeled strucrture quality, it should be framed appropriatey and added in the text.**

**Table 3** now reports the pLDDT scores for each predicted structure. Notes for assessing modeled structure quality have also been added to the text (see Lines 307-308, 327-343)

**As the Authors have performed molecular docking experiment, what was the rational to select the binding site region, kindly add it**.

The text now describes the rationale for selection of the binding site region in the Results section (see Lines 351-353)

**The molecular docking is not the wholesome validation, the authors should carry out MD Simulation study, which will give us more detail outcome in terms of dynamicity and stability.**

Thank you for this suggestion to validate the molecular docking outcomes. The report now includes MD simulation to analyze the docking outcomes. Interaction energies averaged over a 10-ns simulation are reported in **Table 3** for each ligand-receptor complex. The text also describes these outcomes in a new subsection **Molecular dynamics simulations** (see Lines 361-448). Supporting Information **Fig S7** shows time-evolution structures along with time-dependent distance and energy plots.

Spell check and grammar needs to corrected

Spelling and grammar have been corrected.

The PDB-id should be well define.

PDB-ids have been defined.

[Note: HTML markup is below. Please do not edit.]

Reviewers' comments:

Reviewer's Responses to Questions

**Comments to the Author**

1. Is the manuscript technically sound, and do the data support the conclusions?

The manuscript must describe a technically sound piece of scientific research with data that supports the conclusions. Experiments must have been conducted rigorously, with appropriate controls, replication, and sample sizes. The conclusions must be drawn appropriately based on the data presented.

Reviewer #1: Yes

2. Has the statistical analysis been performed appropriately and rigorously?

Reviewer #1: Yes

3. Have the authors made all data underlying the findings in their manuscript fully available?

The [PLOS Data policy](http://www.plosone.org/static/policies.action#sharing) requires authors to make all data underlying the findings described in their manuscript fully available without restriction, with rare exception (please refer to the Data Availability Statement in the manuscript PDF file). The data should be provided as part of the manuscript or its supporting information, or deposited to a public repository. For example, in addition to summary statistics, the data points behind means, medians and variance measures should be available. If there are restrictions on publicly sharing data—e.g. participant privacy or use of data from a third party—those must be specified.

Reviewer #1: Yes

4. Is the manuscript presented in an intelligible fashion and written in standard English?

PLOS ONE does not copyedit accepted manuscripts, so the language in submitted articles must be clear, correct, and unambiguous. Any typographical or grammatical errors should be corrected at revision, so please note any specific errors here.

Reviewer #1: Yes

5. Review Comments to the Author

Please use the space provided to explain your answers to the questions above. You may also include additional comments for the author, including concerns about dual publication, research ethics, or publication ethics. (Please upload your review as an attachment if it exceeds 20,000 characters)

Reviewer #1: This well-written and enjoyable manuscript by Utzman et al provides compelling evidence that organisms evolved ways to deal with oxidative DNA damage prior to the Great Oxidation Event. The presence of homologues of the GO DNA repair system in microbes recovered from the Lost City Hydrothermal Field (LCHF) is interesting, and the ability of distant mutY homologues from this environment to complement E. coli mutY mutants is remarkable. This work raises a number of interesting possibilities concerning the importance of ROS and oxidative damage during the evolution of life, even in the absence of high oxygen levels.

Thank you for reviewing our manuscript favorably!

I have only a couple of minor notes:

1. In the Discussion, when covering potential sources of ROS/RNS/RSS, beyond being by-products of some metabolic pathways, might these also be generated as a form of inter-bacterial competition or warfare? The idea that many antibiotics act by generating ROS directly or indirectly is gaining traction and might be relevant selective pressures in mixed microbial communities. Also of potential relevance is the idea that some quorum sensing molecules might be progenitors of certain antibiotics, having antibiotic activities against competitors. Is there any evidence of inter-microbial competition/warfare within the LCHF or similar environments?

Thank you for this interesting observation and suggestion. We have added note in the Discussion to highlight the possibility of bacterial warfare (see Lines 563-565); however, so far no evidence for inter-bacterial competition has emerged from analysis of these communities.

2. Line 174: '…and the catalytic Tyr residue is often replaced...' (missing ‘d’)

Thank you for pointing out this typographical error which has been corrected (see Line 191). Other mistakes in spelling and grammar have also been corrected.
